# Supplementary material for: Bacteriophage infection drives loss of β-lactam resistance in methicillin-resistant Staphylococcus aureus
Source: eLife. 2025 Jul 10;13:RP102743. doi: 10.7554/eLife.102743 (PMC12245174; doi:10.7554/eLife.102743)

Figure 1–Source Data 1

For Figure 1B: Plaquing images of bacteriophage ΦStaph1N against parental MRSA strains MRSA252, MW2, and LAC.

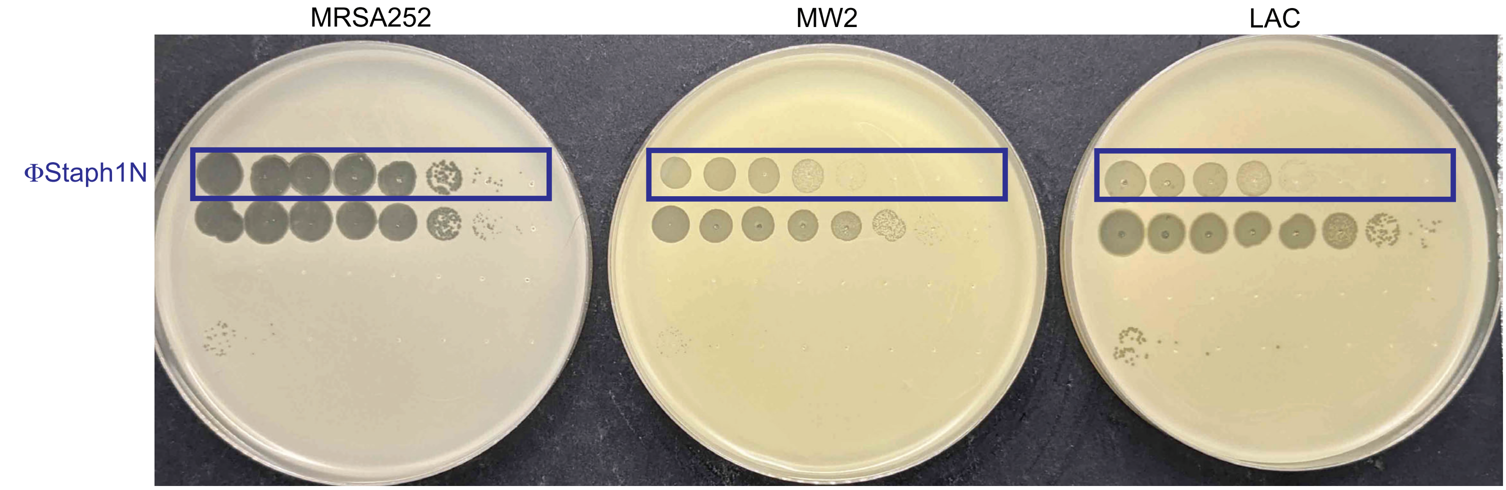

For Figure 1B: Plaquing images of bacteriophage ΦStaph1N against evolved MRSA strains MRSA252, MW2, and LAC.

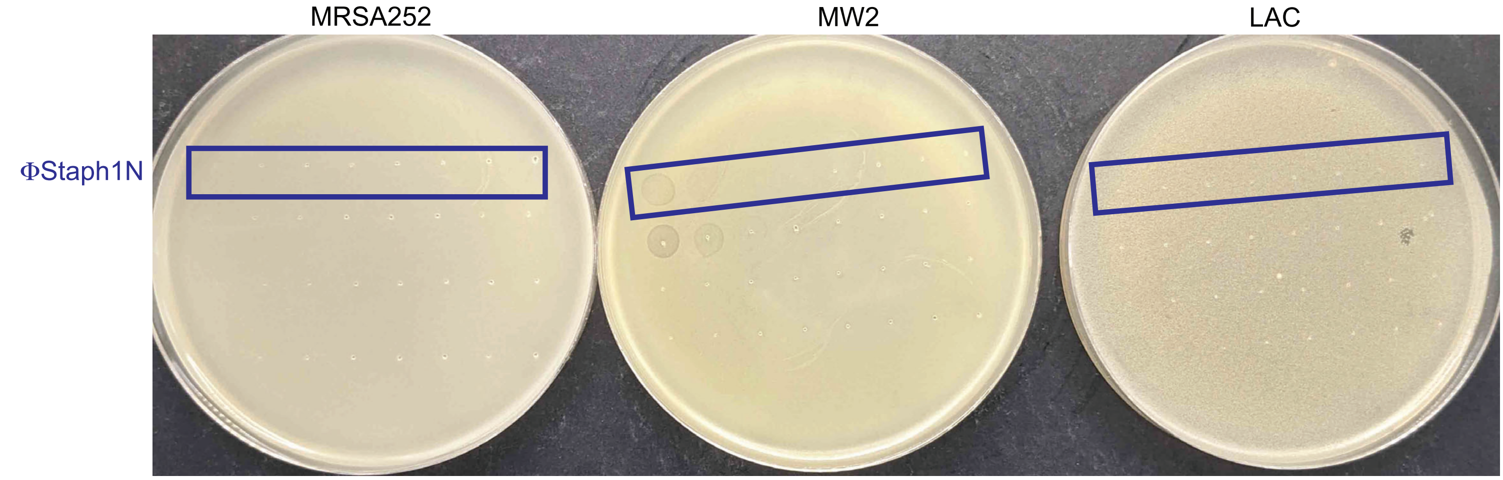

For Figure 1C: Uncropped images of parental and evolved MRSA strains exposed to oxacillin strips.

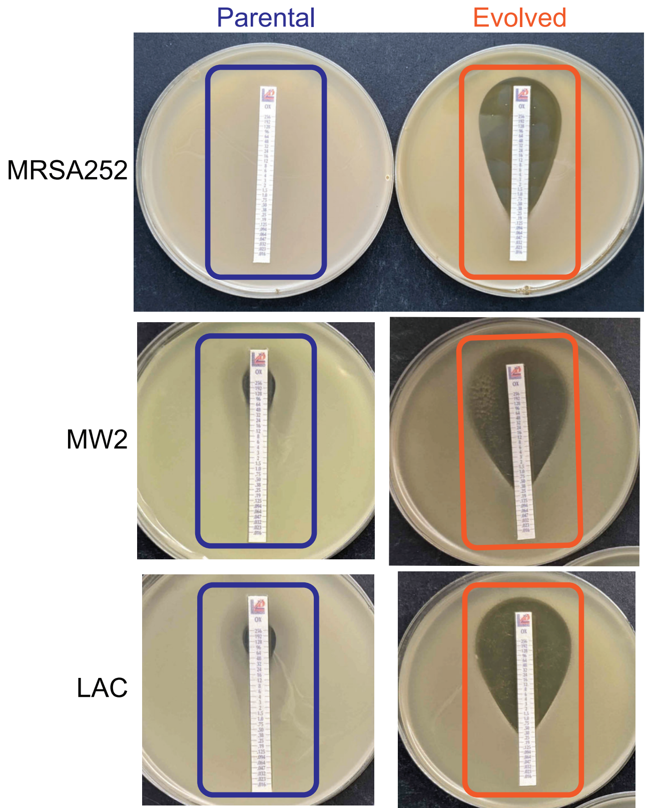

Supplement: Figure 1—source data 1. [file elife-102743-fig1-data1.zip › Figure1_Source Data 1/Figure1-Source Data1.pdf]
