# Supplementary figures and images for: Bacteriophage infection drives loss of β-lactam resistance in methicillin-resistant Staphylococcus aureus
Source: eLife. 2025 Jul 10;13:RP102743. doi: 10.7554/eLife.102743 (PMC12245174; doi:10.7554/eLife.102743)

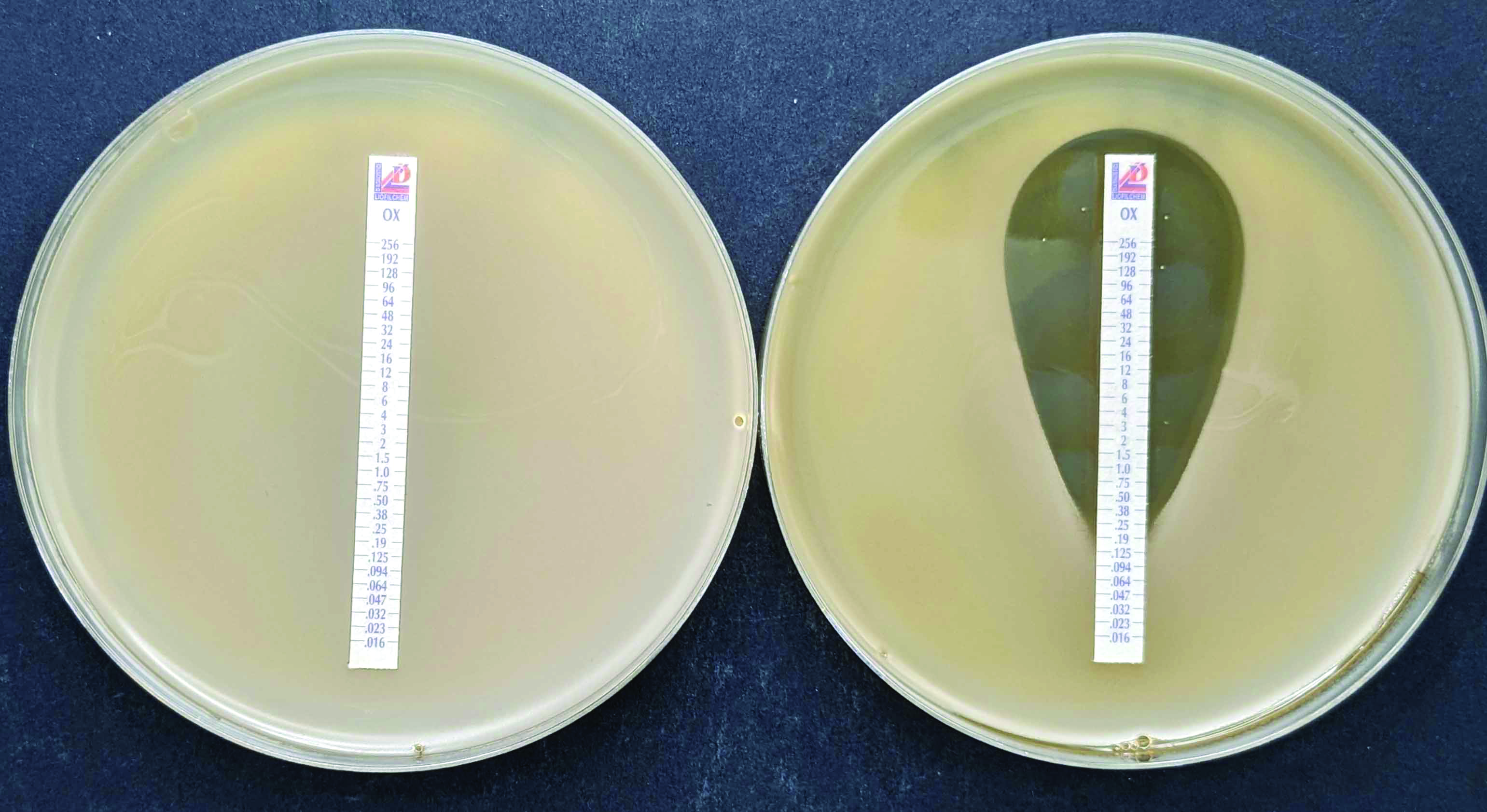

Supplement: Figure 1—source data 1. [file elife-102743-fig1-data1.zip › Figure1_Source Data 1/Figure1_sourcedata_rawimage3.tif]

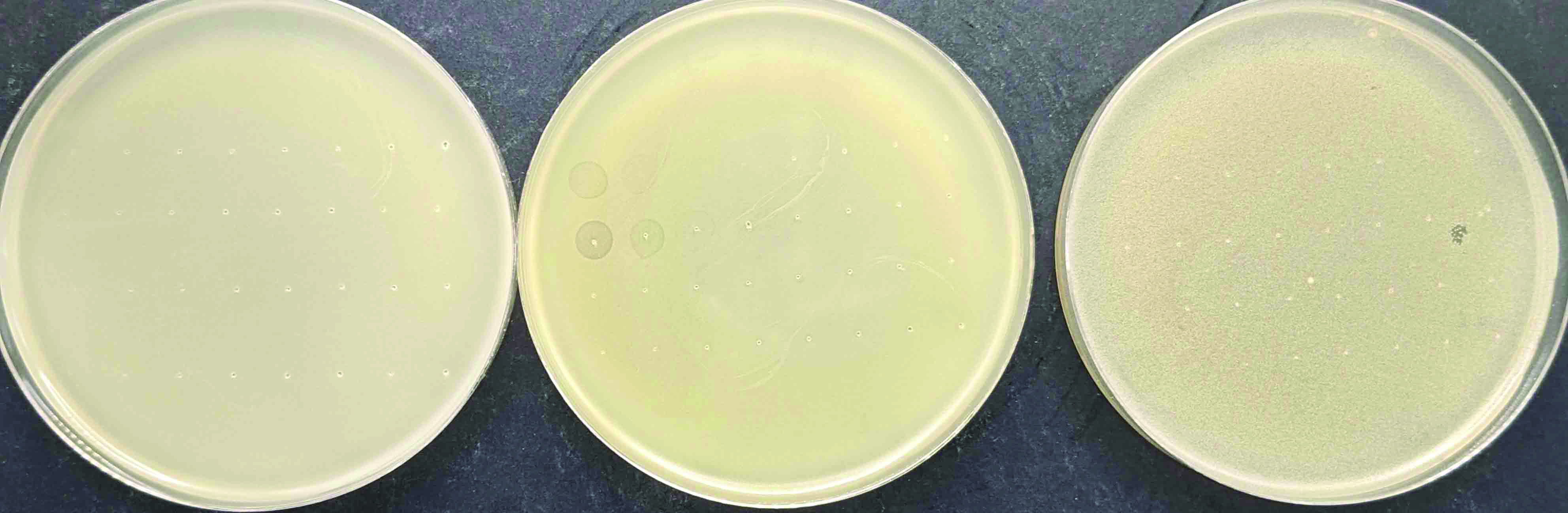

Supplement: Figure 1—source data 1. [file elife-102743-fig1-data1.zip › Figure1_Source Data 1/Figure1_sourcedata_rawimage2.tif]

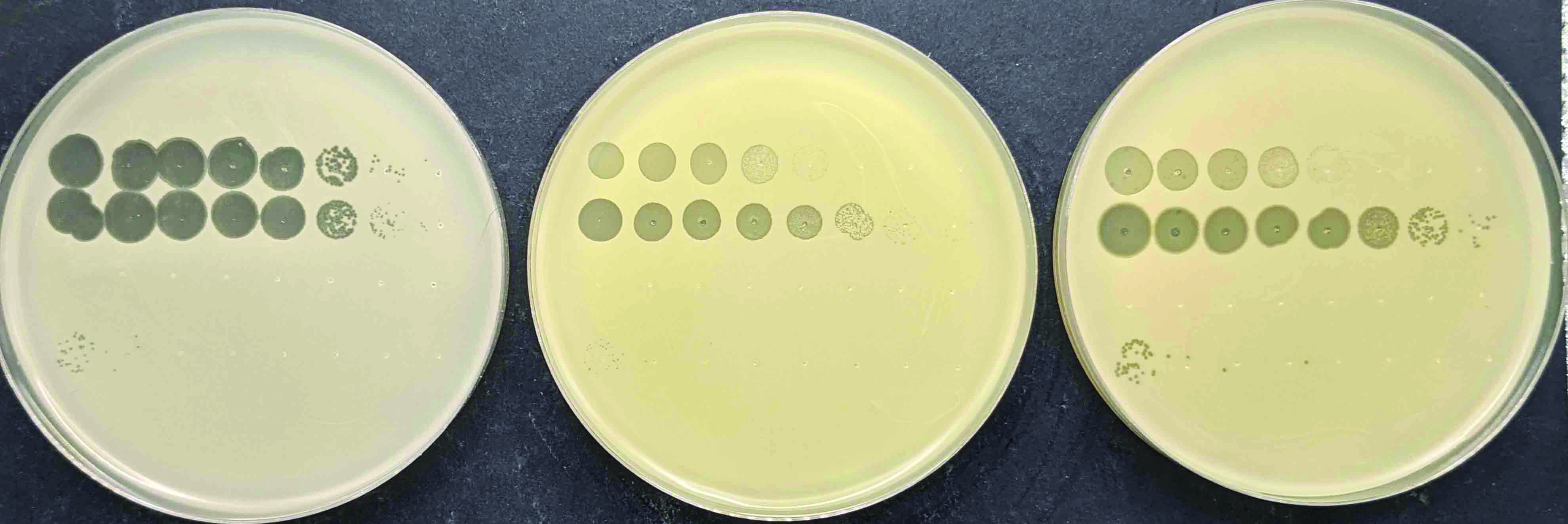

Supplement: Figure 1—source data 1. [file elife-102743-fig1-data1.zip › Figure1_Source Data 1/Figure1_sourcedata_rawimage1.tif]

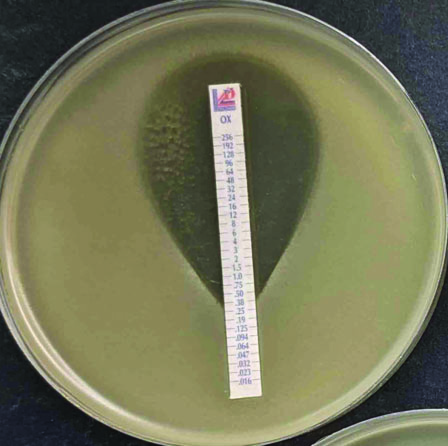

Supplement: Figure 1—source data 1. [file elife-102743-fig1-data1.zip › Figure1_Source Data 1/Figure1_sourcedata_rawimage5.tif]

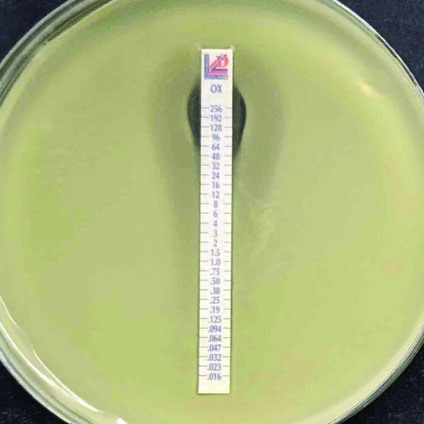

Supplement: Figure 1—source data 1. [file elife-102743-fig1-data1.zip › Figure1_Source Data 1/Figure1_sourcedata_rawimage4.tif]

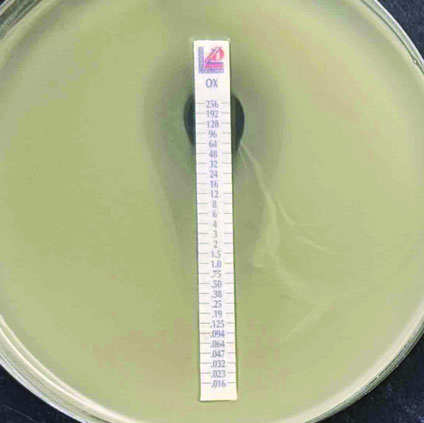

Supplement: Figure 1—source data 1. [file elife-102743-fig1-data1.zip › Figure1_Source Data 1/Figure1_sourcedata_rawimage6.tif]

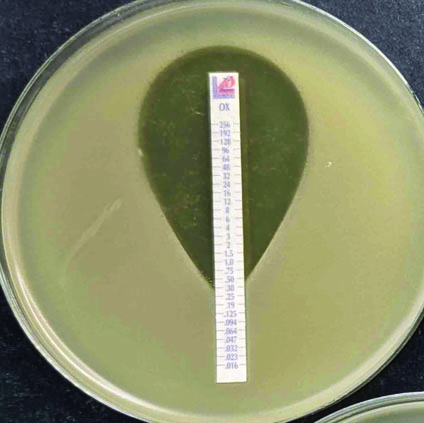

Supplement: Figure 1—source data 1. [file elife-102743-fig1-data1.zip › Figure1_Source Data 1/Figure1_sourcedata_rawimage7.tif]

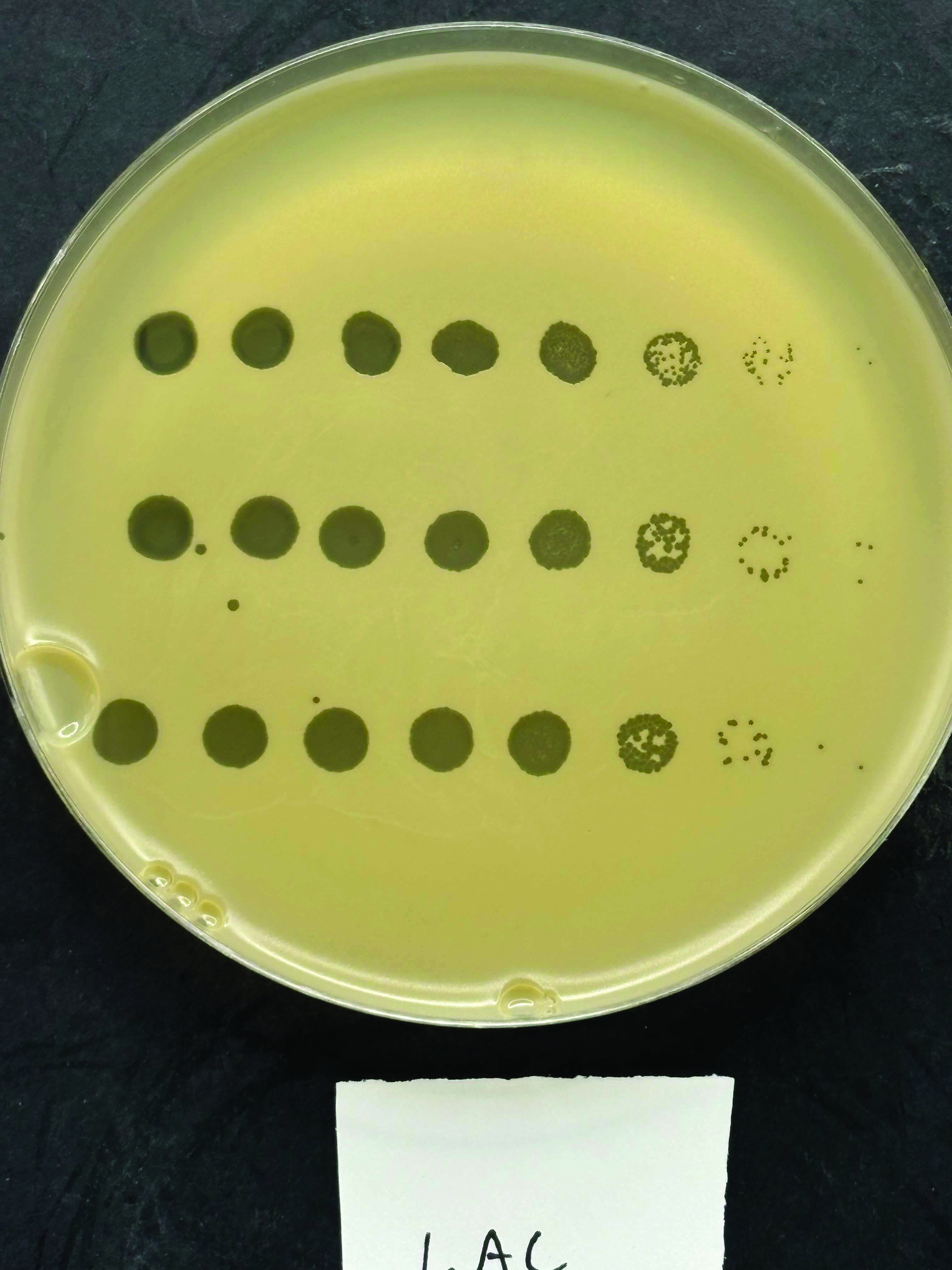

Supplement: Figure 1—figure supplement 1—source data 1. [file elife-102743-fig1-figsupp1-data1.zip › Figure1-figure supplement1_Source Data 1/Figure1_figure supplement 1_Source Data_rawimage5 copy.jpg]

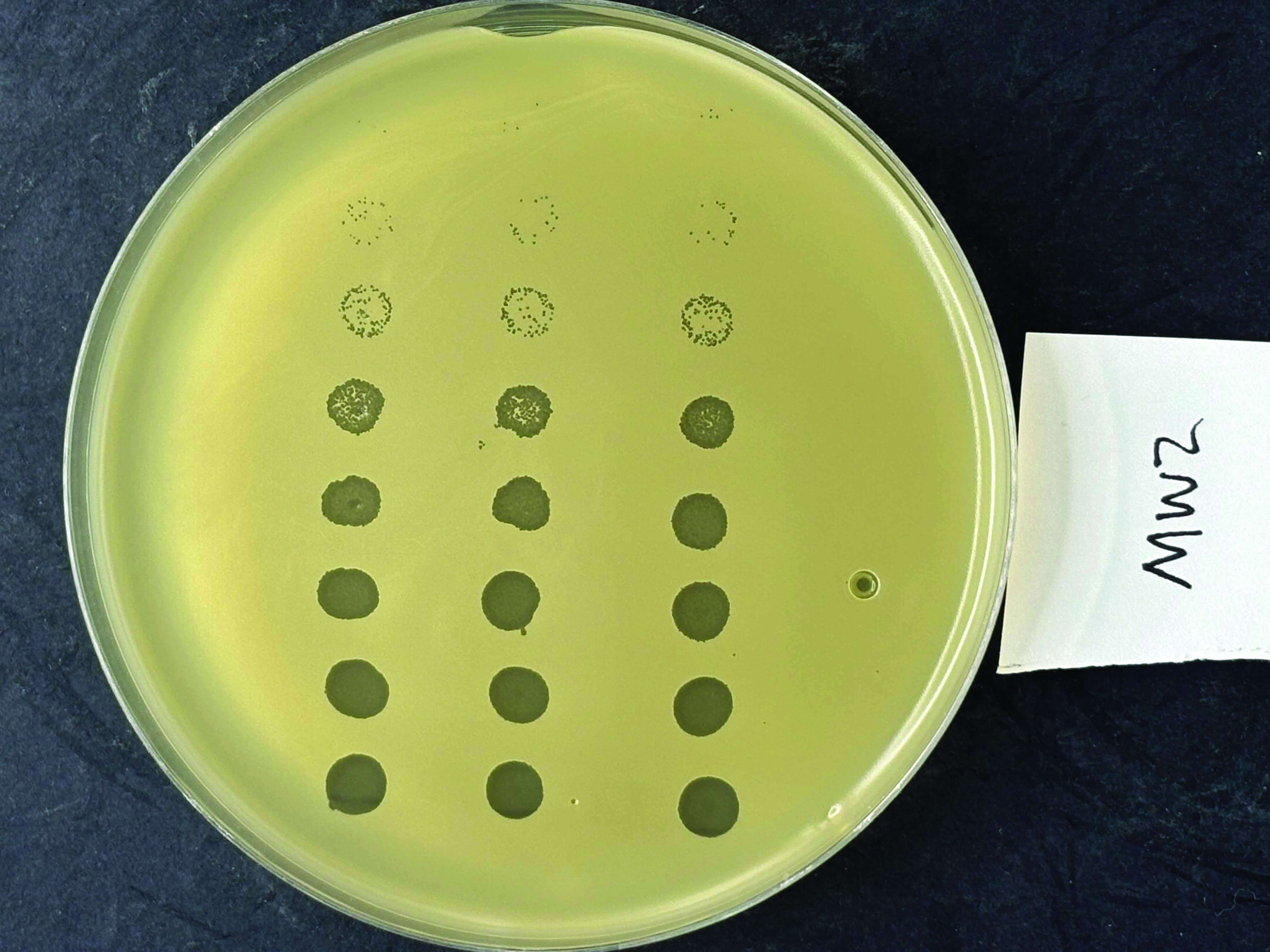

Supplement: Figure 1—figure supplement 1—source data 1. [file elife-102743-fig1-figsupp1-data1.zip › Figure1-figure supplement1_Source Data 1/Figure1_figure supplement 1_Source Data_rawimage4 copy.jpg]

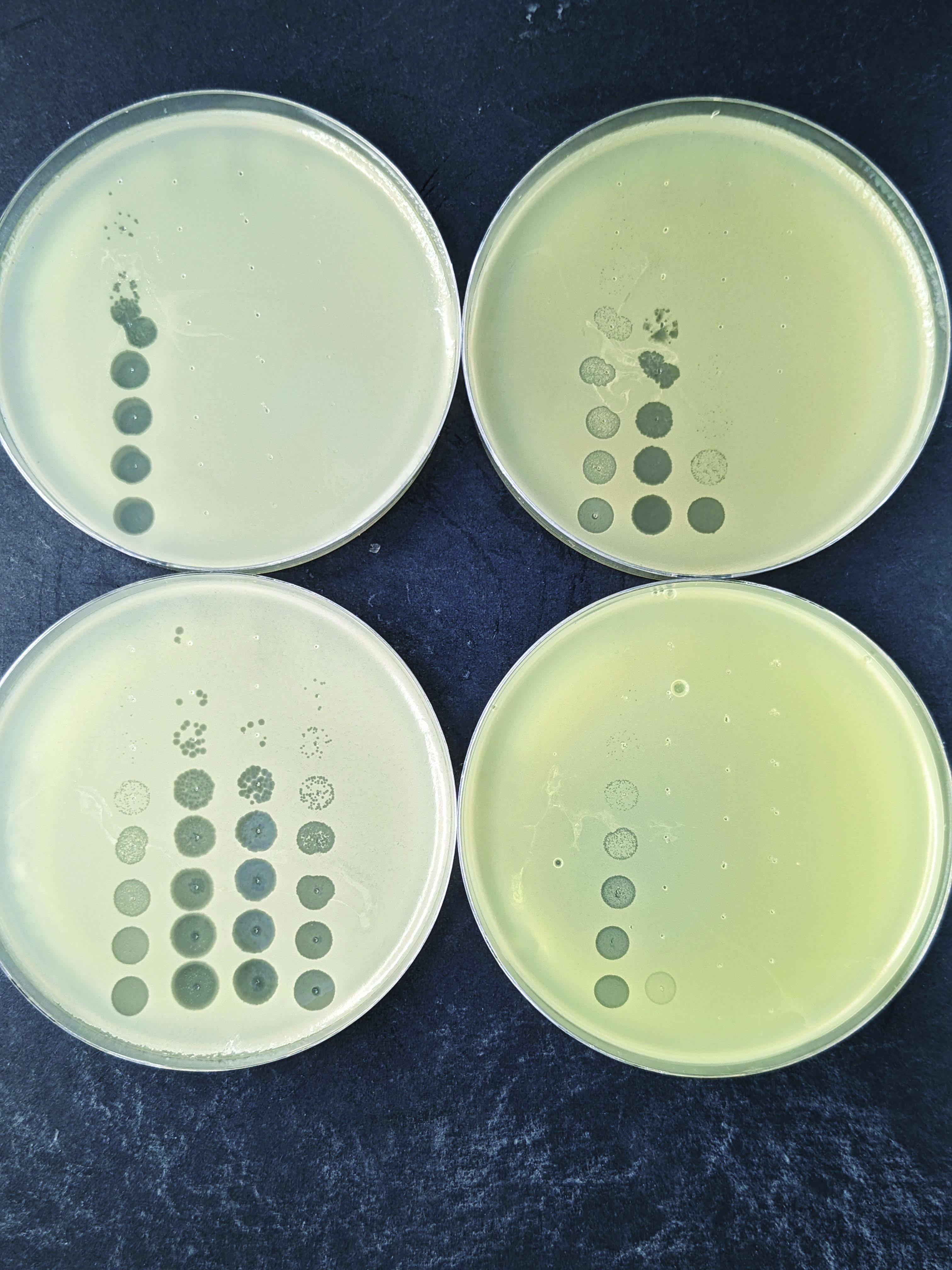

Supplement: Figure 1—figure supplement 1—source data 1. [file elife-102743-fig1-figsupp1-data1.zip › Figure1-figure supplement1_Source Data 1/Figure1_figure supplement 1_Source Data_rawimage2 copy.jpg]

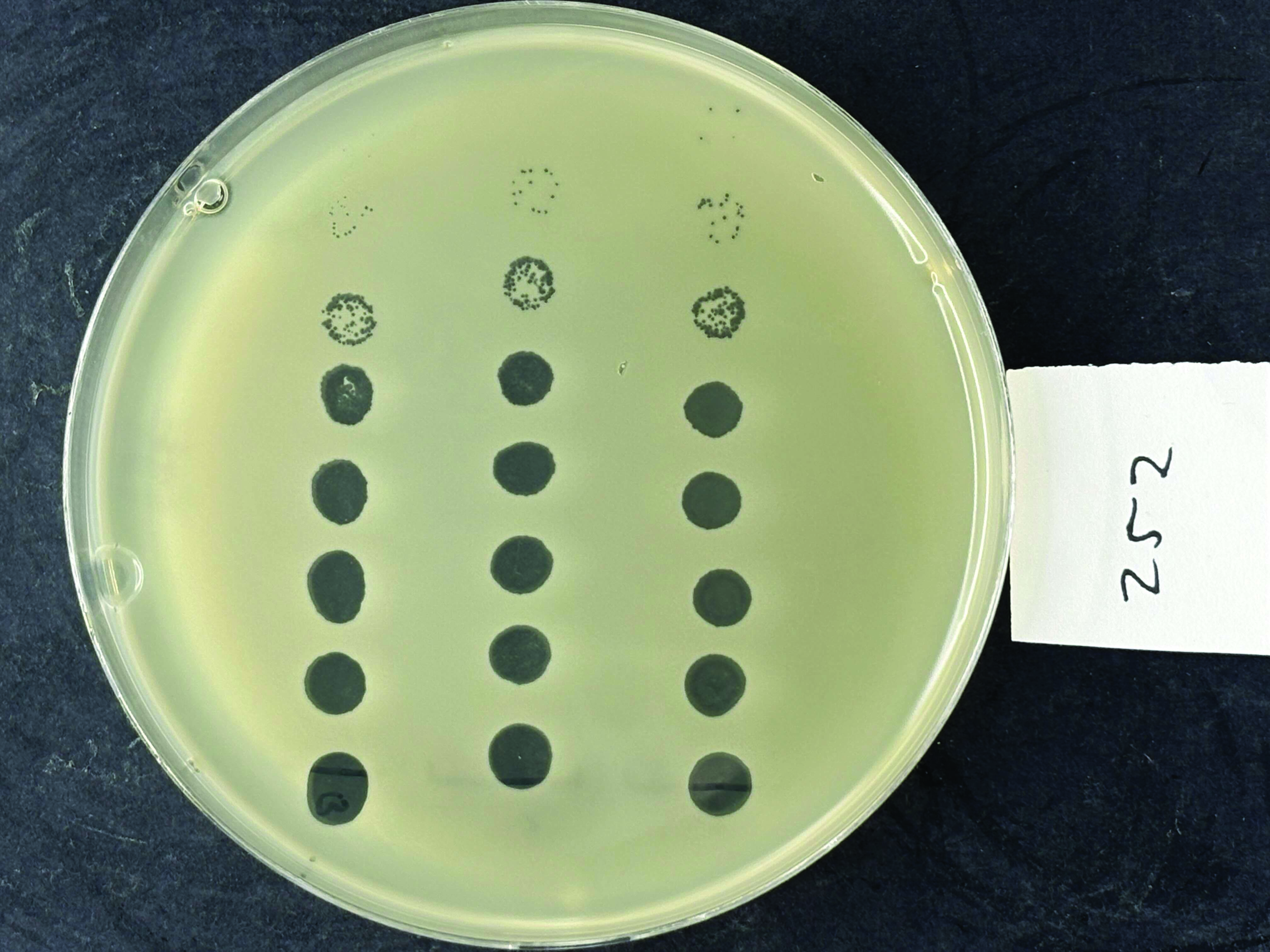

Supplement: Figure 1—figure supplement 1—source data 1. [file elife-102743-fig1-figsupp1-data1.zip › Figure1-figure supplement1_Source Data 1/Figure1_figure supplement 1_Source Data_rawimage3 copy.jpg]

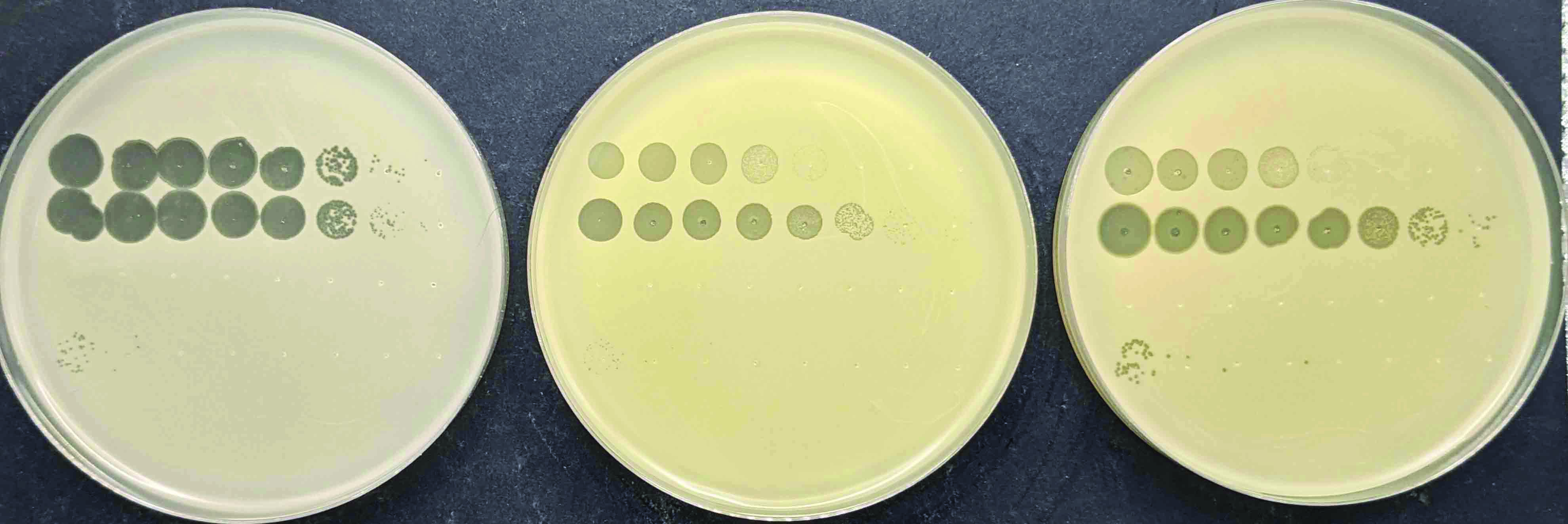

Supplement: Figure 1—figure supplement 1—source data 1. [file elife-102743-fig1-figsupp1-data1.zip › Figure1-figure supplement1_Source Data 1/Figure1_figure supplement 1_Source Data_rawimage1 copy.jpg]

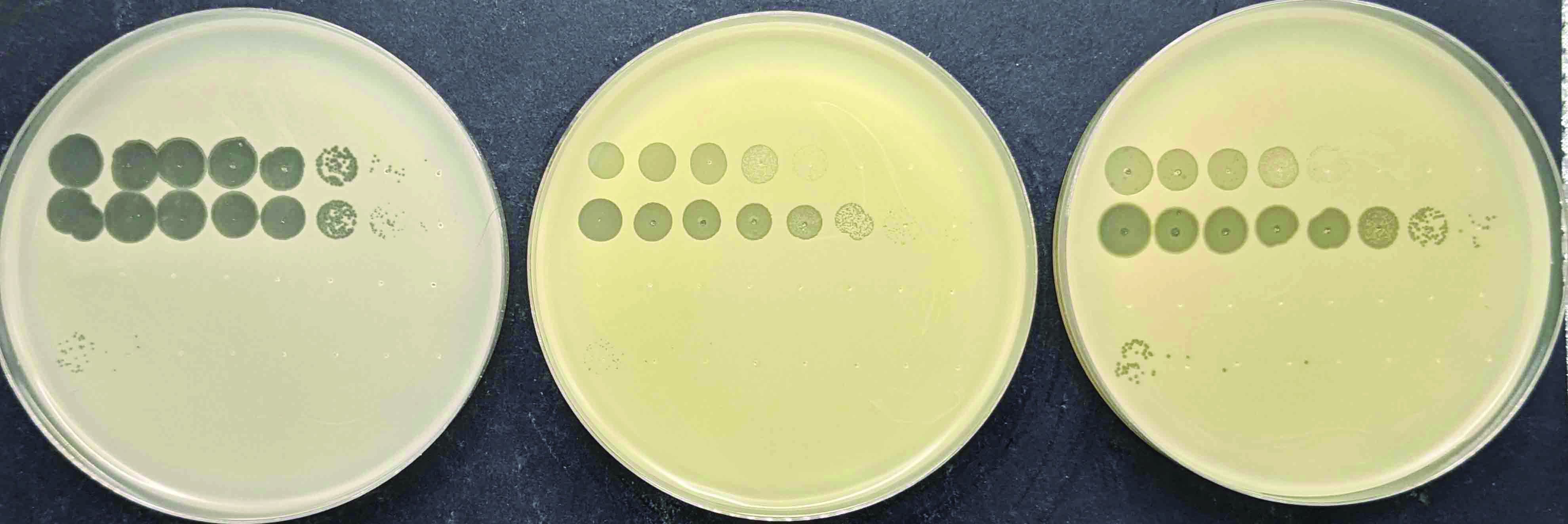

Supplement: Figure 2—source data 1. [file elife-102743-fig2-data1.zip › Figure 2_Source Data 1/Figure 2_Source Data_rawimage1.tif]

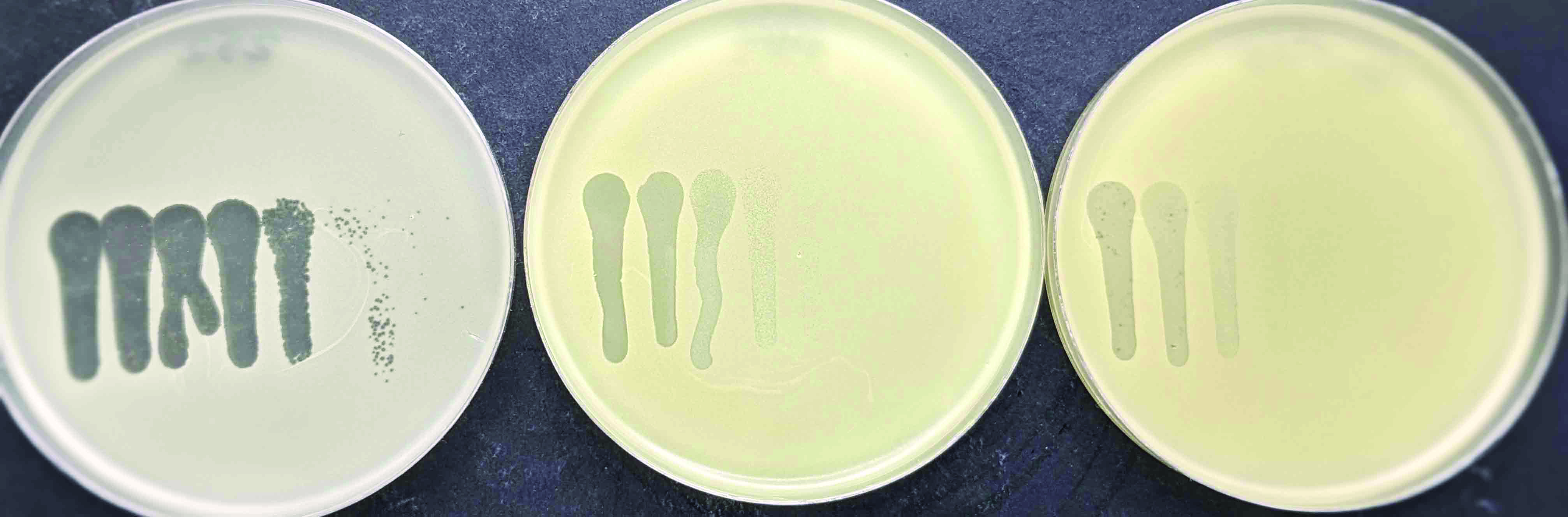

Supplement: Figure 2—figure supplement 1—source data 1. [file elife-102743-fig2-figsupp1-data1.zip › Figure 2-figure supplement 1_Source Data 1/Figure2_figure supplement 1_rawimage1.tif]

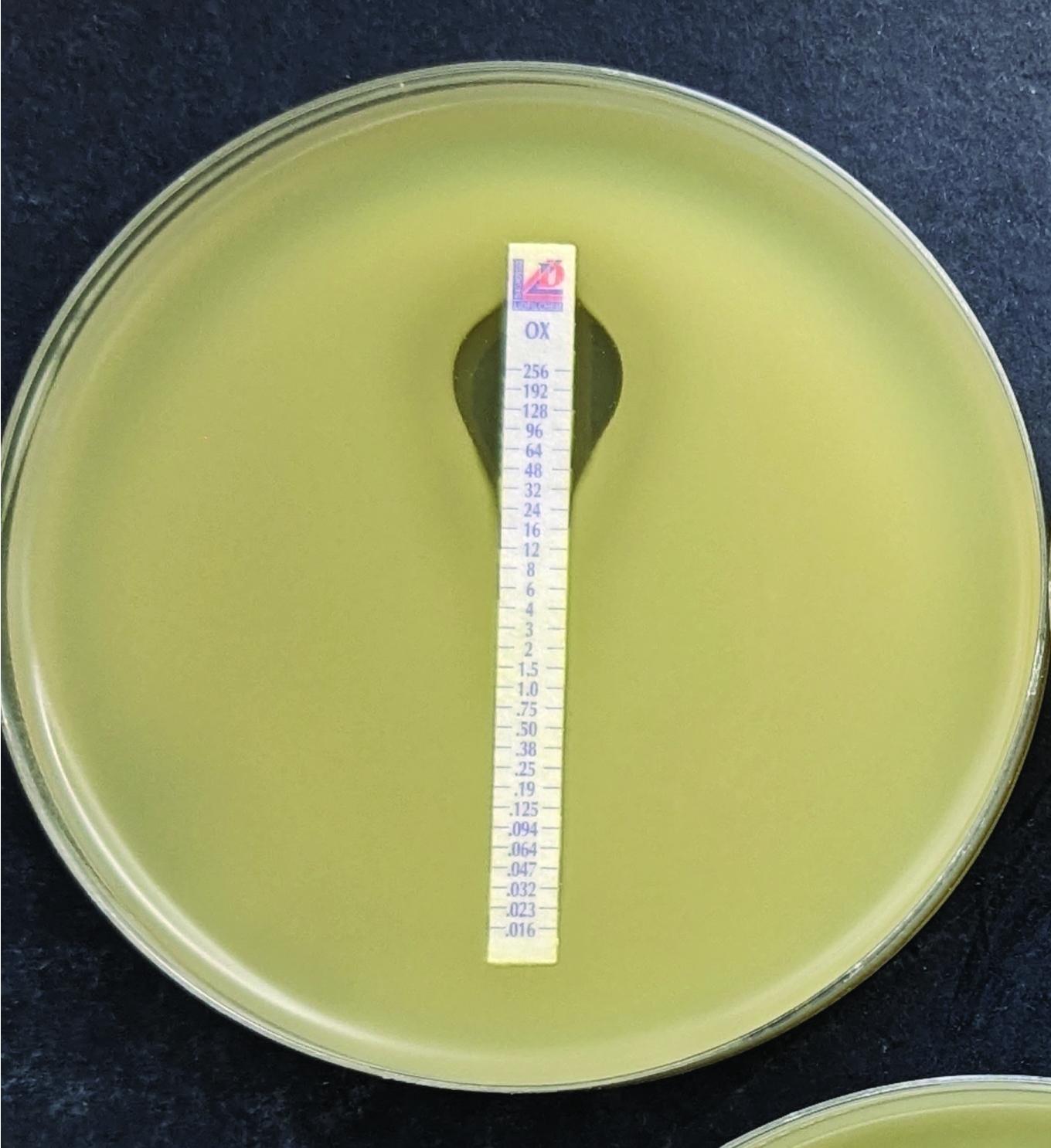

Supplement: Figure 2—figure supplement 3—source data 1. [file elife-102743-fig2-figsupp3-data1.zip › Figure 2-figure supplement 3_Source Data 1/Figure 2-figure supplement 3-rawimage8.tif]

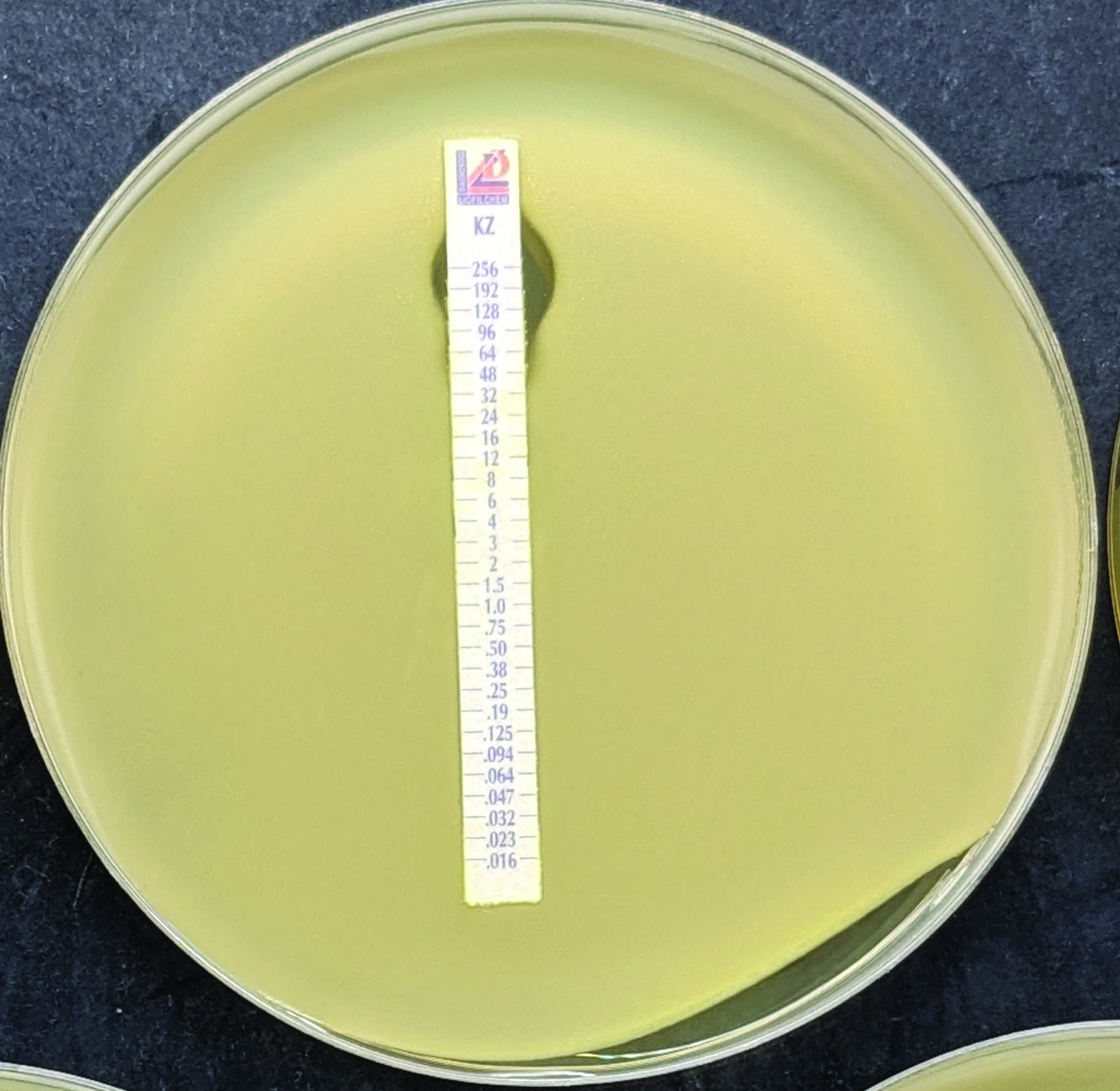

Supplement: Figure 2—figure supplement 3—source data 1. [file elife-102743-fig2-figsupp3-data1.zip › Figure 2-figure supplement 3_Source Data 1/Figure 2-figure supplement 3-rawimage9.tif]

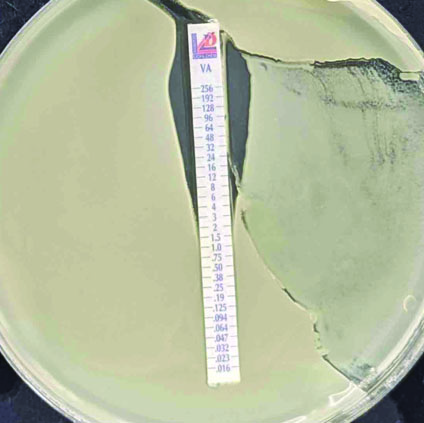

Supplement: Figure 2—figure supplement 3—source data 1. [file elife-102743-fig2-figsupp3-data1.zip › Figure 2-figure supplement 3_Source Data 1/Figure 2-figure supplement 3-rawimage7.tif]

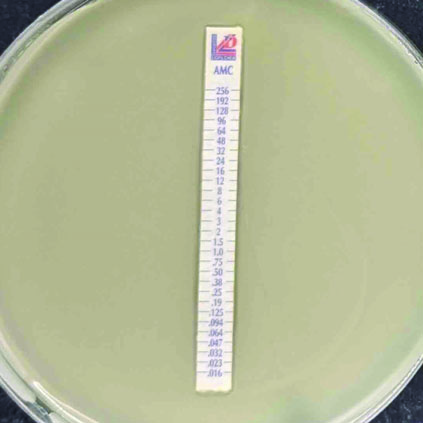

Supplement: Figure 2—figure supplement 3—source data 1. [file elife-102743-fig2-figsupp3-data1.zip › Figure 2-figure supplement 3_Source Data 1/Figure 2-figure supplement 3-rawimage6.tif]

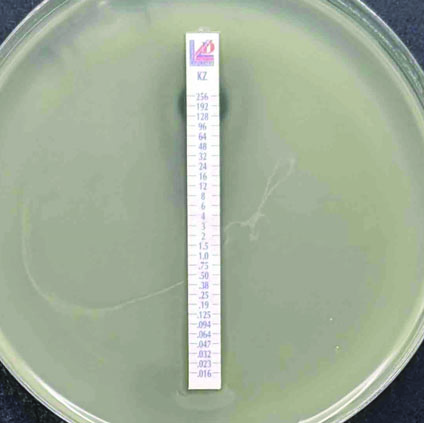

Supplement: Figure 2—figure supplement 3—source data 1. [file elife-102743-fig2-figsupp3-data1.zip › Figure 2-figure supplement 3_Source Data 1/Figure 2-figure supplement 3-rawimage4.tif]

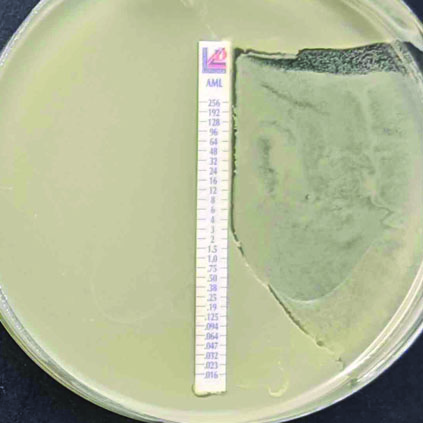

Supplement: Figure 2—figure supplement 3—source data 1. [file elife-102743-fig2-figsupp3-data1.zip › Figure 2-figure supplement 3_Source Data 1/Figure 2-figure supplement 3-rawimage5.tif]

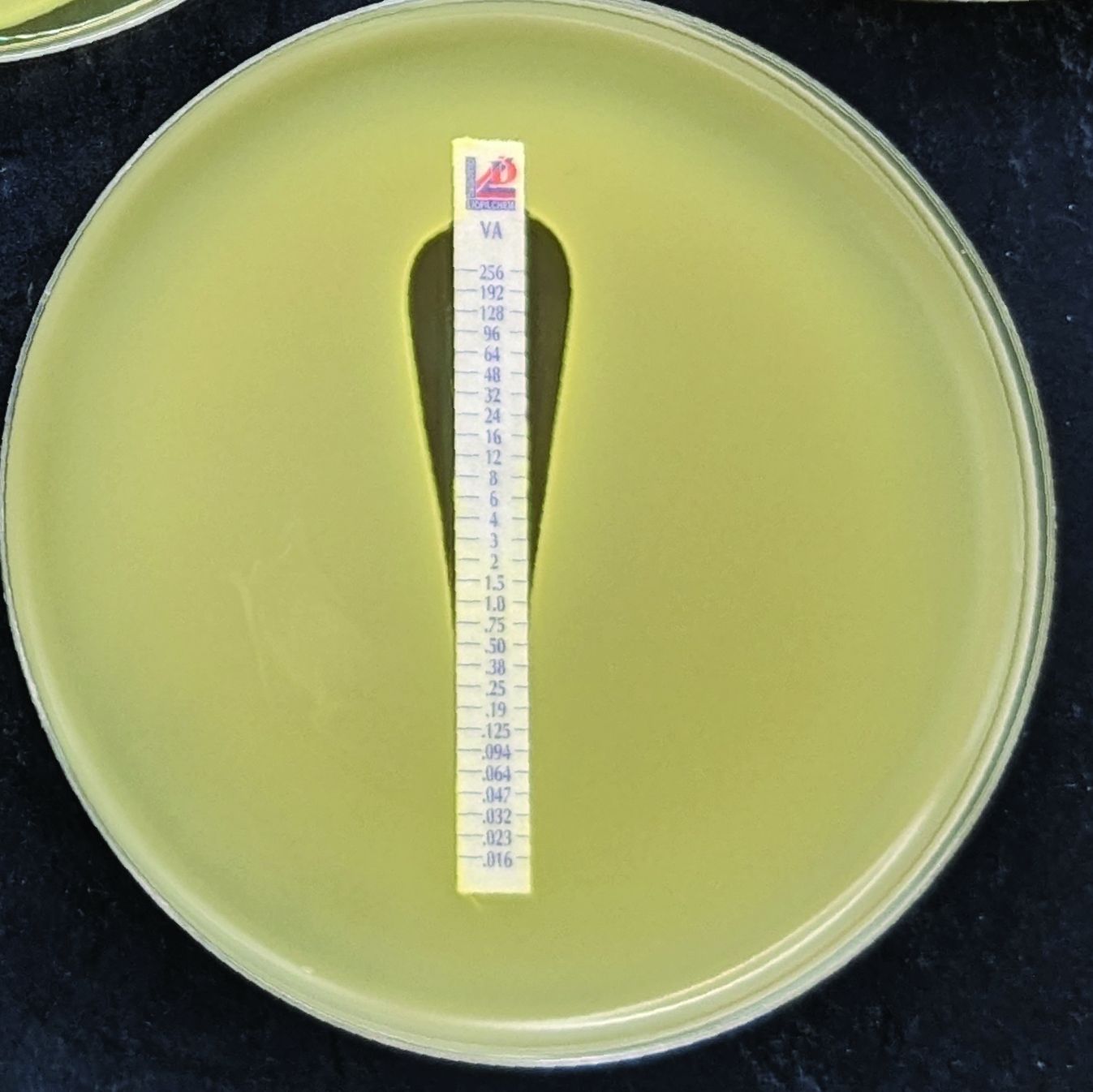

Supplement: Figure 2—figure supplement 3—source data 1. [file elife-102743-fig2-figsupp3-data1.zip › Figure 2-figure supplement 3_Source Data 1/Figure 2-figure supplement 3-rawimage12.tif]

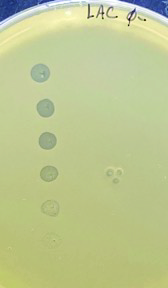

Supplement: Figure 2—figure supplement 3—source data 1. [file elife-102743-fig2-figsupp3-data1.zip › Figure 2-figure supplement 3_Source Data 1/Figure 2-figure supplement 3-rawimage1.tif]

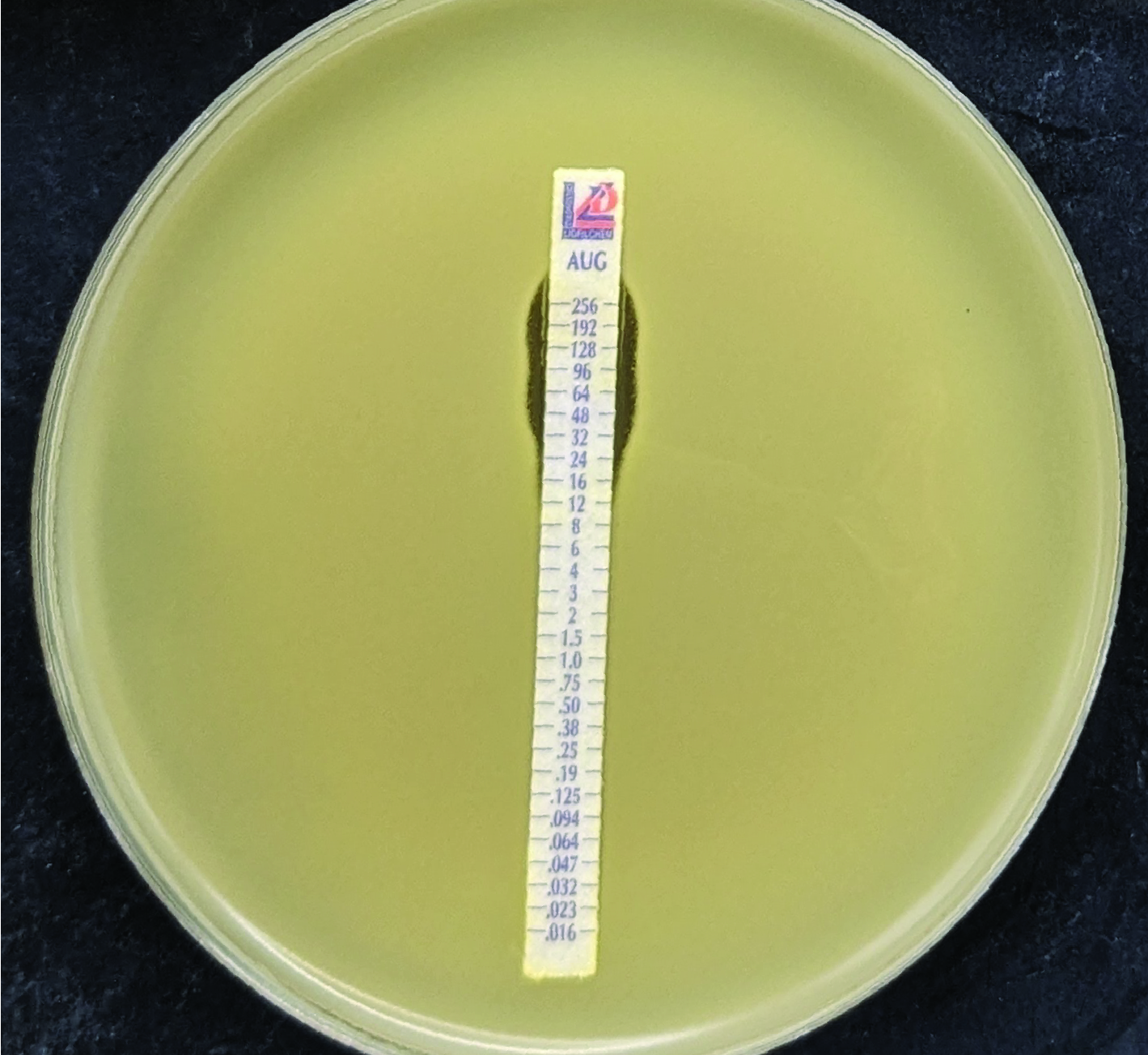

Supplement: Figure 2—figure supplement 3—source data 1. [file elife-102743-fig2-figsupp3-data1.zip › Figure 2-figure supplement 3_Source Data 1/Figure 2-figure supplement 3-rawimage11.tif]

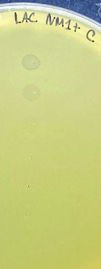

Supplement: Figure 2—figure supplement 3—source data 1. [file elife-102743-fig2-figsupp3-data1.zip › Figure 2-figure supplement 3_Source Data 1/Figure 2-figure supplement 3-rawimage2.tif]

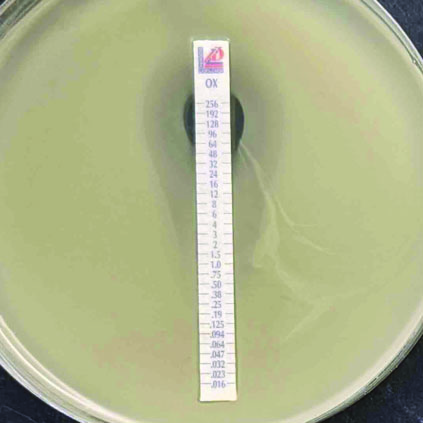

Supplement: Figure 2—figure supplement 3—source data 1. [file elife-102743-fig2-figsupp3-data1.zip › Figure 2-figure supplement 3_Source Data 1/Figure 2-figure supplement 3-rawimage3.tif]

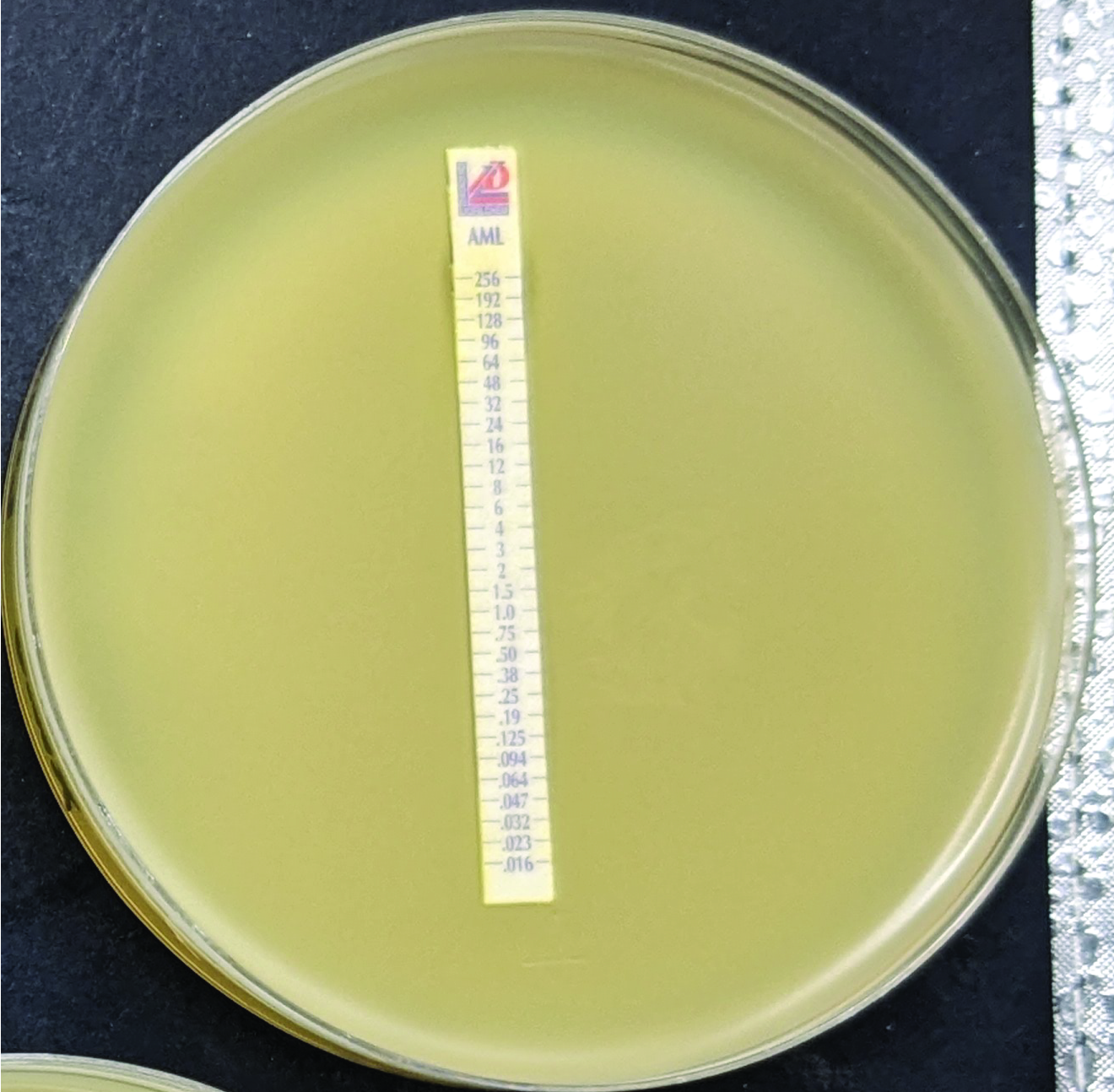

Supplement: Figure 2—figure supplement 3—source data 1. [file elife-102743-fig2-figsupp3-data1.zip › Figure 2-figure supplement 3_Source Data 1/Figure 2-figure supplement 3-rawimage10.tif]

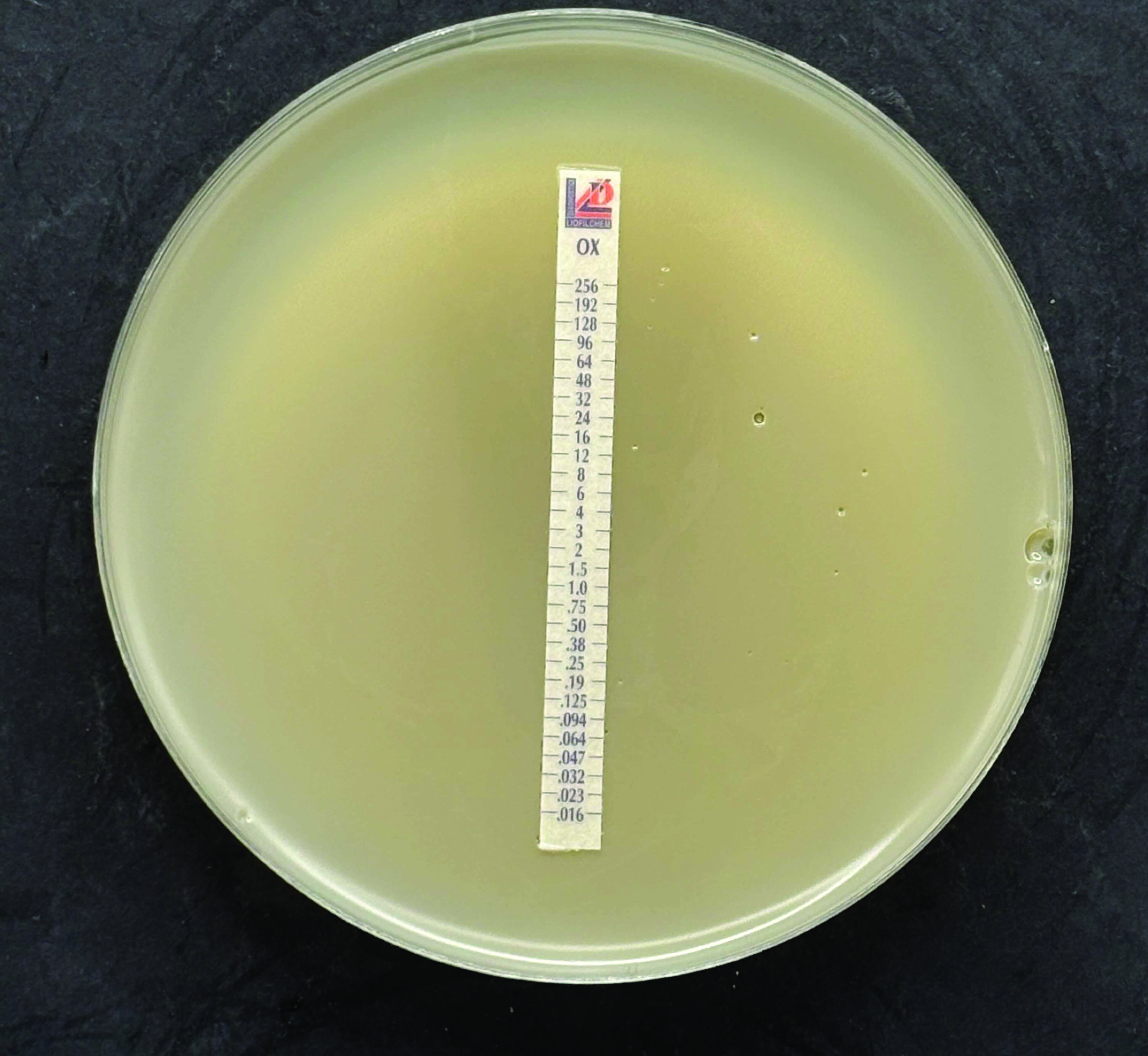

Supplement: Figure 2—figure supplement 4—source data 1. [file elife-102743-fig2-figsupp4-data1.zip › Figure2-figure supplement 4_Source Data 1/Figure2-figure supplement 4_rawimage7 copy.jpg]

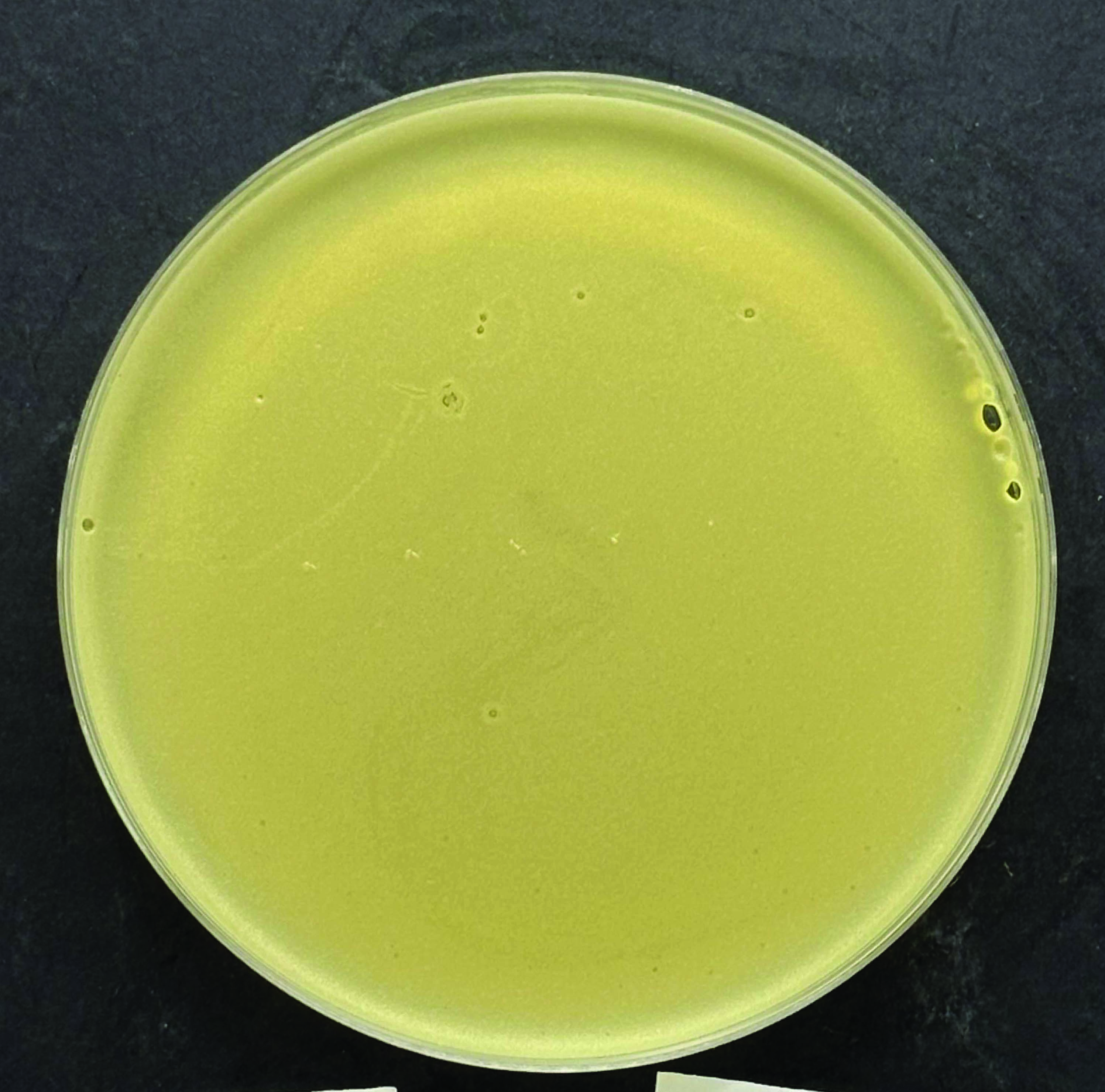

Supplement: Figure 2—figure supplement 4—source data 1. [file elife-102743-fig2-figsupp4-data1.zip › Figure2-figure supplement 4_Source Data 1/Figure2-figure supplement 4_rawimage6 copy.jpg]

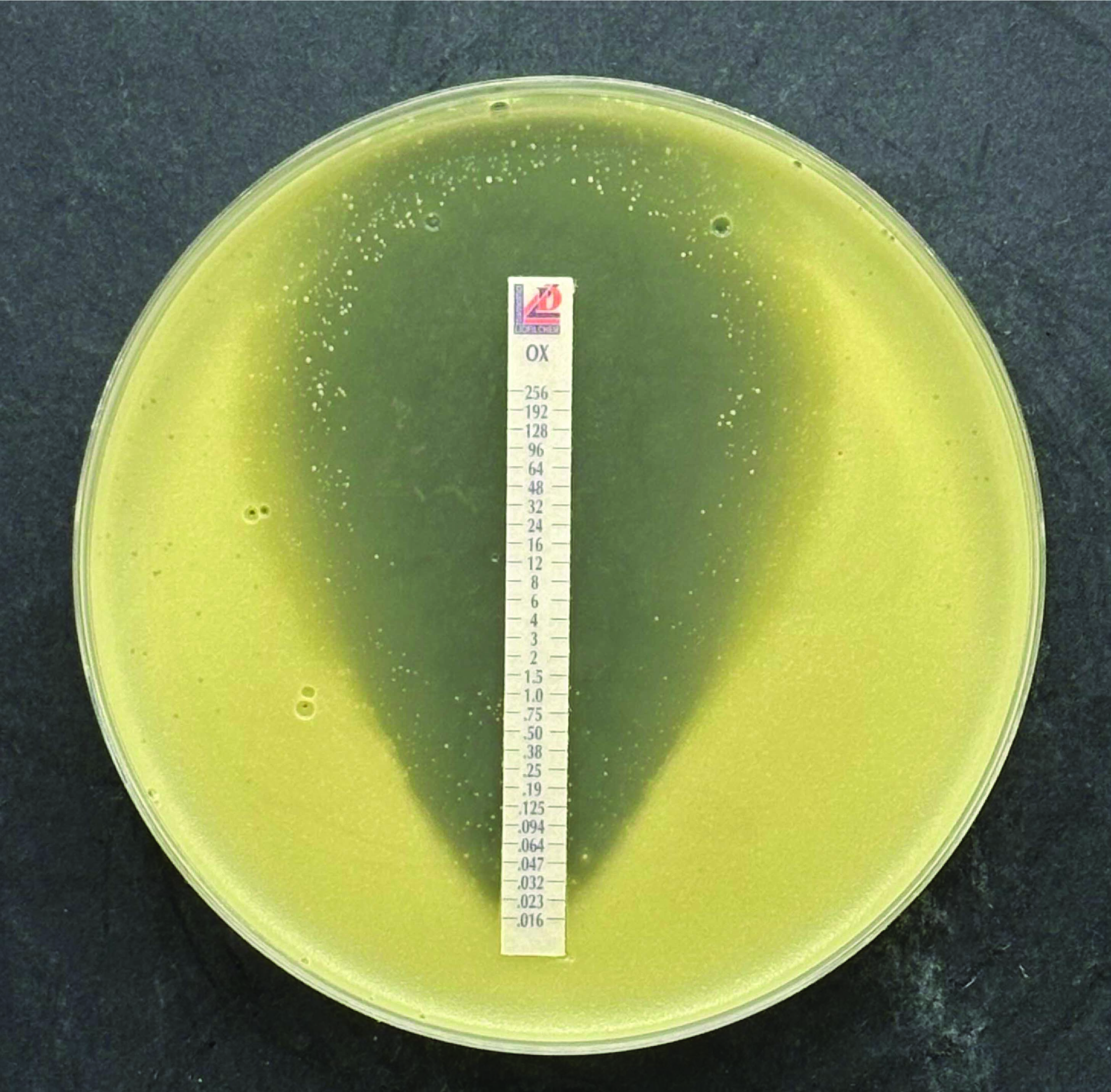

Supplement: Figure 2—figure supplement 4—source data 1. [file elife-102743-fig2-figsupp4-data1.zip › Figure2-figure supplement 4_Source Data 1/Figure2-figure supplement 4_rawimage12 copy.jpg]

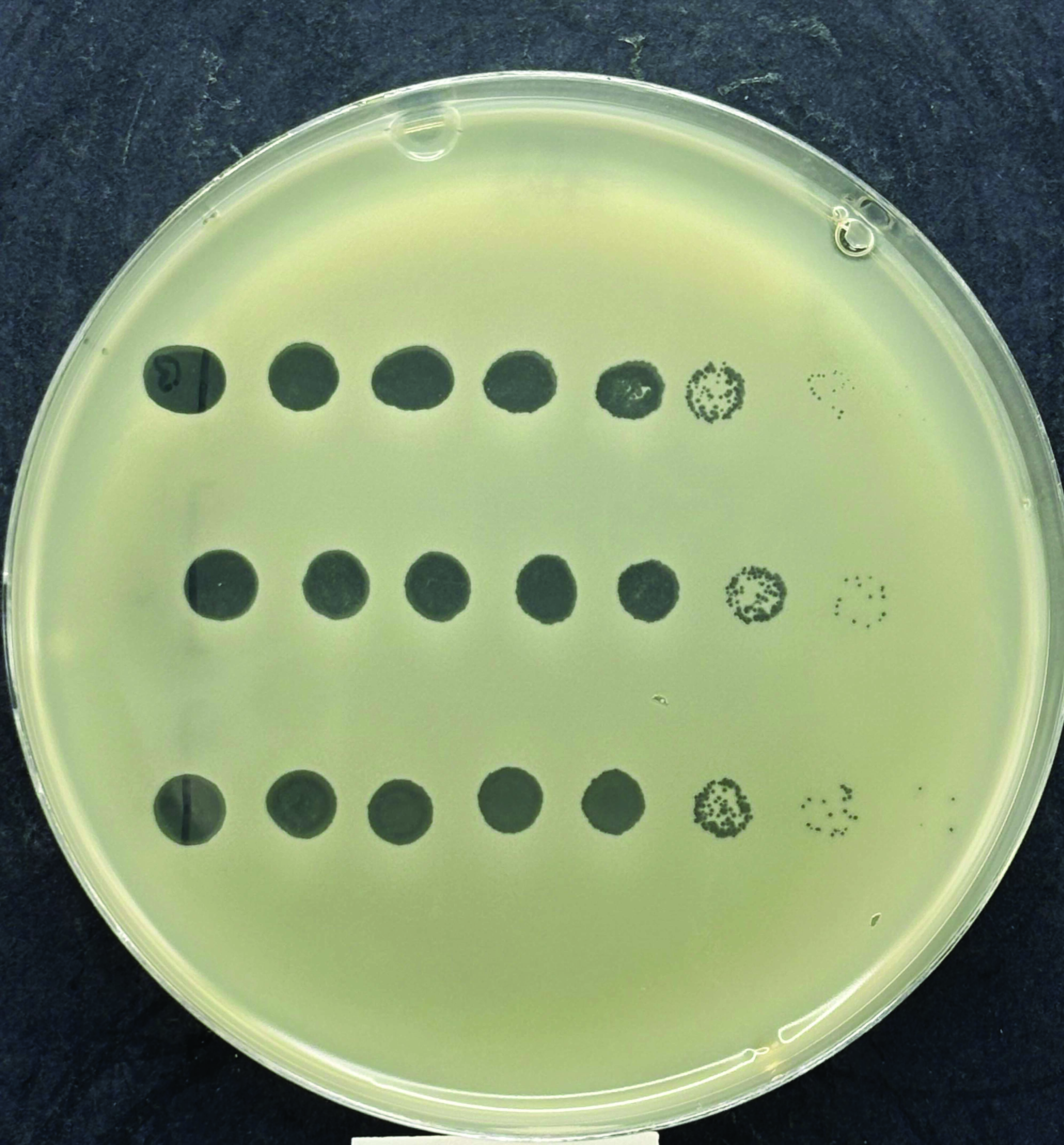

Supplement: Figure 2—figure supplement 4—source data 1. [file elife-102743-fig2-figsupp4-data1.zip › Figure2-figure supplement 4_Source Data 1/Figure2-figure supplement 4_rawimage1 copy.jpg]

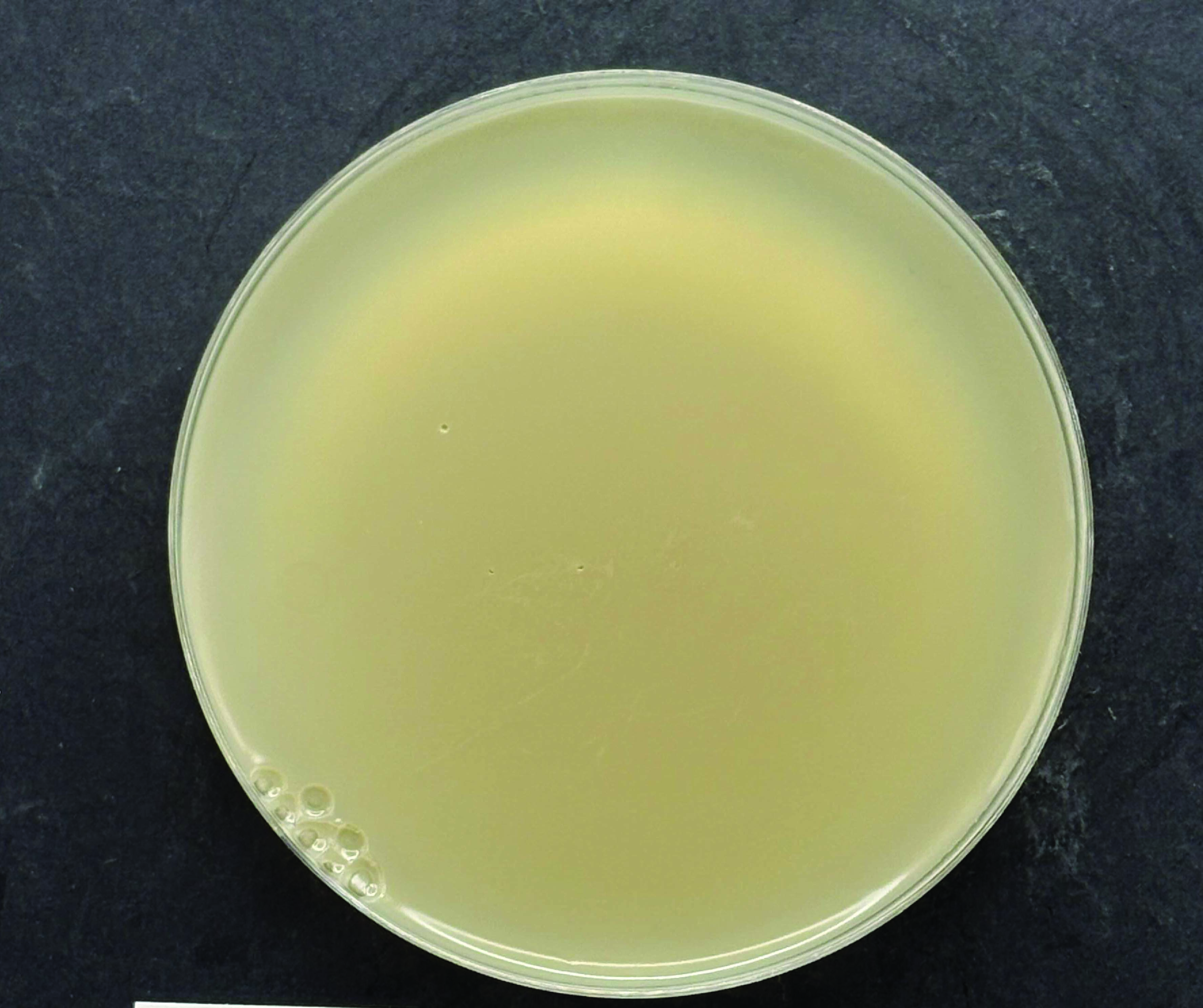

Supplement: Figure 2—figure supplement 4—source data 1. [file elife-102743-fig2-figsupp4-data1.zip › Figure2-figure supplement 4_Source Data 1/Figure2-figure supplement 4_rawimage4 copy.jpg]

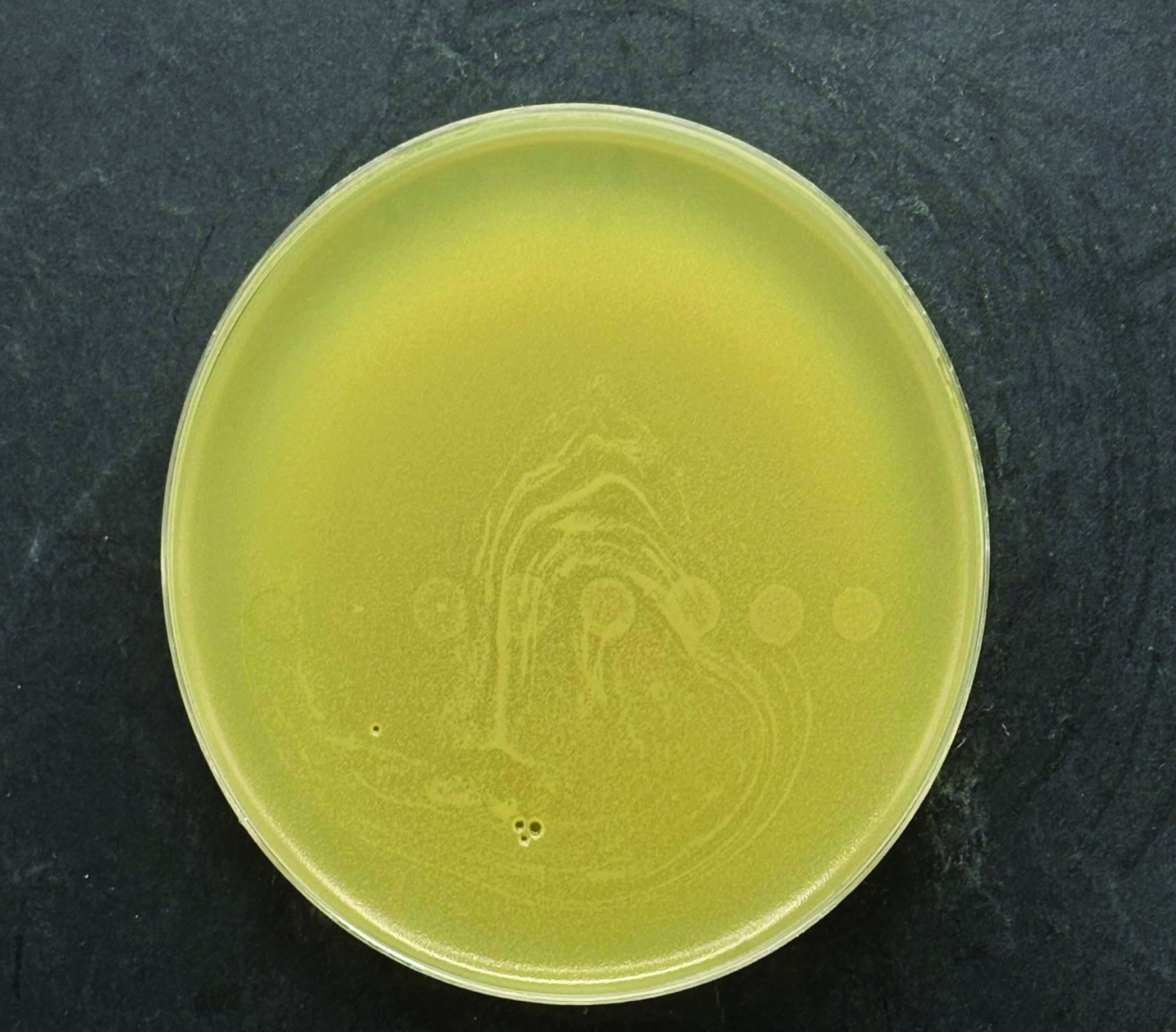

Supplement: Figure 2—figure supplement 4—source data 1. [file elife-102743-fig2-figsupp4-data1.zip › Figure2-figure supplement 4_Source Data 1/Figure2-figure supplement 4_rawimage5 copy.jpg]

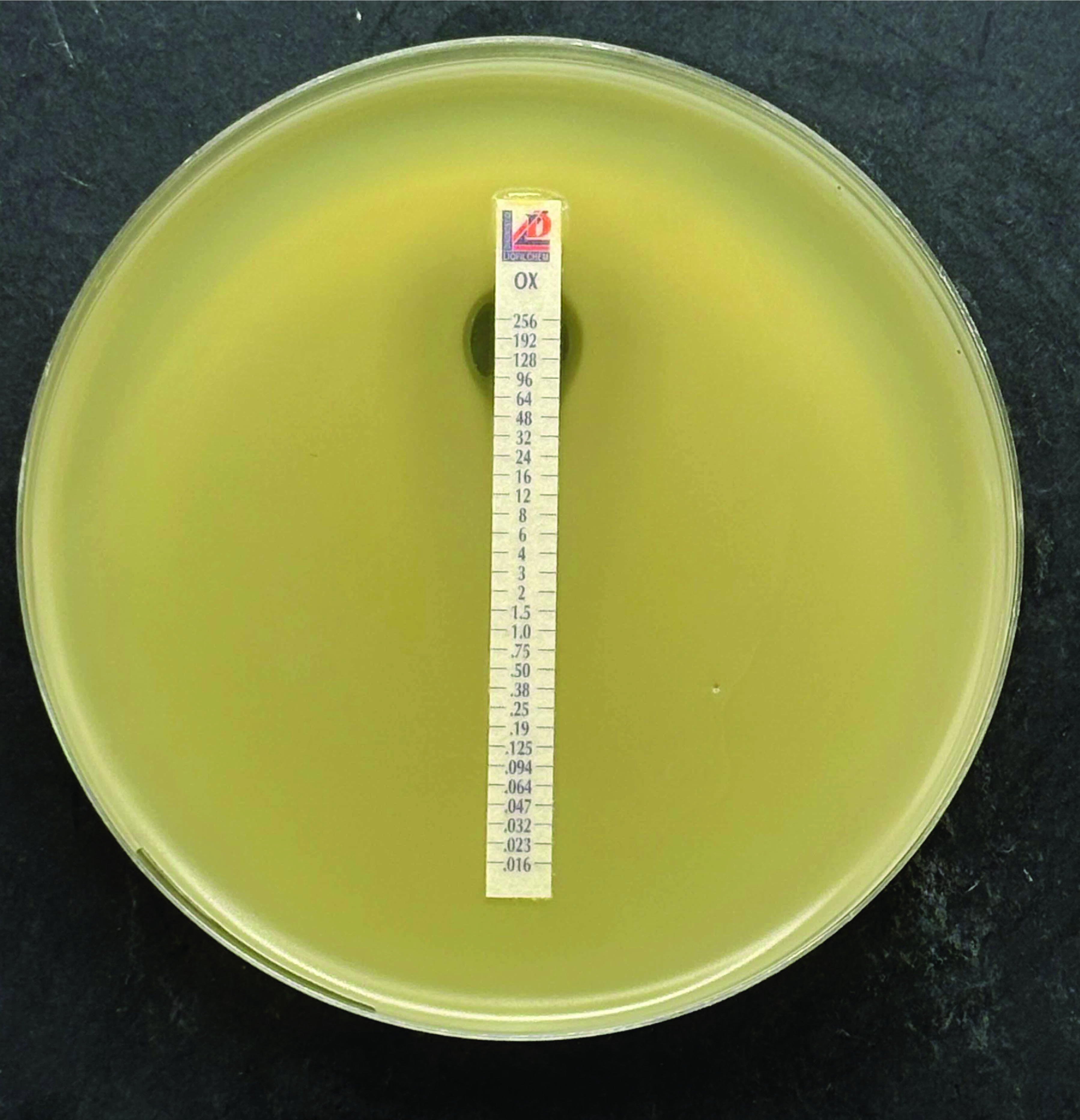

Supplement: Figure 2—figure supplement 4—source data 1. [file elife-102743-fig2-figsupp4-data1.zip › Figure2-figure supplement 4_Source Data 1/Figure2-figure supplement 4_rawimage9 copy.jpg]

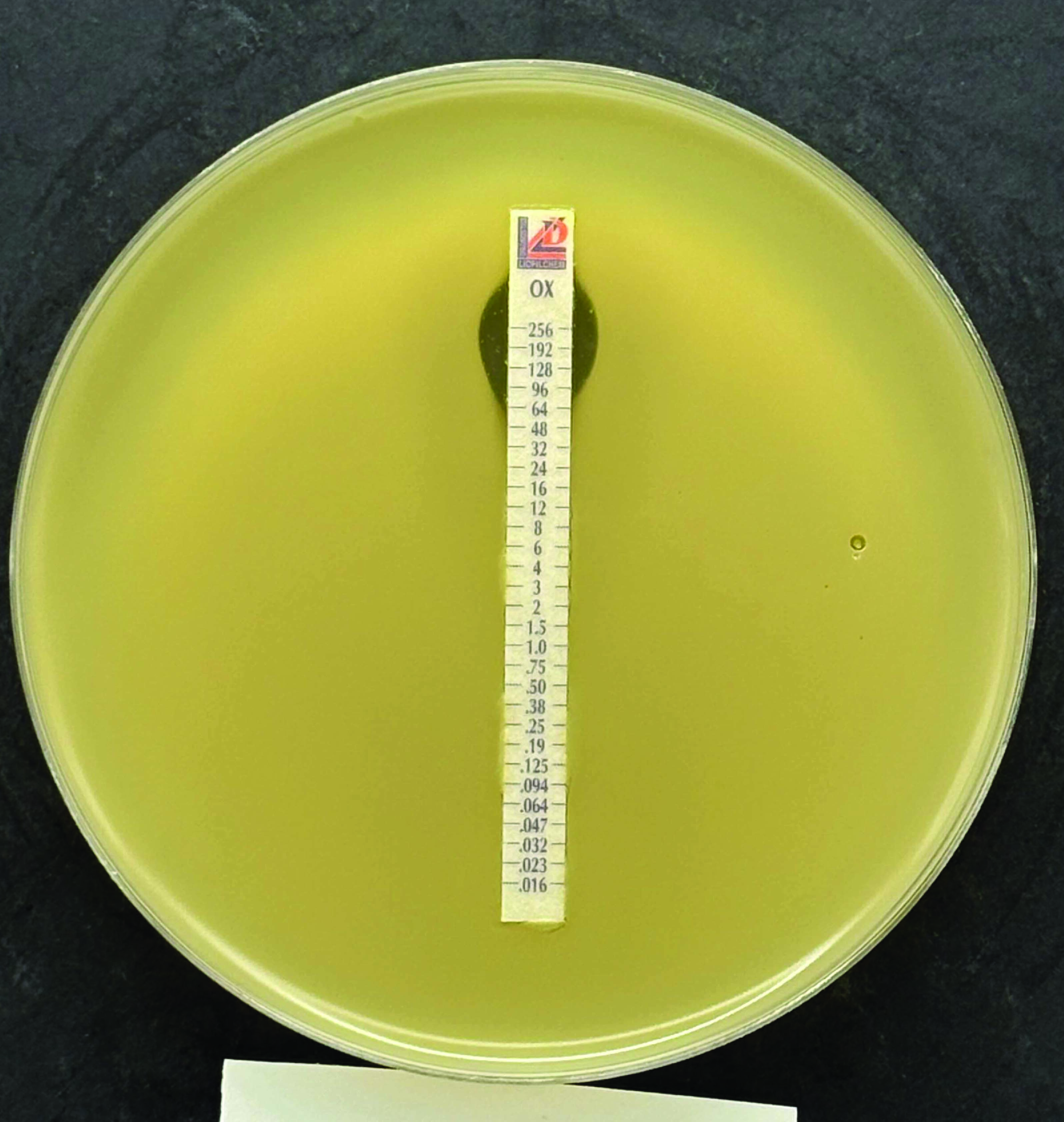

Supplement: Figure 2—figure supplement 4—source data 1. [file elife-102743-fig2-figsupp4-data1.zip › Figure2-figure supplement 4_Source Data 1/Figure2-figure supplement 4_rawimage8 copy.jpg]

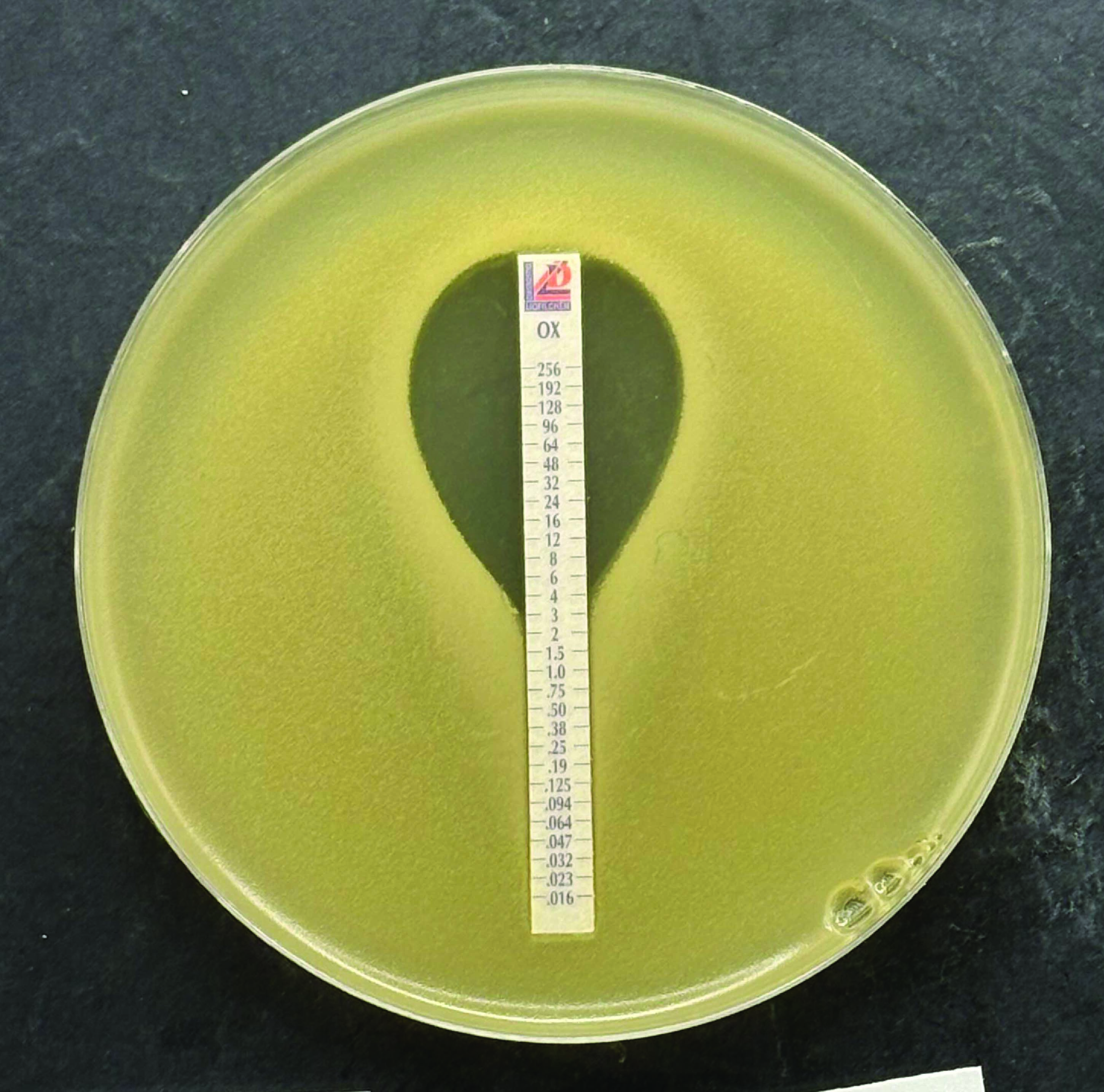

Supplement: Figure 2—figure supplement 4—source data 1. [file elife-102743-fig2-figsupp4-data1.zip › Figure2-figure supplement 4_Source Data 1/Figure2-figure supplement 4_rawimage11 copy.jpg]

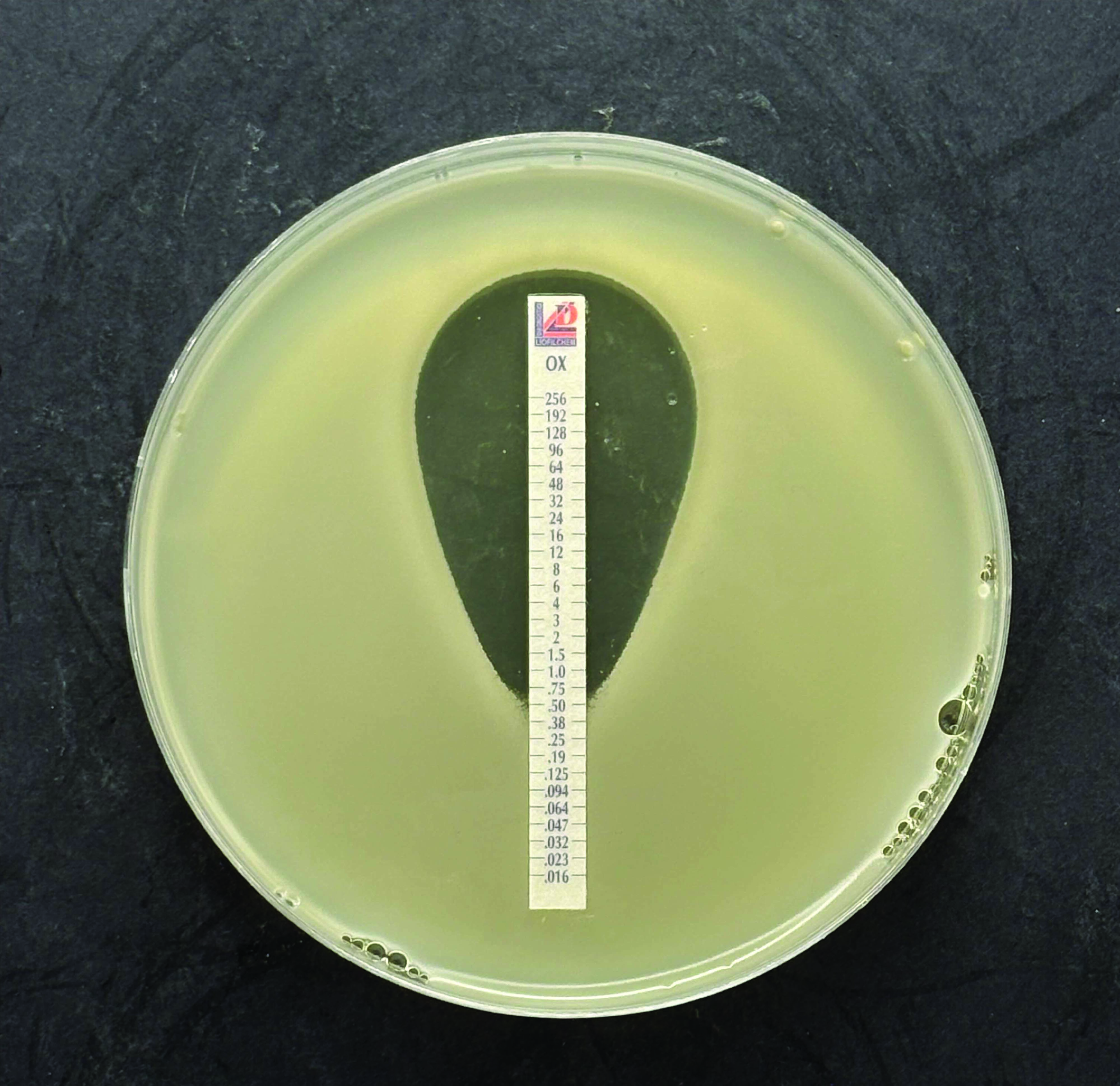

Supplement: Figure 2—figure supplement 4—source data 1. [file elife-102743-fig2-figsupp4-data1.zip › Figure2-figure supplement 4_Source Data 1/Figure2-figure supplement 4_rawimage10 copy.jpg]

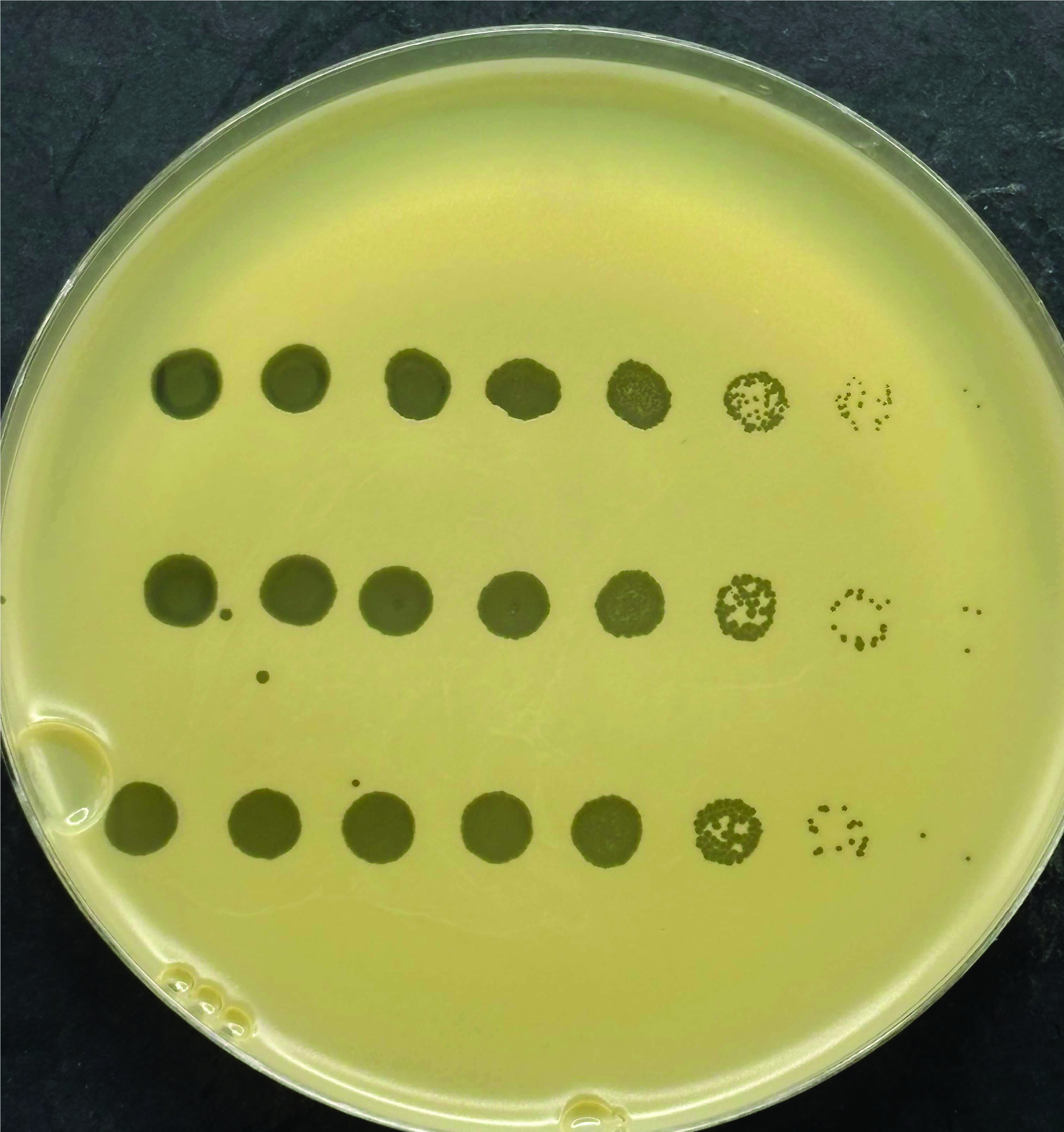

Supplement: Figure 2—figure supplement 4—source data 1. [file elife-102743-fig2-figsupp4-data1.zip › Figure2-figure supplement 4_Source Data 1/Figure2-figure supplement 4_rawimage3 copy.jpg]

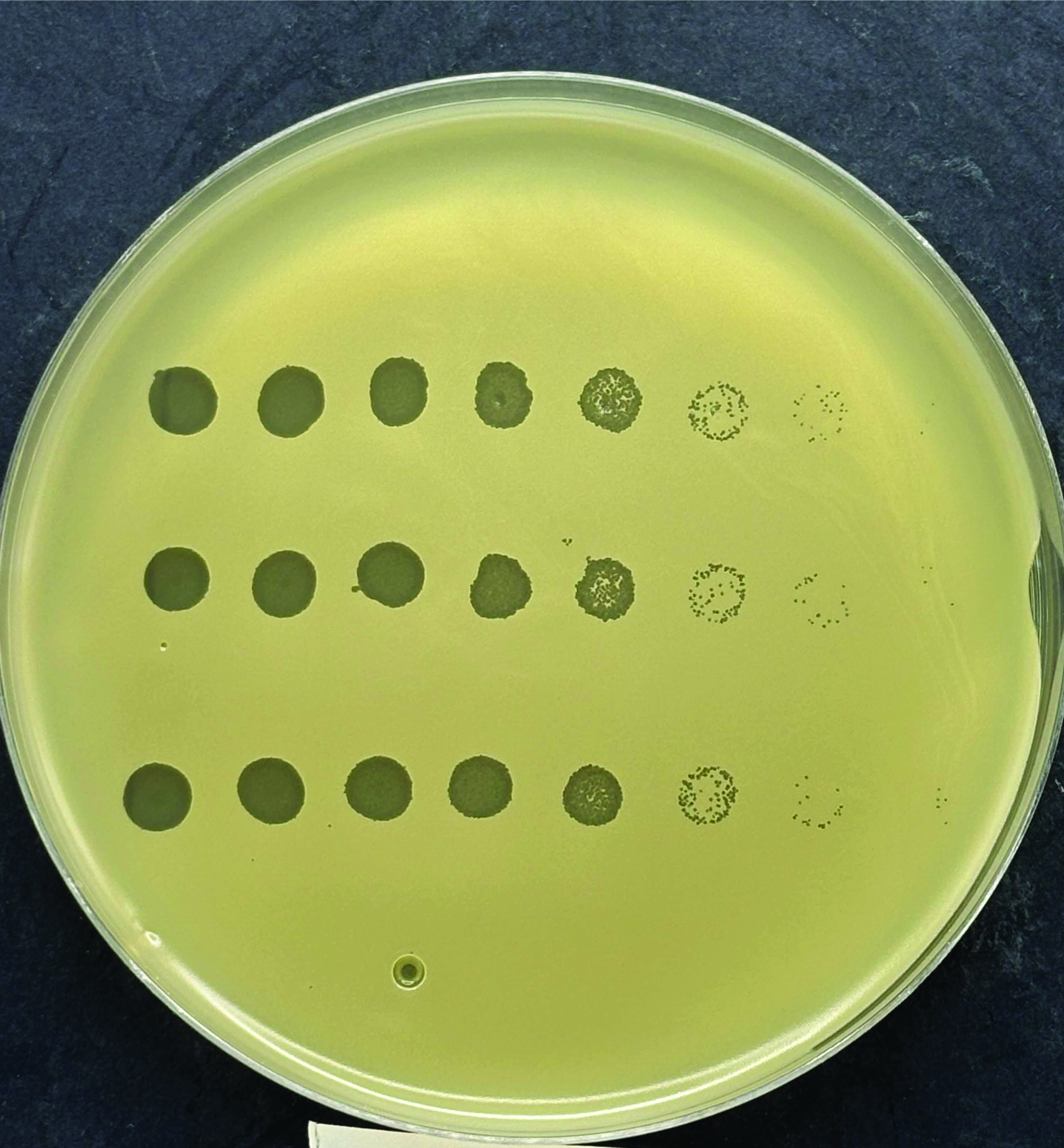

Supplement: Figure 2—figure supplement 4—source data 1. [file elife-102743-fig2-figsupp4-data1.zip › Figure2-figure supplement 4_Source Data 1/Figure2-figure supplement 4_rawimage2 copy.jpg]

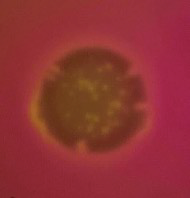

Supplement: Figure 5—source data 1. [file elife-102743-fig5-data1.zip › Figure5-Source Data 1/Figure5-rawimage1.tif]

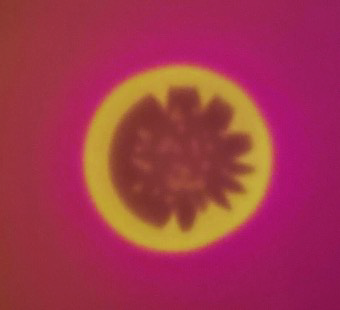

Supplement: Figure 5—source data 1. [file elife-102743-fig5-data1.zip › Figure5-Source Data 1/Figure5-rawimage3.tif]

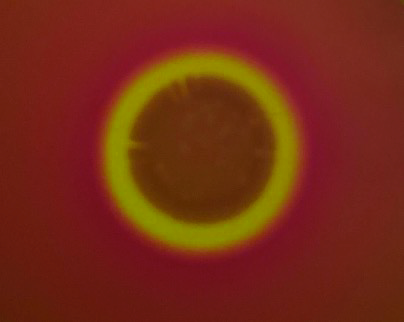

Supplement: Figure 5—source data 1. [file elife-102743-fig5-data1.zip › Figure5-Source Data 1/Figure5-rawimage2.tif]
