# Supplementary material for: Bacteriophage infection drives loss of β-lactam resistance in methicillin-resistant Staphylococcus aureus
Source: eLife. 2025 Jul 10;13:RP102743. doi: 10.7554/eLife.102743 (PMC12245174; doi:10.7554/eLife.102743)

Figure 1-figure supplement 1-Source Data 1: raw plaquing images of bacteriophages against MRSA strains MRSA252, MW2, and LAC. The top raw image is also found in Figure 1-Source Data 1 and Figure 2-Source Data 1

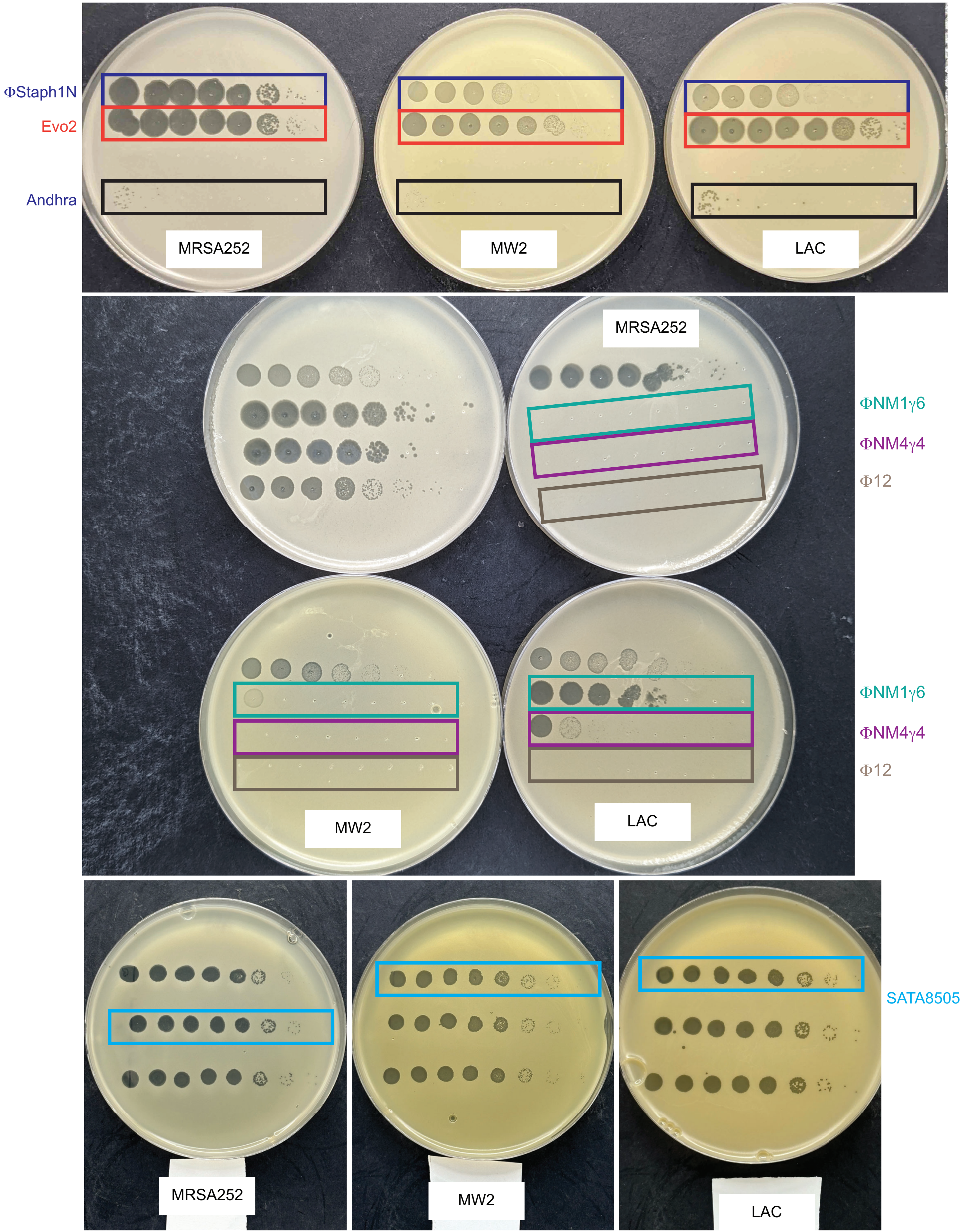

Supplement: Figure 1—figure supplement 1—source data 1. [file elife-102743-fig1-figsupp1-data1.zip › Figure1-figure supplement1_Source Data 1/Figure1_figure supplement 1_Source Data 1.pdf]
