# Supplementary material for: Bacteriophage infection drives loss of β-lactam resistance in methicillin-resistant Staphylococcus aureus
Source: eLife. 2025 Jul 10;13:RP102743. doi: 10.7554/eLife.102743 (PMC12245174; doi:10.7554/eLife.102743)

Figure 2- Source Data 1

For Figure 2A: raw plaquing images of ΦStaph1N and Evo2 bacteriophages against MRSA strains MRSA252, MW2, and LAC. The raw image is also found in Figure 1-Source Data 1 and Figure 1-figure supplement 1-Source Data 1.

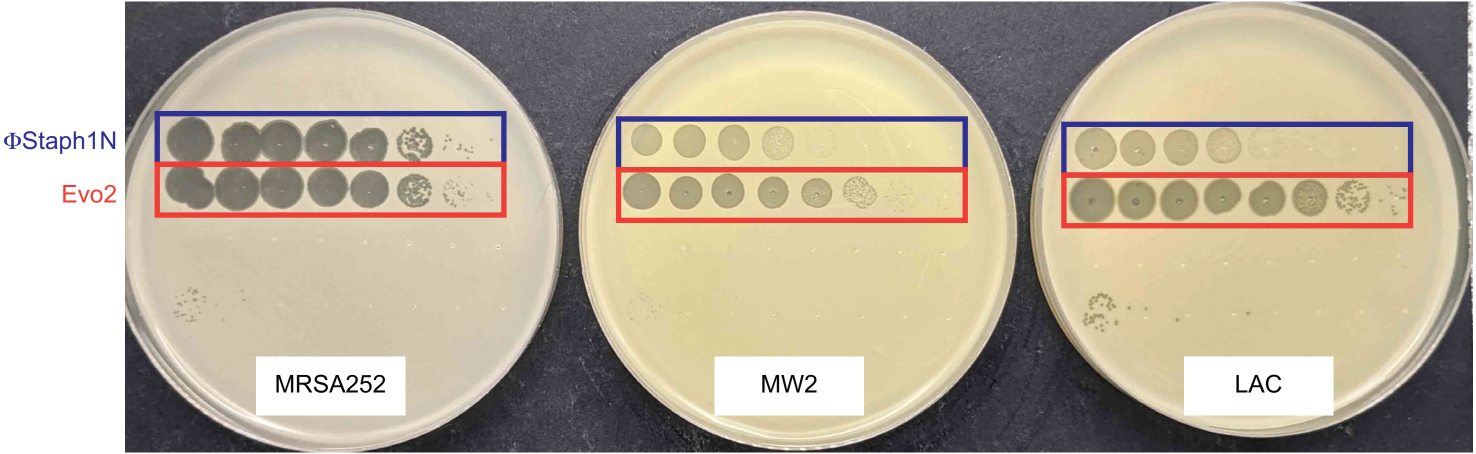

Supplement: Figure 2—source data 1. [file elife-102743-fig2-data1.zip › Figure 2_Source Data 1/Figure 2_Source Data 1.pdf]
