# Supplementary material for: Bacteriophage infection drives loss of β-lactam resistance in methicillin-resistant Staphylococcus aureus
Source: eLife. 2025 Jul 10;13:RP102743. doi: 10.7554/eLife.102743 (PMC12245174; doi:10.7554/eLife.102743)
Supplement: Figure 2—figure supplement 1—source data 1. [file elife-102743-fig2-figsupp1-data1.zip › Figure 2-figure supplement 1_Source Data 1/Figure2_figure supplement 1_Source Data 1.pdf]

Figure 2–figure supplement 1 - Source Data 1

For Figure 2 - figure supplement 1A: Plaquing image of bacteriophage ΦStaph1N against LAC.

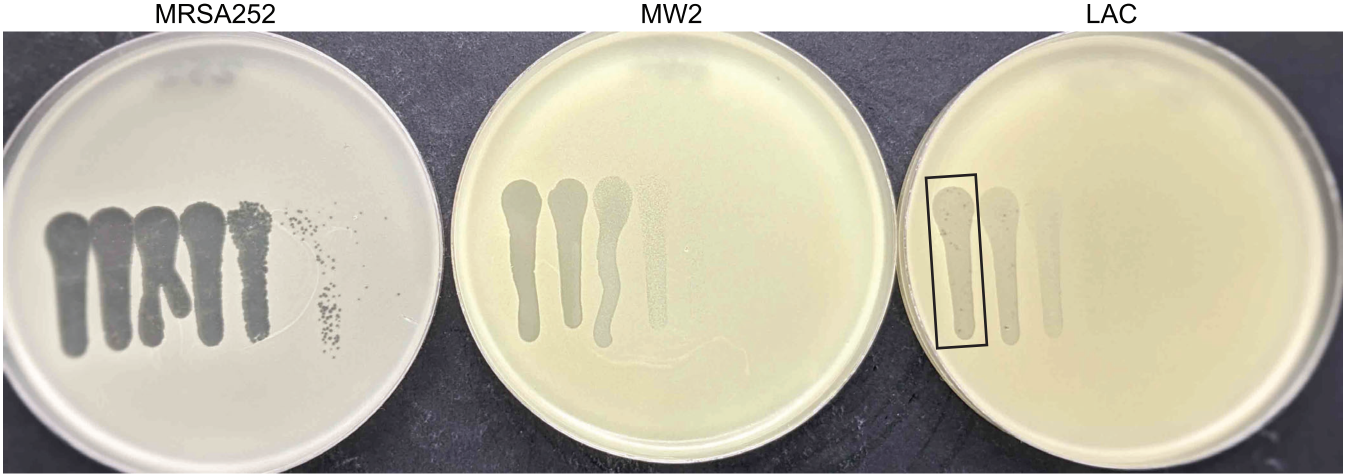

For Figure 2 - figure supplement 1A: the remaining plaquing images are the same as in Figure 2A and share the same Figure 2 - Source Data 1.
