# Supplementary material for: Bacteriophage infection drives loss of β-lactam resistance in methicillin-resistant Staphylococcus aureus
Source: eLife. 2025 Jul 10;13:RP102743. doi: 10.7554/eLife.102743 (PMC12245174; doi:10.7554/eLife.102743)

Figure 2–figure supplement 3 - Source Data 1

For Figure 2 - figure supplement 3A: Plaquing image of bacteriophage ΦNM1γ6 against parental LAC and LAC following ΦNM1γ6 infection (Evolved).

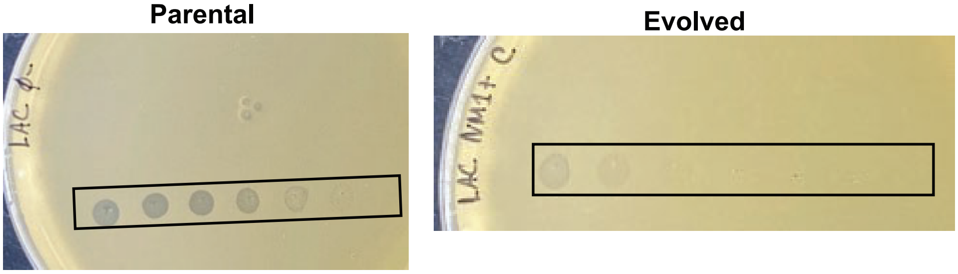

For Figure 2 - figure supplement 3B: images of parental and ΦNM1γ6-resistant LAC strains exposed to oxacillin strips

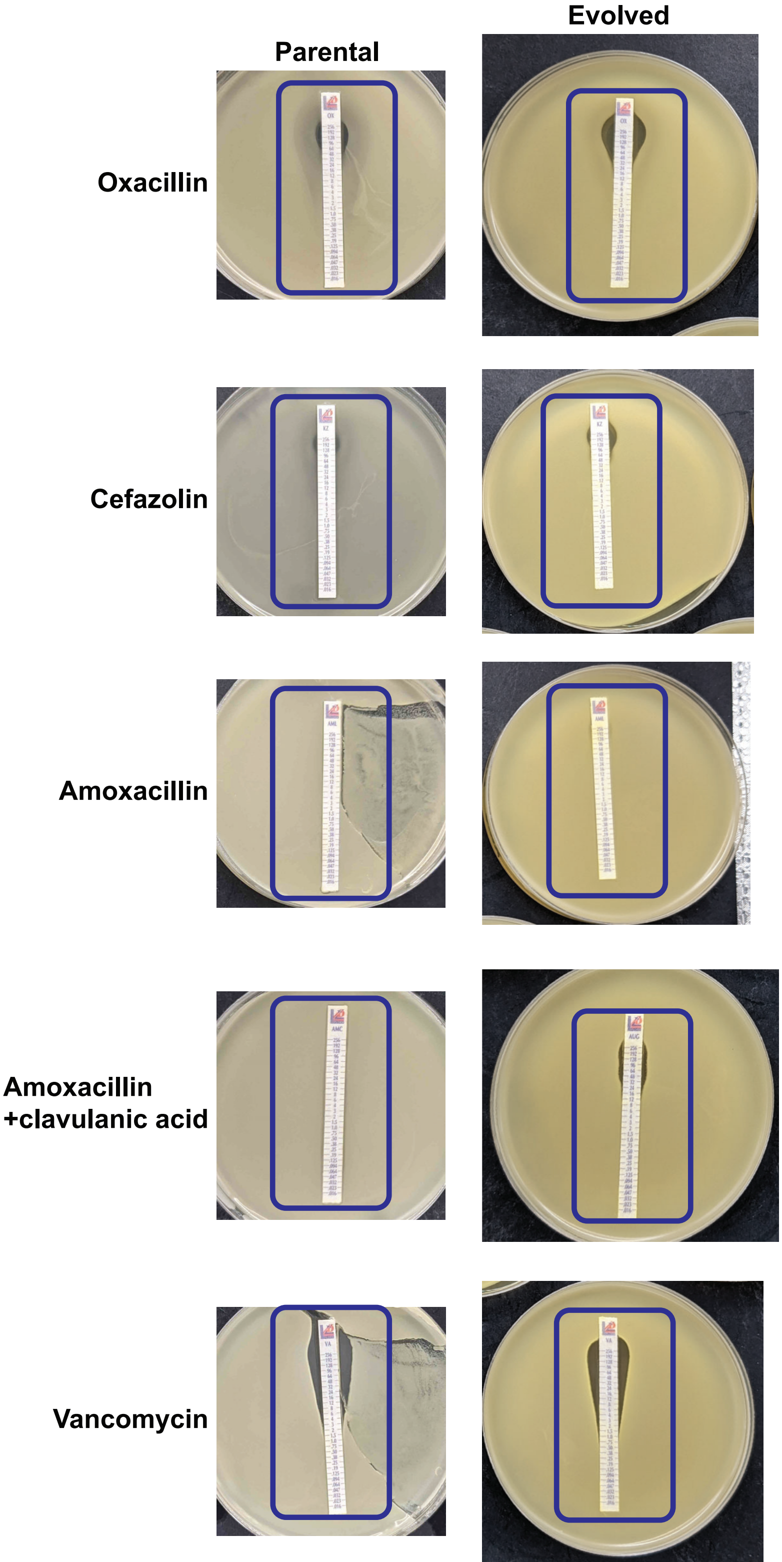

Supplement: Figure 2—figure supplement 3—source data 1. [file elife-102743-fig2-figsupp3-data1.zip › Figure 2-figure supplement 3_Source Data 1/Figure2_figure supplement 3_Source Data 1.pdf]
