# Supplementary material for: Bacteriophage infection drives loss of β-lactam resistance in methicillin-resistant Staphylococcus aureus
Source: eLife. 2025 Jul 10;13:RP102743. doi: 10.7554/eLife.102743 (PMC12245174; doi:10.7554/eLife.102743)

Figure 2-figure supplement 4-Source Data 1:

For Figure 2-figure supplememt 4A: raw plaquing images of bacteriophage SATA8505 against parental and evolved MRSA strains MRSA252, MW2, and LAC.

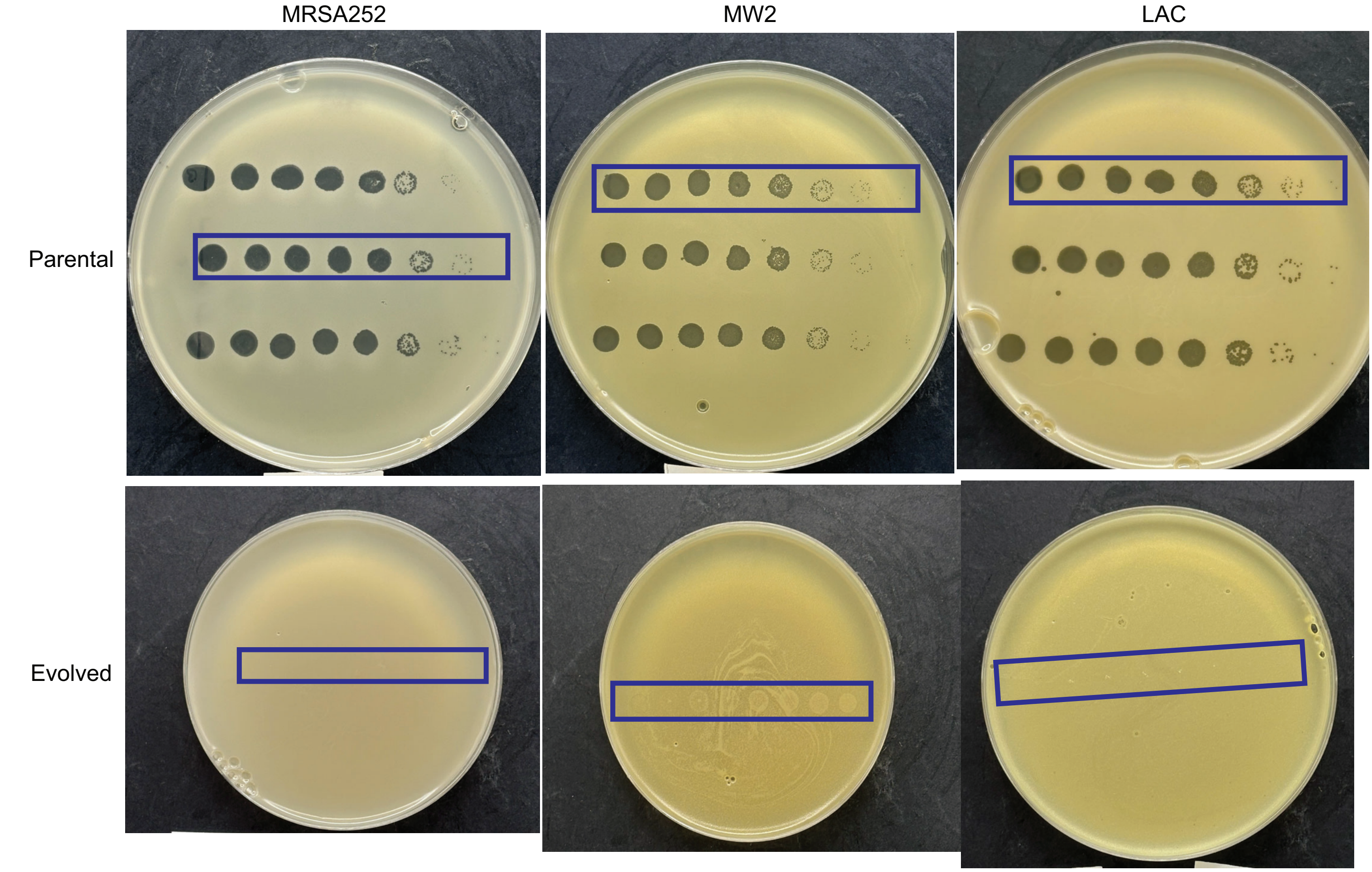

For Figure 2-figure supplememt 4B: Uncropped images of parental and SATA8505-evolved MRSA strains exposed to oxacillin strips.

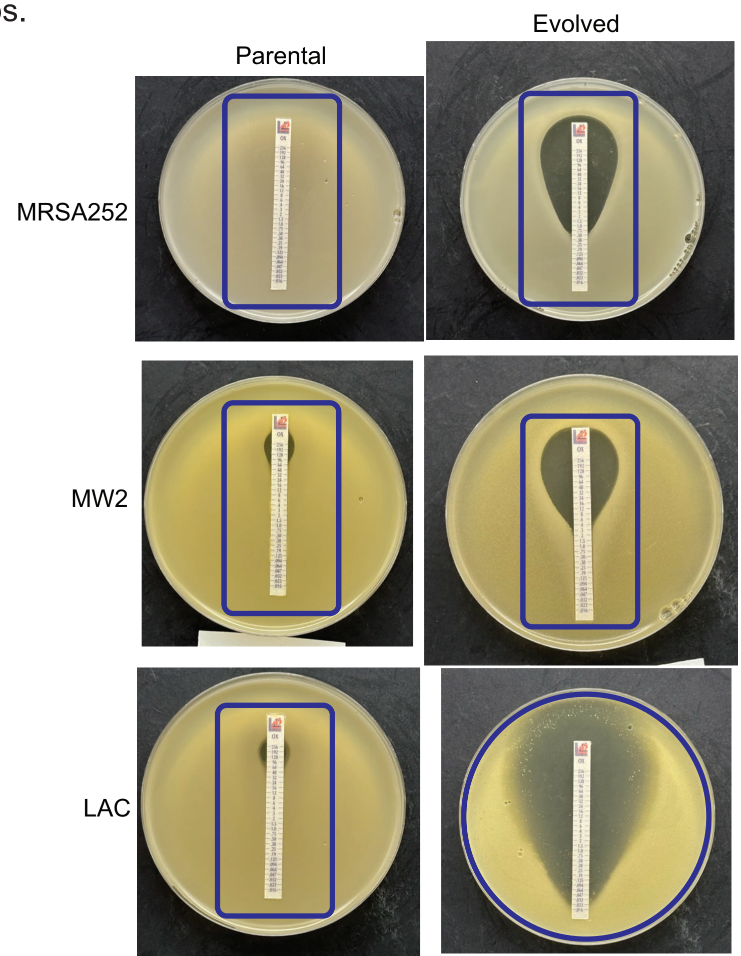

Supplement: Figure 2—figure supplement 4—source data 1. [file elife-102743-fig2-figsupp4-data1.zip › Figure2-figure supplement 4_Source Data 1/Figure2_figure supplement 4_Source Data 1.pdf]
