# Supplementary material for: Bacteriophage infection drives loss of β-lactam resistance in methicillin-resistant Staphylococcus aureus
Source: eLife. 2025 Jul 10;13:RP102743. doi: 10.7554/eLife.102743 (PMC12245174; doi:10.7554/eLife.102743)

For Figure 3-figure supplement 2: Plaquing images of bacteriophage ΦStaph1N and Evo2 against mutant MW2 and LAC strains.

AH3456 (MW2 *mgrA*::tetM)

AH5679 (MW2 *sarA*::Tn(Erm))

AH843 (MW2)

AH3060 (MW2 *arl*::tet)

AH3455 (LAC *mgrA*::tetM)

AH1975 (LAC Δ*arl*)

AH1263 (LAC, erm^{S})

AH1525 (LAC *sarA*::kan)

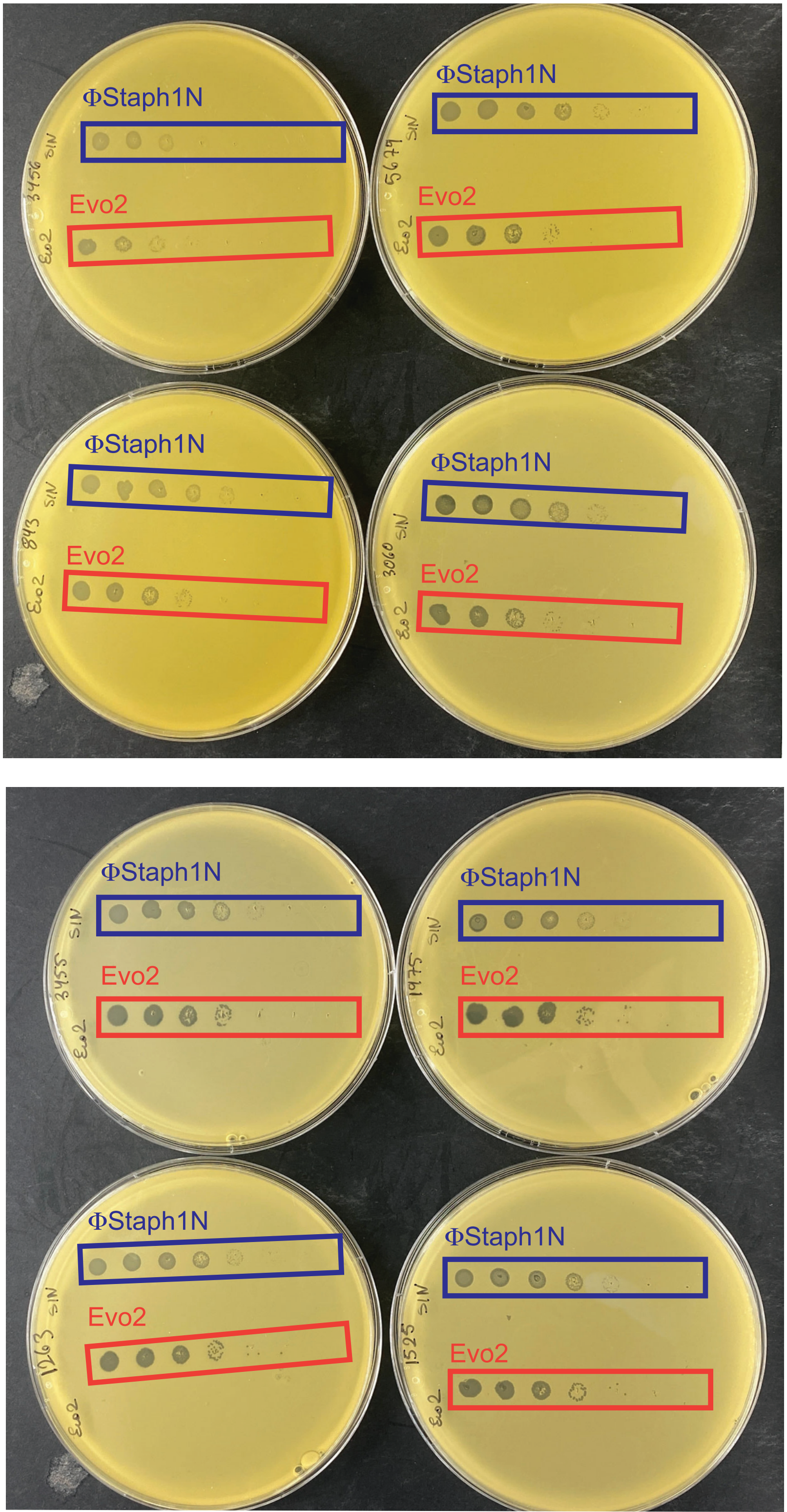

Supplement: Figure 3—figure supplement 2—source data 1. [file elife-102743-fig3-figsupp2-data1.zip › Figure 3-figure supplement 2_Source Data 1/Figure3_figure supplement 2_Source Data 1.pdf]
