# Supplementary material for: Bacteriophage infection drives loss of β-lactam resistance in methicillin-resistant Staphylococcus aureus
Source: eLife. 2025 Jul 10;13:RP102743. doi: 10.7554/eLife.102743 (PMC12245174; doi:10.7554/eLife.102743)

Figure 5 - Source Data 1

For Figure 5A: Uncropped images of MRSA strains MRSA252, MW2, and LAC in blood agar plates.

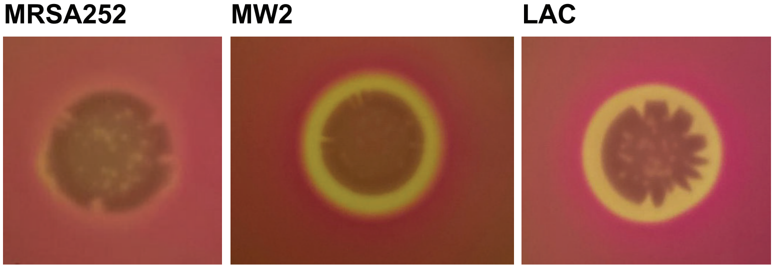

Supplement: Figure 5—source data 1. [file elife-102743-fig5-data1.zip › Figure5-Source Data 1/Figure5-Source Data 1.pdf]
