# Supplementary material for: Bacteriophage infection drives loss of β-lactam resistance in methicillin-resistant Staphylococcus aureus
Source: eLife. 2025 Jul 10;13:RP102743. doi: 10.7554/eLife.102743 (PMC12245174; doi:10.7554/eLife.102743)
Supplement: Supplementary file 3. [file elife-102743-supp3.docx]

**Infección por bacteriófagos provoca la pérdida de resistencia a los β-lactámicos en *Staphylococcus aureus* resistente a la meticilina**

My Tran*, Angel J. Hernandez Viera*, Patricia Q. Tran, Erick Nilsen, Lily Tran, Charlie Y. Mo

Departamento de Bacteriología, Universidad de Wisconsin-Madison, EEUU

*Denota contribución igualitaria

Correspondencia: [cymo@wisc.edu](mailto:cymo@wisc.edu)

**Resumen**

La terapia de bacteriófagos (fagos) es un método prometedor para combatir patógenos bacterianos resistentes a antibióticos. La infección por fagos puede seleccionar mutaciones en poblaciones bacterianas que confieren resistencia a la infección por fagos. Sin embargo, la resistencia a los fagos puede generar compensaciones o contrapartidas evolutivas de relevancia biomédica. En esta publicación, informamos del descubrimiento de que la infección por ciertos fagos estafilocócicos sensibiliza a diferentes cepas de *Staphylococcus aureus* resistente a la meticilina (SARM) a los β-lactámicos, una clase de antibióticos contra los cuales el SARM es resistente. Las bacterias de SARM que sobreviven a la infección por estos fagos muestran reducciones significativas en la concentración inhibitoria mínima (CMI) contra diferentes β-lactámicos en comparación con las bacterias no infectadas. El perfil transcriptómico revela que estas cepas evolucionadas de SARM poseen perfiles transcripcionales altamente modulados, donde numerosos genes involucrados en la virulencia de *S. aureus* fueron subexpresados. El SARM tratado con fagos mostró fenotipos de virulencia atenuados, en forma de reducción de hemólisis y agregación mediada por fibronectina. A pesar de compartir fenotipos similares, el análisis de secuenciación del genoma reveló que las diferentes cepas de SARM desarrollaron perfiles genéticos únicos durante la infección. Estos resultados sugieren trayectorias evolutivas complejas en el SARM durante la depredación por fagos y abren nuevas posibilidades para reducir la resistencia a antibióticos y virulencia en las infecciones por SARM.

**Declaración de Impacto**

La evolución de la resistencia a bacteriófagos resensibiliza a *Staphylococcus aureus* resistente a la meticilina a los antibióticos β-lactámicos.

**Introducción**

*Staphylococcus aureus* es uno de los patógenos bacterianos más comunes y conocidos, es responsable de cientos de miles de infecciones graves en todo el mundo cada año.

*S. aureus* resistente a la meticilina (SARM) representa una amenaza clínica particular, ya que las infecciones por SARM aumentan la mortalidad, la morbilidad y la estancia hospitalaria, en comparación con las causadas por *S. aureus* sensible a la meticilina.¹ Parte de la notoriedad del SARM se debe a su fuerte resistencia a los antibióticos de la familia β-lactámicos, como las penicilinas y las cefalosporinas, que inhiben la actividad de las enzimas transpeptidasas durante la síntesis de peptidoglicano en la pared celular bacteriana. Los β-lactámicos forman parte de una de las clases de fármacos más comúnmente recetadas, y muchas de ellas han sido designadas como antimicrobianos de "importancia crítica" por la Organización Mundial de la Salud.^4^ Por lo tanto, las infecciones por SARM representan un riesgo considerable para la salud pública, ya que son notoriamente difíciles de tratar y son comunes en comunidades y entornos hospitalarios.

De hecho, en 2019, el SARM causó más de 100,000 muertes atribuibles a infecciones resistentes a antibióticos en todo el mundo.^4^

Un mediador principal de la resistencia a los β-lactámicos en el SARM son los genes *SCCmec*, un elemento genético móvil que porta el gen de resistencia *mecA*. *mecA* codifica la proteína fijadora de penicilina 2a (PBP2a), una transpeptidasa con baja afinidad por los betalactámicos.^5^ Esta menor afinidad permite que PBP2a participe en la síntesis de peptidoglicano incluso en presencia de β-lactámicos, lo que favorece la supervivencia celular. Además de *mecA*, numerosas cepas de SARM también portan betalactamasas, como BlaZ, que degradan ciertos β-lactámicos, lo que contribuye aún más a la resistencia a estos antibióticos. En conjunto, estos mecanismos pueden limitar gravemente las opciones de tratamiento contra el SARM, las opciones de tratamiento clínico actuales se basan principalmente en la vancomicina y la daptomicina.^6^ Tanto la vancomicina como la daptomicina son antibióticos de último recurso contra el SARM, y una preocupación importante es la creciente resistencia del SARM a estos antibioticos.^7,8^ El desarrollo de soluciones para combatir el SARM es un objetivo principal en el ámbito académico e industrial.

Debido a su resistencia a antibióticos y su impacto clínico, *S. aureus* es un candidato ideal para tratamientos antimicrobianos alternativos, como la terapia de fagos. Los fagos son virus que infectan y destruyen bacterias, lo que representa una de las mayores amenazas para las comunidades bacterianas. Algunos estimados sugieren que el 40 % de la mortalidad bacteriana mundial se debe a la depredación de fagos.^9^ En la terapia de fagos, se administran fagos líticos para destruir el o los patógenos bacterianos causantes de una infección. Los fagos ofrecen ciertas ventajas sobre los antibióticos tradicionales: son altamente específicos a sus huéspedes, reduciendo la destrucción de microorganismos fuera del patógeno; se autoamplifican y evolucionan, lo que permite la rápida generación de nuevas variantes de fagos con mejor actividad; y generalmente se consideran seguros, ya que solo se ha informado de toxicidad en casos extremadamente raros en animales y pacientes.^10^ De hecho, en la última década se han publicado más de una docena de estudios de caso y ensayos clínicos prometedores contra las infecciones por *S. aureus*.^11^

A pesar de estos avances, el uso rutinario de la terapia de fagos aún enfrenta desafíos. El principal de estos es el inevitable aumento de la resistencia a los fagos, ya que su depredación ejerce una fuerte presión selectiva sobre las poblaciones bacterianas. Según un metaanálisis centrado en los resultados en el uso de terapia de fagos, la resistencia a los fagos evolucionó en el 75 % de los casos clínicos humanos en los que se monitoreó la evolución de la resistencia.^12^ Las mutaciones representan una vía principal por la cual las bacterias desarrollan resistencia a los fagos. Hasta la fecha, las mutaciones de resistencia a fagos mejor caracterizadas implican alteraciones en las moléculas del receptor en la superficie celular que median la adhesión de fagos. En muchas bacterias, estos receptores suelen ser proteínas o glúcidos, que son reconocidos por las proteínas de los fagos.^11,13,14^ Por ejemplo, en *Escherichia coli*, las mutaciones en la proteína de la pared celular LamB confieren resistencia contra la infección por el fago lambda, mientras que en *S. aureus*, se ha demostrado que las mutaciones que modifican los ácidos teicoicos asociados a la pared celular (WTA, por sus siglas en inglés) limitan la infección por fagos.^14,15^ Para complicar el panorama, otros estudios han revelado que otros mecanismos adicionales del huésped bacteriano, incluyendo sistemas de defensa contra fagos , pueden afectar la evolución de la resistencia contra los fagos.^16,17^ Estos problemas resaltan la importancia de desarrollar estrategias de tratamiento con fagos que minimicen o capitalicen la evolución de la resistencia a los fagos.^18^

Un aspecto único de la terapia de fagos es la posibilidad de aprovechar las compensaciones evolutivas para combatir patógenos resistentes. Una compensación evolutiva se define como un rasgo evolutivo que confiere una ventaja de adaptación frente a una presión selectiva particular, a expensas de una menor adaptación frente a una presión no seleccionada. En diferentes especies bacterianas, se ha demostrado que estas compensaciones pueden ocurrir entre la resistencia a los fagos y la resistencia a los antibióticos (Figura 1A). Se predice que los fagos que usan como receptor a un factor de virulencia o mecanismo de resistencia a los antibióticos en la bacteria huésped ejercen una fuerte presión selectiva sobre la bacteria para mutar o inhibir el receptor que el fago usa. Estos cambios conferirían protección contra la infección por fagos, pero a su vez podrían reducir la resistencia o la virulencia en la bacteria. Por ejemplo, en *P. aeruginosa*, la infección por el fago OMKO1, que se enlaza a la proteína de membrana externa M OprM de las bombas de eflujo MexAB y MexXY-OprM, impulsa la evolución de mutaciones en dichos genes, lo que lleva a la resensibilización a antibioticos en *P. aeruginosa* resistente a los fagos.^10,19^

Se sabe poco sobre cómo la resistencia a los fagos puede causar compensaciones evolutivas en el SARM. Trabajos previos han demostrado que la resistencia a los fagos en *S. aureus* puede proceder a través de mutaciones genéticas que no están directamente involucradas en la adhesión del fago. Por ejemplo, estudios de Berryhill y colegas demostraron que la infección por fagos en *S. aureus* Newman, una cepa sensible a betalactámicos, puede seleccionar mutaciones en *femA*, una enzima citoplasmática que cataliza la formación de los puentes de pentaglicina en la pared celular de *S. aureus*.^20^ En lugar de servir directamente como un receptor del fago, *femA* mantiene la integridad de la pared celular, que a su vez podría ser vital para la maduración de WTA y la adhesión del fago.^13,21^ Curiosamente, una consecuencia de estas mutaciones de femA es una mayor sensibilidad a los antibióticos betalactámicos. Por lo tanto, nos preguntamos cómo la resistencia a los fagos podría afectar la fisiología del SARM, con la intención de identificar compensaciones evolutivas de posible relevancia biomédica.

En esta publicación, demostramos que la infección por fagos estafilocócicos provoca que diferentes cepas de SARM desarrollen sensibilidad a diferentes antibióticos β-lactámicos y atenúen múltiples fenotipos de virulencia. Descubrimos que esta pérdida de resistencia y virulencia se asocia con perfiles mutacionales distintivos en cada cepa de SARM, y que las poblaciones de SARM evolucionadas tratadas con fagos presentan una remodelación significativa de sus transcriptomas. Sorprendentemente, también descubrimos un fago mutante con mayor actividad y un espectro de hospedadores más amplio contra el SARM. Los hallazgos de nuestro trabajo pueden contribuir al desarrollo de terapias con fagos que reduzcan la farmacorresistencia y la virulencia en bacterias patógenas.

**Resultados**

**Identificación del fago ΦStaph1N con actividad lítica contra múltiples cepas de SARM**

Para nuestros estudios, nos centramos en tres cepas de SARM: MRSA252 (USA200), MW2 (USA400) y LAC (USA300). Estas tres cepas de SARM son aislados clínicos implicados en enfermedades humanas y se utilizan como ejemplos representativos para estudios de SARM.^22–24^ Para evaluar la susceptibilidad a los fagos, realizamos ensayos de placa con un panel de fagos estafilocócicos (Figura 1, Figura 1- Figura suplementaria 1). De los fagos analizados, el fago ΦStaph1N, perteneciente al género Kayvirus, formó placas en las tres cepas de SARM (Figura 1B).^21,25^ Sin embargo, a pesar de su capacidad para infectar las tres cepas, la infección por ΦStaph1N no logró erradicar completamente los cultivos de SARM. Tanto MW2 como LAC mostraron una lisis incompleta en cultivo líquido con multiplicidad de infección (MOI) de 0.1 o inferiores (Figura 1- Figura suplementaria 2). Además, los cultivos infectados de las tres cepas de SARM pudieron recuperar una alta densidad celular tras el pasaje del 1% del cultivo a medio de cultivo fresco tras 24 horas de la infección inicial. Estos resultados sugieren que la infección por ΦStaph1N selecciona a mutantes resistentes causando un barrido selectivo. De hecho, ΦStaph1N no pudo formar placas en cultivos de SARM recuperados que sobrevivieron a la infección inicial con ΦStaph1N (Figura 1B).

**Resistencia a infección de ΦStaph1N sensibiliza al SARM contra los β-lactámicos.**

Dado que tanto los fagos como las β-lactámicos interactúan con la pared celular bacteriana, planteamos la hipótesis de que resistencia a infección de ΦStaph1N podría causar un cambio en la resistencia a los β-lactámicos en el SARM, incluso en presencia de PBP2a y BlaZ. Primero, evaluamos la sensibilidad a los β-lactámicos de las cepas parentales MRSA252, MW2 y LAC. Como era de esperar, las tres cepas mostraron altas concentraciones inhibitorias mínimas (CMI) de ≥48 µg/mL contra los β-lactámicos oxacilina (OXA), cefazolina (CEF), amoxicilina (AMX) y amoxicilina con ácido clavulánico (AMX+CA), lo que se refleja visualmente en su capacidad de formar crecimiento confluente alrededor de tiras de antibióticos (Figura 1C). Las cepas fueron sensibles a la vancomicina (VANC; CMI = 1,5 µg/mL), que inhibe la síntesis de la pared celular mediante un mecanismo diferente al de los β-lactámicos. Sorprendentemente, el SARM resistente a fagos que sobrevivió a la infección por ΦStaph1N mostró una fuerte reducción de la resistencia a OXA, CEF y AMX+CA, con reducciones de la CMI de entre 10 y 1000 veces (Figura 1D); no se observaron cambios en la CMI de VANC ni AMX. Estos resultados muestran, a nivel fenotípico, que el SARM resistente a ΦStaph1N pierde resistencia a los β-lactámicos.

Luego nos preguntamos si esta pérdida de resistencia a β-lactámicos dependía del MOI de ΦStaph1N. Infectamos las tres cepas de SARM con ΦStaph1N con una MOI entre 10^-2^ y 10^-5^, aislamos las células de SARM sobrevivientes y analizamos la CMI de oxacilina (Tabla 1). En el caso de MRSA252, observamos una reducción de la CMI de aproximadamente tres órdenes de magnitud con un MOI de 10^-5^. Con MW2, la reducción de la CMI disminuyó notablemente con niveles más bajos de fagos, sin mostrar una pérdida significativa con MOI de 10^-3^ o inferiores. En el caso de LAC, dos réplicas mostraron una reducción de la CMI de un orden de magnitud con un MOI de 10^-4^, mientras que la tercera réplica no mostró ningún cambio. Estos resultados muestran que, para MRSA252, los MOI de ΦStaph1N tan bajos como 10^-5^ aún pueden provocar la pérdida de resistencia, mientras que, para MW2 y LAC, se necesitan MOI de fago más altos para garantizar el mismo resultado de reducción de la resistencia a los β-lactámicos.

**Tabla 1. Concentracion mínima inhibitoria (µg/mL) de oxacilina contra SARM tratados con diferentes MOI de fagos**

|  | **MRSA252** | | | | | |
| --- | --- | --- | --- | --- | --- | --- |
|  | **ΦStaph1N** | | | **Evo2** | | |
| **MOI** | **Rep 1** | **Rep 2** | **Rep 3** | **Rep 1** | **Rep 2** | **Rep 3** |
| 10^-2^ | 0.25 | 0.125 | 0.38 | NG | 2 | 0.5 |
| 10^-3^ | NG | 0.94 | 0.19 | 1 | 0.75 | 1 |
| 10^-4^ | 0.5 | 0.25 | 0.19 | 0.75 | 1 | 0.5 |
| 10^-5^ | 0.25 | 0.38 | NG | 0.38 | NG | NG |
| Sin fago | >256 | >256 | >256 | >256 | >256 | >256 |
|  | **MW2** | | | | | |
|  | **ΦStaph1N** | | | **Evo2** | | |
| **MOI** | **Rep 1** | **Rep 2** | **Rep 3** | **Rep 1** | **Rep 2** | **Rep 3** |
| 10^-2^ | 3 | 24 | 24 | 4 | NG | NG |
| 10^-3^ | 32 | 24 | 48 | 4 | NG | NG |
| 10^-4^ | 48 | 96 | 32 | 3 | NG | NG |
| 10^-5^ | 96 | 64 | 24 | 2 | NG | NG |
| Sin fago | 96 | 48 | 32 | 96 | 48 | 32 |
|  | **LAC** | | | | | |
|  | **ΦStaph1N** | | | **Evo2** | | |
| **MOI** | **Rep 1** | **Rep 2** | **Rep 3** | **Rep 1** | **Rep 2** | **Rep 3** |
| 10^-2^ | NG | NG | 2 | 0.064 | NG | NG |
| 10^-3^ | NG | 3 | 1.5 | 0.032 | NG | NG |
| 10^-4^ | 32 | 1.5 | 1 | NG | NG | NG |
| 10^-5^ | 32 | 16 | 0.38 | NG | NG | NG |
| Sin fago | 32 | 48 | 48 | 32 | 48 | 48 |

NG: no se detectó crecimiento

**Descubrimiento de un ΦStaph1N mutante con mejor actividad lítica contra SAMR**

En el caso de ΦStaph1N, observamos que, si bien el fago puede infectar las tres cepas de SARM, su eficiencia de formación de placas es menor en las cepas MW2 y LAC (Figura 1B, Figura 1- Figura suplementaria 1). Las placas de ΦStaph1N en los céspedes bacterianos de MW2 y LAC eran turbias, y la eficiencia de formación de placas fue aproximadamente dos órdenes de magnitud menor que la de MRSA252. Sorprendentemente, observamos consistentemente placas más pequeñas y claras que surgían en las placas más grandes y turbias de LAC (Figura 2- Figura suplementaria 1); esto no ocurrió en MW2. Planteamos la hipótesis de que estas placas claras eran causadas por una forma mutante de ΦStaph1N que desarrolló una mayor actividad lítica. Aislamos clones de fagos de estas placas individuales y evaluamos su actividad contra SARM. Este fago mutante, al que llamamos Evo2, forma placas en las cepas LAC y MW2 con mayor eficiencia, mostrando una eficiencia comparable a la de MRSA252 (Figura 2A). En experimentos de curvas de crecimiento, observamos además que Evo2 lisa cultivos de SARM a MOI más bajos en comparación con ΦStaph1N (Figura 2- Figura suplementaria 2). Evo2 exhibe actividad lítica contra MW2 y LAC incluso a un MOI de 10⁻^4^, una concentración a la que ΦStaph1N no muestra actividad detectable contra ambas cepas.

Secuenciamos el genoma de Evo2 para determinar el mecanismo genético que impulsa esta mayor actividad. Observamos una única mutación puntual en ORF141 que induce un codón de terminación prematuro (Figura 2- Figura suplementaria 1). El análisis de secuencia con HHpred predice que ORF141 es una posible proteína que enlaza ADN con un motivo proteico HTH (PDB: 2LVS, valor E: 2,5e-9). Especulamos que esta proteína es un regulador transcripcional que, al ser inactivada por una mutación sin sentido, aumenta la infectividad de ΦStaph1N. Estudios futuros se centrarán en determinar el mecanismo de esta mutación y por qué Evo2 solo evolucionó en LAC.

Dada la mayor actividad de Evo2 contra SARM, nos preguntamos si la depredación por Evo2 afecta la resistencia a los β-lactámicos. Infectamos MRSA252, MW2 y LAC con Evo2 a un MOI de 0.1 y medimos las CMI contra los β-lactámicos tras 48 horas. Al igual que con ΦStaph1N, la infección por Evo2 redujo las CMI de las tres cepas de SARM contra OXA, CEF y AMX+CA, mientras que las CMI contra AMX solo y VAN no variaron significativamente (Figura 2B). Ampliando el análisis de las diferentes clases de antibióticos, evaluamos si la depredación por Evo2 podría afectar la susceptibilidad al inhibidor de transcripción rifampicina; a los inhibidores de traducción eritromicina y mupirocina; y a los disruptores de la envoltura celular teicoplanina, fosfomicina y daptomicina (Tabla 2). Observamos que las CMI de estos antibióticos no cambiaron significativamente, con algunas excepciones: en algunos casos, LAC resistente a Evo2 se sensibilizó a fosfomicina y daptomicina; además, una réplica de MRSA252 resistente a Evo2 desarrolló sensibilidad a la teicoplanina. Sin embargo, en general, la reducción de la CMI en estos casos no fue tan drástica como la observada frente a los β-lactámicos.

**Tabla 2. Concentración mínima inhibitoria (µg/mL) de diferentes antibióticos contra SARM tratado con Evo2 y sin tratamiento de fago**

|  |  | **Sin fago** | | | **Evo2** | | |
| --- | --- | --- | --- | --- | --- | --- | --- |
| **Cepa** | **Antibiótico** | **Rep 1** | **Rep 2** | **Rep 3** | **Rep 1** | **Rep 2** | **Rep 3** |
| MRSA252 | Oxacilina | >256 | >256 | >256 | 0.38 | 0.75 | 0.5 |
|  | Rifampicina | 0.047 | 0.032 | 0.012 | 0.023 | 0.047 | 0.023 |
|  | Mupirocina | 0.75 | 0.5 | 1 | 0.5 | 0.5 | 0.38 |
|  | Eritromicina | >256 | >256 | >256 | >256 | >256 | >256 |
|  | Teicoplanina | 6 | 6 | 4 | 4 | 4 | 0.75 |
|  | Fosfomicina | 12 | 8 | 8 | 8 | 8 | 6 |
|  | Daptomicina | 2 | 3 | 2 | 2 | 2 | 2 |
| MW2 | Oxacilina | 48 | 32 | 32 | 0.75 | 1 | 0.75 |
|  | Rifampicina | 0.032 | 0.047 | 0.064 | 0.023 | 0.023 | 0.032 |
|  | Mupirocina | 0.5 | 0.25 | 0.5 | 0.38 | 0.38 | 0.25 |
|  | Eritromicina | 1 | 0.75 | 0.75 | 0.25 | 0.5 | 0.25 |
|  | Teicoplanina | 2 | 1.5 | 1.5 | 1.5 | 1 | 1 |
|  | Fosfomicina | 1 | 1.5 | 1.5 | 0.5 | 1 | 1 |
|  | Daptomicina | 1.5 | 2 | 1.5 | 0.75 | 0.5 | 3 |
| LAC | Oxacilina | 48 | 24 | 64 | 0.19 | 0.064 | 0.047 |
|  | Rifampicina | 0.047 | 0.047 | 0.047 | 0.032 | 0.032 | 0.047 |
|  | Mupirocina | 0.5 | 0.75 | 0.75 | 0.5 | 0.5 | 0.5 |
|  | Eritromicina | 3 | 3 | 3 | 1.5 | 1 | 2 |
|  | Teicoplanina | 1 | 0.5 | 1 | 0.5 | 0.5 | 0.75 |
|  | Fosfomicina | 6 | 6 | 12 | 1.5 | 12 | 1 |
|  | Daptomicina | 0.5 | 3 | 1 | 0.064 | 2 | 0.75 |

Rep = replica biológica

Finalmente, examinamos cómo diferentes MOI de Evo2 afecta la resistencia a los β-lactámicos en SARM (Tabla 1). Infectamos las tres cepas de SARM con Evo2 a diferentes MOI, de 10^-2^ a 10^-5^, y medimos la CMI de oxacilina del SARM evolucionado. En las tres cepas, observamos que algunas replicas de los cultivos con diferentes MOI no recuperaron el crecimiento tras la infección con Evo2. Sin embargo, los cultivos de MRSA252, MW2 y LAC que sí volvieron a crecer mostraron una pérdida de resistencia a la oxacilina de entre 10 y 1000 veces. Por lo tanto, en general, Evo2 mostró una mayor infectividad contra el SARM y una mayor potencia para reducir la resistencia a los β-lactámicos.

**Evo2 es ampliamente lítico contra aislados clínicos recientes de *S. aureus* USA300**

MRSA252, MW2 y LAC se aislaron en 1997, 1998 y a principios de la década de 2000, respectivamente. Por lo tanto, probamos si Evo2 puede infectar aislados clínicos de *S. aureus* más recientes. Comparamos la eficiencia de formación de placas de Evo2 y ΦStaph1N contra 30 cepas USA300 que se aislaron entre 2008 y 2011 en el St. Louis Children's Hospital.^26^ Observamos una variación drástica en la eficiencia de formación de placas de ΦStaph1N en las 30 cepas, mientras que Evo2 exhibió una mayor eficiencia de formación de placas en la mayoría de las 30 cepas (Tabla 3). Luego probamos si infección de Evo2 afecta a la resistencia a OXA en 12 (ADL1-12) de estos aislados clínicos. Infectamos las cepas con Evo2 a una MOI de 0.1 y, si se recuperaba una población resistente al fago, medimos la CMI de OXA tras 48 horas. Curiosamente, tras 15 desafíos independientes con Evo2, no pudimos recuperar poblaciones resistentes a Evo2 de ADL1, 5, 6 y 12. Esto sugiere que la adquisición de resistencia a Evo2 es poco frecuente en estas cepas. En el resto de las cepas, observamos que, al igual que con MRSA252, MW2 y LAC, la CMI de OXA se redujo entre 10 y 100 veces tras la infección con Evo2 (Figura 2C). En general, estos resultados destacan la mayor variedad de hospedadores y la mayor actividad de Evo2.

**Tabla 3. Eficiencia de formación de placas (EOP)^*^ de ΦStaph1N, Evo2, and ΦNM1γ6 en aislados clínicos del tipo USA300 (ADL1-30).**

| **Strain** | **ΦStaph1N** | **Evo2** | **ΦNM1γ6** |
| --- | --- | --- | --- |
| RN4220 | 1.0E+00 | 1.0E+00 | 1.0E+00 |
| ADL1 | 1.7E-02 | 2.7E+00 | 6.7E-01 |
| ADL2 | 2.0E-01 | 3.3E+00 | 1.0E+00 |
| ADL3 | 6.0E-02 | 2.0E+00 | 1.7E-02 |
| ADL4 | 1.3E-03 | 1.0E+00 | 1.7E-01 |
| ADL5 | 1.2E-02 | 2.7E+00 | 1.0E+00 |
| ADL6 | 1.5E-02 | 1.7E+00 | 1.0E+00 |
| ADL7 | 6.0E-01 | 1.7E+00 | 6.7E-01 |
| ADL8 | 9.0E-02 | 2.0E+00 | 3.3E-01 |
| ADL9 | 6.0E-03 | 1.3E+00 | 2.0E-04 |
| ADL10 | 5.0E-01 | 1.3E+00 | 1.0E-03 |
| ADL11 | 1.0E+00 | 1.0E+00 | 3.3E-01 |
| ADL12 | 9.0E-04 | 2.0E+00 | 6.7E-07 |
| ADL13 | 7.0E-02 | 2.7E+00 | 2.3E-01 |
| ADL14 | 2.7E-01 | 3.3E+00 | 1.0E-05 |
| ADL15 | 9.0E-02 | 3.0E+00 | 2.0E-02 |
| ADL16 | 2.4E-01 | 5.6E+00 | 7.8E-02 |
| ADL17 | 3.3E+00 | 1.0E+01 | 2.4E+00 |
| ADL18 | 6.7E+00 | 1.4E+01 | 1.6E-02 |
| ADL19 | 7.1E-01 | 1.6E+00 | 2.3E+00 |
| ADL20 | 6.2E+00 | 7.8E+01 | 2.4E+00 |
| ADL21 | 5.2E+00 | 5.6E+00 | 3.3E-06 |
| ADL22 | 9.5E-01 | 2.0E+00 | 5.2E-01 |
| ADL23 | 1.5E+00 | 3.3E+01 | 1.2E-01 |
| ADL24 | 7.1E-01 | 5.6E+00 | 3.3E-06 |
| ADL25 | 7.1E-01 | 1.8E+00 | 2.3E-01 |
| ADL26 | 2.4E-02 | 3.9E+00 | 2.2E-03 |
| ADL27 | 1.3E+00 | 7.8E+00 | 1.7E+00 |
| ADL28 | 1.9E+00 | 8.9E+00 | 8.9E-02 |
| ADL29 | 1.9E+00 | 1.0E+01 | 1.3E-04 |
| ADL30 | 6.7E-01 | 1.4E+00 | 1.7E-05 |

*EOP en los aislados clínicos fueron estandarizados con sus respectivos EOP en la cepa de *S. aureus* RN4220

**Efecto de otros fagos estafilocócicos en la CMI de β-lactámicos**

Nos planteamos si dos fagos adicionales de nuestra colección podrían inducir una reducción en la CMI de oxacilina en SARM. La cepa SARM LAC es sensible a la infección por ΦNM1γ6, una versión lítica del fago templado ΦNM1 del género Dubowvirus, derivado de la cepa *S. aureus* Newman^27-29^. En LAC, ΦNM1γ6 presenta una formación de placas comparable a la de ΦStaph1N, a la vez que muestra actividad contra algunos de los aislados clínicos USA300 (Figura 1-Figura suplementaria 1, Tabla 3). Por lo tanto, infectamos LAC con ΦNM1γ6 a una MOI de 0.1 y medimos la CMI de β-lactámicos de las células sobrevivientes. Si bien los cultivos de LAC recuperados mostraron resistencia a ΦNM1γ6, no mostraron una reducción en la CMI (Figura 2-Figura suplementaria 3), lo que sugiere que la resistencia a los fagos causada por FNM1g6 no está asociada a la resistencia a β-lactámicos. También aislamos del ambiente un segundo fago del género Kayvirus, denominado SATA8505^30^, y evaluamos su actividad contra SARM. SATA8505 es activo contra MRSA252, MW2 y LAC (Figura 2-Figura suplementaria 4), y la infección de las tres cepas provocó un aumento de la resistencia a SATA8505 en MRSA252, MW2 y LAC. Al igual que en el caso de ΦStaph1N y Evo2, las células resistentes a SATA8505 mostraron una marcada pérdida de resistencia a la oxacilina (Figura 2-Figura suplementaria 4). En conjunto, nuestros resultados con estos dos fagos sugieren que la capacidad de reducir la resistencia a los β-lactámicos no es una característica universal en todos los fagos estafilocócicos y exige un análisis más exhaustivo de los fagos estafilocócicos y su capacidad para obtener un efecto en la resistencia a los β-lactámicos.

**Mutaciones genómicas en cepas de SARM tras la infección con fagos**

Tras nuestros análisis fenotípicos, examinamos los genomas de las cepas de SARM resistente a fagos. Primero, secuenciamos los genomas de tres aislados clonales (A-C) de cada cepa de SARM sometida a ΦStaph1N, Evo2 o sin infección por fagos. Observamos que cada cepa de SARM desarrollaron mutaciones distintas (Figura 3-Figura suplementaria 1). Independientemente de la cepa y el tratamiento con fagos, encontramos que la mayoría de las mutaciones causan sustituciones de aminoácidos, seguidas de truncamientos (Figura S6). Las variantes del Grupo de Genes Ortólogos (*Cluster of Orthologous Genes*, COG) asociadas con la transcripción, la biogénesis de la pared/membrana/envoltura celular, el transporte y metabolismo de coenzimas, y los mecanismos de defensa fueron las categorías más comunes. Las mutaciones en genes anotados que aparecieron al menos dos veces en las réplicas clonales se resumen en la Tabla 4. La información sobre todas las variantes genéticas detectadas están en el Archivo suplementario 1.

**Tabla 4. Genes mutados en SARM después de infección con los fagos ΦStaph1N or Evo2**

| **Gen** | **Descripción** | **Cepa** | **Tratamiento** | **Referencia** |
| --- | --- | --- | --- | --- |
| *sarA* | Regulador transcripcional de resistencia a antibióticos y virulencia | MW2 | Evo2 | ^31,32^ |
| *mgrA* | Regulador transcripcional de resistencia a antibióticos y virulencia | MW2 | ΦStaph1N, Evo2 | ^33,34^ |
| *rpoB* | Subunidad beta de la polimerasa de ARN  Regulador transcripcional de resistencia a antibióticos | LAC | ΦStaph1N, Evo2 | ^35^ |
| *arlR* | Regulador transcripcional de resistencia a antibióticos y virulencia | LAC | ΦStaph1N, Evo2 | ^34,36,37^ |
| *spoVG* | Regulador transcripcional de resistencia a antibióticos y virulencia | LAC | ΦStaph1N, Evo2 | ^38,39^ |
| *cysE* | Síntesis de cisteína y metionina, serina O-acetiltransferasa | MW2 | Evo2 | ^40^ |
| *metK* | Síntesis de cisteína y metionina, sintetasa  S-adenosilmetionina (SAM) | MW2 | ΦStaph1N, Evo2 | ^41^ |
| *trpF* | Síntesis de fenilalanina, tirosina and triptófano, isomerasa fosforibosilantranilato | LAC | ΦStaph1N, Evo2 | ^42^ |
| *femA* | Síntesis de peptidoglícano, síntesis de pentaglicina | MRSA252 | ΦStaph1N, Evo2 | ^43,44^ |
| *murE* | Síntesis de peptidoglícano, síntesis del tripéptido UDP-MurNAc | MW2 | ΦStaph1N, Evo2 | ^45^ |
| *trpS* | Síntesis de aminoacil-ARNt, síntesis de triptofanil-ARNt | MW2 | ΦStaph1N, Evo2 | ^46^ |
| *ytqA* | Modificaciones de ARNt, síntesis de mnm^5^s^2^U | MW2 | ΦStaph1N, Evo2 | ^47^ |
| *yvcD* | Desconocido | MW2 | Evo2 |  |
| *natA* | Transportador ABC | MW2 | ΦStaph1N, Evo2 | ^48^ |
| *tcaB* | Bomba de eflujo multifármaco (predicción) | MW2 | ΦStaph1N, Evo2 | ^49^ |
| *fmhC* | Factor parecido a Fem | LAC | ΦNM1γ6 | ^50^ |
| *rsaC* ncRNA | modula la respuesta al estrés oxidativo y la inmunidad a los metales | MW2 | ΦStaph1N + oxacilina | ^51^ |
| *nrdF* | subunidad beta de la ribonucleósido-difosfato reductasa de clase 1b; implicada en la síntesis de ADN | MW2 | ΦStaph1N + oxacilina | ^52^ |
| *fstAT* ncRNA | Desconocido | MW2 | ΦStaph1N + oxacilina |  |
| *rpoC* | Subunidad beta’ de la polimerasa de ARN | MW2 | ΦStaph1N + oxacilina |  |
| tRNA | ARN de transferencia | MW2 | ΦStaph1N + oxacilina |  |

Una hipótesis para explicar la pérdida de resistencia a los β-lactámicos es que la infección por fagos seleccionó a *SCCmec* o *blaZ* defectuosos. Sin embargo, no se observaron mutaciones en ninguno de los locus. En cambio, todas las cepas de SARM presentaron mutaciones en genes auxiliares implicados en la pérdida de resistencia a los β-lactámicos. En MRSA252, tanto la infección por ΦStaph1N como por Evo2 seleccionó a mutaciones sin sentido en el gen *femA*, que inactivarían el producto proteico. Como se mencionó anteriormente, *femA* es necesario para la síntesis de pentaglicina en el lípido II de *S. aureus*, el precursor del peptidoglicano (Tabla 4). Se ha demostrado que la eliminación de *femA* aumenta la susceptibilidad a los β-lactámicos incluso cuando se expresa PBP2a (codificada por *mecA*), lo que proporciona un mecanismo genético que explica cómo MRSA252 pierden la resistencia a las β-lactámicas tras la selección de fagos.^43,44^ Al mismo tiempo, se detectó la presencia de otras mutaciones no caracterizadas en MRSA252 resistente a fagos. Por ejemplo, el clon A de células tratadas con ΦStaph1N presentaba dos mutaciones: una mutación sin sentido en *femA* y una mutación de sustitución en una proteína no caracterizada; por otro lado, el clon B mostró una mutación de sustitución en *pfkA*, una 6-fosfofructoquinasa dependiente de ATP , y una mutación en una región intergénica; el clon C mostró una mutación por sustitución en una posible proteína de transporte, denominada *yueF* (Figura 3B, Archivo suplementario 1). Se desconoce el papel de estas mutaciones en la mediación de la resistencia a los fagos o la sensibilidad a los β-lactámicos, si existe alguna.

En MW2, encontramos mutaciones en dos reguladores transcripcionales, *mgrA* y *sarA* (Figura 3A, Tabla 4). Tanto *mgrA* como *sarA* pertenecen a la familia de proteínas MarR (regulador de resistencia múltiple a antibióticos)/SarA (regulador accesorio estafilocócico A), que regulan la resistencia a antibióticos y la virulencia en *S. aureus*.^31–33^ En MW2 tratado con ΦStaph1N, solo *mgrA* mutó, mientras que en MW2 tratado con Evo2, los clones también mostraron mutaciones sin sentido en *sarA*. También se encontraron mutaciones en *metK* e *ytqA*, ambos asociados con S-adenosilmetionina (SAM): *metK* sintetiza SAM, mientras que *ytqA* pertenece a la familia de enzimas radicales SAM y se predice que participa en la modificación del ARNt.^41,47^ MW2 tratado con fagos también mostró mutaciones en otros genes, como *tcaB* y *murE*. Cabe destacar que cada réplica clonal presentó múltiples mutaciones en el genoma, mientras que, en contraste, las células MW2 no tratadas solo mostraron una deleción en una región intergénica que no está presente en ninguna de las muestras tratadas con fagos. Estos hallazgos sugieren que MW2 podría estar acumulando múltiples mutaciones durante la infección por fagos.

En el caso de LAC, observamos un tercer patrón mutacional distinto (Figura 3A). Cabe destacar que encontramos mutaciones sin sentido en *arlR*, que forma parte del sistema de señalización de dos componentes *arlRS* (Tabla 4). La actividad de *arlRS* se ha relacionado con la virulencia, patogenicidad y resistencia a la oxacilina de *S. aureus*.^36,37^ Además, observamos mutaciones de sustitución de aminoácido en *spoVG*, un factor de transcripción que regula la expresión de genes implicados en diversas funciones, incluido el metabolismo de la pared celular (Tabla 4).^38^ De hecho, *spoVG* activa la expresión de femA.^39^ Estudios han demostrado que *spoVG* modula la resistencia a los antibióticos β-lactámicos mediante la modulación de la síntesis de la pared celular.^39^ Al igual que las otras dos cepas de SARM, los clones de LAC tratados con fagos mostraron múltiples mutaciones en sus genomas. Observamos mutaciones en *prsA* y *bioA* que aparecieron tanto en las condiciones sin tratamiento de fagos como en las de tratamiento con fagos, lo que sugiere que estas mutaciones no surgen debido a la selección de fagos.

En conjunto, nuestros resultados muestran que las cepas de SARM infectadas con fagos adquieren perfiles mutacionales distintos. Es probable que estas mutaciones actúen juntas para promover la resistencia a los fagos y la sensibilidad a los β-lactámicos, lo que dificulta determinar las contribuciones mecanísticas de cada mutación. Por ejemplo, observamos que los genes *mgrA*, *sarR* y *arlR* desarrollaron mutaciones sin sentido, lo que resultaría en productos proteicos truncados y potencialmente no funcionales. Por lo tanto, evaluamos si la eliminación de estos genes por sí solo es suficiente para conferir resistencia a ΦStaph1N y Evo2 (Figura 3-Figura suplementaria 2). En MW2, la eliminación de *mgrA* resultó en una modesta reducción de la formación de placas en Evo2 y ΦStaph1N. Sin embargo, ninguno de los mutantes restantes, ni en la cepa de MW2 ni en el de LAC, confirió resistencia. Estudios experimentales previos también han demostrado que la resistencia a los fagos en *S. aureus* puede surgir de la alteración de genes individuales directamente implicados en la síntesis y modificación de WTA, como *tagO*.^51^ Algunas cepas de SARM también alteran la glicosilación de la pared celular a través de genes específicos codificados en profagos.^14^ Sin embargo, no observamos mutaciones en genes directamente implicados en la síntesis de WTA. Por lo tanto, nuestros resultados destacan cómo diferentes cepas de SARM puede adoptar vías mutacionales únicas bajo selección de fagos.

Finalmente, examinamos las mutaciones en poblaciones de LAC resistentes a ΦNM1γ6. Dado que la infección con ΦNM1γ6 no redujo la resistencia a OXA, planteamos la hipótesis de que las mutaciones que surgieron en LAC tras ΦNM1γ6 serían distintas de las que surgieron tras la infección con Evo2. Descubrimos que los mismos dos genes mutaron en tres aislados clonales de diferentes poblaciones resistentes: *bioA* y *fmhC*. Como se observó previamente, las mutaciones en *bioA* aparecieron en el tratamiento sin fagos, lo que sugiere que surgieron independientemente de la selección de fagos. Por otro lado, LAC mostró una mutación de sustitución de aminoácido en *fmhC* (H21D, Tabla 4, Archivo suplementario 1). FmhC y su homólogo FmhA trabajan con FemA y FemB para incorporar dipéptidos Gly-Ser en los entrecruzamientos entre capas de peptidoglicano.^50^ Sin embargo, se desconoce el mecanismo de la mutación H21D y, hasta donde sabemos, las mutaciones en *fmhC* no se han asociado previamente con la resistencia a los fagos en *S. aureus*.

**Las cepas de SARM tratadas con fagos presentan amplios cambios en sus transcriptomas**

Nuestro análisis genómico reveló que las cepas de SARM tratadas con fagos desarrollaron mutaciones en diversos reguladores transcripcionales, algunos de los cuales se sabe que afectan virulencia. Por lo tanto, planteamos la hipótesis de que las mutaciones en estos reguladores alterarían significativamente el perfil transcripcional de las cepas de SARM. Para comprobarlo, realizamos experimentos de secuenciación de ARN en las cepas MW2 y LAC tratadas con el fago Evo2 y comparamos sus perfiles de transcripción con los de las cepas no tratadas con fago (Figura 4, Archivo suplementario 2). Observamos cambios significativos en la expresión génica tanto en MW2 como en LAC. Cabe destacar que, siguiendo la tendencia observada en los datos mutacionales, no observamos cambios significativos en la expresión de genes en el locus *SCCmec* ni en *blaZ* presentes en MW2 y LAC.

Primero comparamos nuestros datos de expresión con estudios transcriptómicos previos. Por ejemplo, Horswill y sus colegas demostraron que la deleción de *arlRS* y *mgrA* aumenta la expresión de la proteína de unión a la matriz extracelular *ebh*. Además, estas deleciones también aumentaron la expresión de los genes de la ureasa involucrados en el ciclo de la urea TCA.^33,37^ En nuestros experimentos, tanto las cepas MW2 como LAC desarrollaron mutaciones sin sentido en *mgrA* y *arlR*, respectivamente. Por lo tanto, planteamos la hipótesis de que estas mutaciones imitarían los efectos de la deleción de genes y, asimismo, producirían niveles elevados de transcripción de los genes *ebh* y *ureasa*. En consonancia con nuestra hipótesis, los datos de expresión diferencial tanto de MW2 como de LAC mostraron cambios log2 de >7 para el gen *ebh* y >3 para los genes *ureABCDEFG* (Archivo suplementario 2).

Además, tanto MW2 como LAC aumentaron considerablemente la expresión de varios genes implicados en el mantenimiento de la pared celular. Estos incluyen *lytN*, una hidrolasa de mureína implicada en el compartimento de la pared de *S. aureus*, y *fmhC*, que, como se describió previamente, incorpora dipéptidos Gly-Ser en los entrecruzamientos de pentaglicina en la pared celular del peptidoglicano de *S. aureus*. Se ha demostrado que la sobreexpresión de *lytN* daña la pared celular, lo cual a su vez se alivia con la sobreexpresión de *fmhC*.^50^ Curiosamente, la sobreexpresión de *fmhC* está relacionada con una mayor sensibilidad a los β-lactámicos y, por lo tanto, podría contribuir a la pérdida de los fenotipos de resistencia a los β-lactámicos que observamos en las cepas de SARM.

MW2 y LAC también inhibieron la expresión de numerosos genes, muchos de los cuales son factores de virulencia. Ambas cepas redujeron los niveles de transcripción de genes en el locus del sistema de secreción tipo VII (locus *ess*), la biosíntesis de estafiloxantina (*crtM*, *crtN*, *crtP*) y la percepción de cuórum (p. ej., agrA) (Figura 4, Archivo suplementario 2). Individualmente, se ha demostrado que estos genes refuerzan la capacidad de *S. aureus* para establecer la infección y evadir el sistema inmune del huésped.^52-54^ Nuestros resultados sugieren que la infección por Evo2 puede provocar que SARM reduzca la expresión de todas estos genes, lo que podría reducir la virulencia de *S. aureus*.

Finalmente, observamos que tanto MW2 como LAC mostraron cambios transcripcionales que parecen ser específicos de cada cepa. Por ejemplo, en MW2 se observó un aumento significativo del factor de virulencia *spa* (gen codificante de proteína A), conocido por interferir con la respuesta inmunitaria del huésped e interactuar con otras especies bacterianas. También se ha demostrado que la presencia de proteína A unida a la pared celular disminuye la absorción de fagos, probablemente al enmascarar el WTA.^55^ Además, en MW2, observamos que *tarM*, que añade α1,4-GlcNAc al WTA, mostró una fuerte sobreexpresión (proporción log2 = 6.63). Esto concuerda con hallazgos previos que indican que niveles elevados de tarM y α1,4-GlcNAc-WTA pueden provocar resistencia a fagos en SARM. El LAC mostró un aumento en la expresión (proporción log2 >3,8) de los genes de la citotoxina hemolítica lukD/E, que lisa las células huésped, entre ellas a los neutrófilos.^56^ Se desconoce si el aumento de la expresión de estos genes resulta en un mayor nivel de producción y secreción de proteínas, pero estos cambios transcripcionales podrían representar una posible compensación evolutiva con consecuencias negativas para la clínica. Se necesitarán estudios adicionales para evaluar completamente los efectos fisiológicos y ecológicos de estos genes sobreexpresados ​​en SARM. En conjunto, nuestros resultados de secuenciación de ARN sugieren que la infección por fagos y la resistencia en SARM causan cambios transcripcionales significativos en una amplia gama de genes de virulencia, metabólicos y asociados a la pared celular.

**Las cepas de SARM tratadas con fagos presentan fenotipos de virulencia reducidos.**

*S. aureus* es un patógeno altamente virulento que depende de una amplia gama de toxinas y proteínas de evasión inmunitaria para promover infección.^57^ Las cepas MW2 y LAC son utilizados como modelos de virulencia de SARM**.**^33,34^ A la luz de nuestros datos mutacionales y transcriptómicos, planteamos la hipótesis de que las células de SARM tratadas con fagos presentarían fenotipos de virulencia alterados, además de una menor resistencia a β-lactámicos. Primero, evaluamos la capacidad de las cepas de SARM que sobrevivieron a la depredación por fagos para formar biopelículas en un ensayo de Cristal Violeta. Observamos que la infección de MRSA252 con Evo2 resultó en una reducción significativa en la absorción de Cristal Violeta en comparación con la cepa parental, lo que muestra una disminución en formación de biopelículas. Sin embargo, no mostramos diferencias significativas en la absorción de Cristal Violeta entre las cepas MW2 y LAC parentales, las tratadas con placebo y las tratadas con fagos (Figura 5-Figura suplementaria 1).

Luego, evaluamos si la infección por fagos podría afectar la hemólisis de las células sanguíneas de conejo. La hemólisis está mediada por la secreción de toxinas, en particular la hemolisina alfa codificada por el gen *hla*, y desempeña un papel importante en la infección de SARM.^58^ La expresión de estas toxinas está regulada por vías de señalización de virulencia que comprenden numerosos factores de transcripción, como *mgrA*, *arlR* y *sarA*. Además, en nuestros resultados de secuenciación de ARN, observamos que las cepas de SARM resistentes a los fagos mostraron una expresión reducida de otras citotoxinas. Las colonias parentales de MW2 y LAC lisaron células sanguíneas de conejo en platos de agar de sangre, produciendo halos distintivos de aclaramiento alrededor de las células bacterianas. En el caso de LAC sin tratamiento de fago, el área total de hemólisis fue, en promedio, tres veces mayor que la de MW2 sin tratamiento (~210 mm² frente a ~70 mm², respectivamente); en cambio, con MRSA252 no se detectó lisis (Figura 5A). Tras el tratamiento con ΦStaph1N, observamos que MW2 y LAC presentaron una reducción del área de hemólisis de 4 a 5 veces. Con las células tratadas con Evo2, observamos que en MW2 la reducción fue comparable a la de las células tratadas con ΦStaph1N. Sin embargo, en LAC tratada con Evo2, la pérdida de hemólisis fue aún más pronunciada, con dos réplicas sin hemólisis detectable. Observamos que ni MW2 ni LAC mostraron una reducción en la expresión de la transcripción de *hla*. Postulamos que la pérdida de hemólisis podría deberse a la incapacidad del SARM tratado por fagos de secretar la toxina.

Finalmente, analizamos cómo la infección por fagos afecta la aglutinación celular (o agregación, *clumping* en inglés) en SARM. *S. aureus* se enlaza al fibrinógeno y forma agregados bacterianos. Se cree que esta agregación tiene varias funciones en el contexto de las infecciones estafilocócicas, facilitando la adhesión al tejido del huésped. También es probable que las agregaciones sean más resistentes a la eliminación por el sistema inmune, en parte porque pueden ser demasiado grandes para ser fagocitadas por los neutrófilos.^34^ En nuestros datos transcripcionales, observamos que varias proteínas de la superficie celular conocidas por reducir la agregación celular, como *ebh*, fueron sobreexpresadas en SARM resistente a fagos. Horswill y sus colegas descubrieron que la desrepresión de *ebh* reduce la agregación. De hecho, MW2 y LAC tratadas con fagos mostraron menos agregación que la cepa con tratamiento sin fago o la cepa parental. En el caso de MW2, la infección con ΦStaph1N produjo una reducción moderada, mientras que la infección con Evo2 resultó en una reducción de aproximadamente el triple (Figura 5B). En LAC, observamos que tanto el tratamiento con ΦStaph1N como con Evo2 produjo reducciones comparables de la agregación en las células supervivientes. En general, estos resultados fenotípicos coinciden con nuestros datos genéticos y transcriptómicos, lo que demuestra que la infección por fagos puede conducir a poblaciones de SARM a fenotipos de virulencia reducida.

**Tratamiento combinado de Evo2 y β-lactámicos**

Los resultados anteriores sugieren que las células de SARM desarrollan resistencia a los fagos tras la infección, lo que se asocia a compensaciones en la virulencia y la resistencia a β-lactámicos. A continuación, nos preguntamos cómo evolucionarían las poblaciones de SARM bajo cotratamiento de fagos y β-lactámicos. En principio, estas dos presiones selectivas simultáneas podrían impulsar la evolución de la resistencia tanto al fago como al antibiótico, anulando las compensaciones en la resistencia a antibioticos. Para comprobarlo, realizamos ensayos con cotratamiento de fagos y oxacilina en MRSA252, MW2 y LAC. Se mezclaron diluciones seriadas de Evo2 o ΦStaph1N con diluciones seriadas de oxacilina en una placa de 96 pocillos (Figura 6A), tras lo cual se añadieron las cepas de SARM a la placa y se dejaron crecer durante 24 horas. Tras 24 horas, se transfirió el 1% del cultivo de cada pocillo a un pocillo de placa nuevo con medio no selectivo, y se dejó crecer durante 24 horas más (48 horas en total). Durante el experimento, se monitoreó la densidad celular midiendo la densidad óptica en cada pocillo de la placa.

Primero examinamos el crecimiento del SARM en combinaciones de Evo2 y antibiótico (Figura 6A, fila superior). En el caso de MRSA252, las células crecieron con niveles bajos de fago (MOI de 0,01 o inferior) y oxacilina (<0.125 µg/mL) (Figura 6A). LAC mostró mayor sensibilidad, sin crecimiento detectable después de 48 horas en presencia de fago, independientemente de la presencia de oxacilina. En el caso de MW2, las células mostraron un crecimiento limitado con MOI <1 y niveles de oxacilina <0.125 µg/mL. Para cada cepa, seleccionamos células de pocillos con una DO600 >0.5 y las inoculamos en medio fresco no selectivo. Como control, también regeneramos células que no fueron tratadas con fago ni oxacilina (pocillo B1). En el caso de MRSA252, dos pocillos (E2 y F2) contenían células SARM viables, que formaron cultivos turbios; en el caso de MW2, seis pocillos (D4, E2, E3, E5, F2 y F3) seleccionados volvieron a crecer (Figura 6B). Suponemos que las células de los cultivos fallidos presentaron una viabilidad reducida debido al tratamiento con fagos y antibióticos. Analizamos la susceptibilidad de estos cultivos regenerados a fagos y oxacilina. Como era de esperar, las células MRSA252 y MW2 de los pocillos de control B1 fueron sensibles a Evo2, pero resistentes a oxacilina. En el caso de MRSA252, las células de E2 y F2 fueron resistentes a la infección por Evo2 y mostraron una disminución de 1000 veces en la CMI frente a oxacilina. De forma similar, en MW2, los seis cultivos viables mostraron resistencia a Evo2 y una disminución de 10 a 100 veces en la CMI. En conjunto, estos resultados coinciden con los del SAMR que se sometió a una selección única con Evo2.

Luego, analizamos el crecimiento del SARM en las combinaciones de ΦStaph1N/oxacilina (Figura 6A, fila inferior). Ni MRSA252 ni LAC pudieron crecer en ninguna combinación de ΦStaph1N/oxacilina; por el contrario, MW2 creció en una amplia gama de combinaciones de ΦStaph1N/oxacilina. Cuando MW2 infectado con ΦStaph1N se trató con altos niveles de oxacilina (16 µg/mL), las células recuperadas (pocillos D11 y E11) no mostraron una reducción en la CMI frente a la oxacilina (Figura 6C). Sin embargo, la eficiencia de plaqueo del ΦStaph1N se redujo entre 3 y 6 órdenes de magnitud. La secuenciación de sus genomas reveló que estas células desarrollaron un conjunto único de mutaciones diferentes a las observadas en las condiciones de tratamiento con un solo fago (Tabla 4). Sin embargo, cuando las células MW2 infectadas con ΦStaph1N fueron tratadas simultáneamente con niveles bajos (<0,125 µg/mL) de oxacilina, las células recuperadas (C3) mostraron una fuerte resistencia a los fagos y una reducción de 100 veces en la resistencia a la oxacilina, lo que refleja los fenotipos observados en los experimentos de tratamiento con un solo fago.

Observamos que, de las tres cepas de SARM analizadas, MW2 es la menos sensible a la infección por ΦStaph1N. Postulamos que la presión selectiva ejercida por los altos niveles de β-lactámicos predomina sobre la presión selectiva de ΦStaph1N, lo que conduce a la evolución de células con resistencia continua a los β-lactámicos y resistencia limitada a los fagos. Sin embargo, a niveles más bajos de β-lactámicos, predomina la presión ejercida por la infección por fagos, lo que conduce al aumento de células con resistencia completa a los fagos y a una disminución en la resistencia a los β-lactámicos. Esta selección dependiente de la dosis por oxacilina no se observa cuando se utiliza un fago más activo (p. ej., Evo2). Aunque limitados a una cepa de SARM, estos resultados sugieren que diferentes combinaciones de fagos/β-lactámicos pueden producir resultados evolutivos divergentes en el SARM, cada uno con posibles implicaciones clínicas.

**Discusión**

En este trabajo, informamos del descubrimiento de que la infección por ciertos fagos puede impulsar a las poblaciones de SARM a desarrollar compensaciones evolutivas favorables entre la resistencia a fagos y β-lactámicos. Se ha propuesto aprovechar estas compensaciones para combatir la resistencia en patógenos bacterianos.^18^ Los tratamientos con fagos no solo podrían reducir la carga bacteriana de una infección, sino también potencialmente resensibilizar las poblaciones bacterianas a antibióticos a los que previamente eran resistentes. Demostramos que las cepas de SARM infectadas con los virus ΦStaph1N, Evo2 y SATA8505 desarrollaron resistencia a los fagos, pero presentaron una pérdida de hasta 1000 veces en sus CMI frente a los antibióticos β-lactámicos. Además, estos SARM evolucionados presentan fenotipos de virulencia reducida, como niveles más bajos de hemólisis y agregación. Nuestros hallazgos demuestran que los fagos pueden resensibilizar el SARM a los β-lactámicos e incluso disminuir su virulencia, resultados de gran valor biomédico. Sin embargo, no todos los fagos estafilocócicos pueden mediar estas compensaciones: la infección con ΦNM1γ6, un fago del género Dubowvirus, generó resistencia a los fagos en el SARM, pero no redujo la resistencia a los β-lactámicos. Una importante línea de investigación futura será determinar qué tipos de fagos generan estas compensaciones evolutivas beneficiosas en el SARM.

Nuestros resultados también presentan un panorama complejo de la evolución del SARM durante la infección por fagos. La secuenciación genómica reveló que las cepas de SARM desarrollaron perfiles de mutación distintos tras la infección, lo que sugiere una multitud de vías evolutivas que diferentes cepas bacterianas pueden seguir para desarrollar resistencia a los fagos. No solo las cepas de SARM desarrollaron mutaciones distintas, sino que clones individuales resistentes a los fagos acumularon múltiples mutaciones en su genoma. No obstante, las diferentes cepas de SARM mostraron convergencia de fenotipos en forma de resistencia a los fagos, menor resistencia a los β-lactámicos y menor virulencia. Postulamos que esta convergencia se debe a la participación de la pared celular en los tres resultados fenotípicos. Los fagos deben interactuar con la pared celular, los β-lactámicos se dirigen a proteínas asociadas con el mantenimiento de la pared celular, y muchos factores de virulencia de S. aureus están incrustados en la pared celular o se secretan a través de ella. Por lo tanto, cualquier modificación de la integridad o la composición química de la pared celular causada por la resistencia a los fagos afectará la sensibilidad y la virulencia de los β-lactámicos. El mantenimiento de la pared celular está controlado por numerosos genes, desde proteínas individuales implicadas en la síntesis de la pared celular, como *femA*, hasta reguladores transcripcionales, como *mgrA*, que controlan la expresión de genes que la sintetizan. Por lo tanto, los patrones mutacionales observados en cada cepa de SARM podrían reflejar soluciones genéticas que permiten a las bacterias adaptarse a la depredación de los fagos, a la vez que maximizan su aptitud para esa cepa en particular.

Sorprendentemente, el SARM moduló considerablemente los perfiles transcripcionales tras la infección por fagos. Creemos que estos perfiles de expresión alterados son consecuencia de las mutaciones genómicas que surgieron en los diversos reguladores transcripcionales. Observamos que las células evolucionadas inhibieron la expresión de genes implicados en la detección de cuórum, el sistema de secreción de tipo VII y diversas toxinas. Resulta interesante especular cómo inhibir la expresión de estos genes afecta las interacciones del SARM con otras bacterias que ocupan el mismo nicho ecológico y con el sistema inmunitario del huésped. Al mismo tiempo, las cepas de SARM también incrementaron la expresión de algunos factores de virulencia, como la proteína A , lo que podría representar una compensación evolutiva con efectos negativos o indeseables en la clínica.^59^ Los trabajos futuros se centrarán en evaluar el riesgo de estas compesasiones con posibles efectos negativos a la luz del beneficio clínico de la reducción de la resistencia y otros fenotipos de virulencia.

La resisteencia a antibióticos en patógenos bacterianos es un problema evolutivo y requerirá soluciones evolutivas para mitigarla. Nuestros hallazgos destacan la capacidad de los fagos para alterar drásticamente la evolución y la fisiología del SARM resistente a antibióticos. Ciertos tratamientos con fagos pueden forzar a las poblaciones bacterianas a seguir caminos evolutivos que las hacen vulnerables a los antibióticos o al sistema inmune del huésped. Fundamentalmente, esto permite la redistribución de agentes que de otro modo permanecerían ineficaces, ganando tiempo para el descubrimiento de nuevos fármacos. Por lo tanto, esperamos que nuestro trabajo pueda sugerir nuevas vías de investigación para nuevas estrategias de tratamiento basadas en fagos contra el SARM y otros patógenos resistentes a antibióticos.

**Agradecimientos**

MT cuenta con el apoyo monetario del programa SciMed GRS de la Universidad de Wisconsin-Madison; AJHV cuenta con el apoyo monetario del Programa de Becas de Investigación Graduada de la NSF (NSF-GRFP); CYM cuenta con el apoyo de fondos del Departamento de Bacteriología de la Universidad de Wisconsin-Madison y fondos de la Fundación Margaret Q. Landenberger. Agradecemos a la Dra. Wilmara Salgado-Pabón, a la Dra. Petra Levin y al Dr. Alexander Horswill por proporcionarnos cepas de SARM y por sus sugerencias. Agradecemos a los laboratorios Marraffini y Hatoum-Aslan por proporcionarnos muestras de bacteriófagos. Los análisis computacionales se realizaron mediante el Centro de Computación de Alto Rendimiento (*Center for High Throughput Computing*) de la Universidad de Wisconsin-Madison. También agradecemos a todos los miembros del laboratorio de Charlie Mo por su contribución científica.

**Contribuciones de los autores**

MT y AJHV contribuyeron equitativamente al artículo. MT, AJHV y CYM diseñaron el estudio; MT y AJHV realizaron todos los experimentos; PQT realizó el análisis bioinformático; EDL y LT aislaron y analizaron el bacteriófago SATA8505; CYM supervisó el proyecto. MT, AJHV y CYM redactaron el manuscrito, con la colaboración de los demás autores.

**Conflicto de interés**

MT, AJHV y CYM presentaron una patente provisional para el trabajo presentado en este documento a través de la Wisconsin Alumni Research Foundation (P240267US01).

**Figura 1**. **La infección por el bacteriófago ΦStaph1N impulsa la pérdida de resistencia a β-lactámicos en SARM.** **A.** Esquema del experimento. Cultivos bacterianos resistentes a fármacos (Abx^R^) y sensibles a fagos (Phage^S^) se infectan con fagos. La población de células infectadas se pasa y se deja recuperar. La población celular sobreviviente es resistente a la infección por fagos (Phage^R^), pero ha desarrollado sensibilidad a los antibióticos (Abx^S^). **B.** ΦStaph1N infecta las cepas de SARM MRSA252, MW2 y LAC (panel izquierdo). Tras la infección con ΦStaph1N, los cultivos evolucionados de las tres cepas de SARM son resistentes a ΦStaph1N (panel derecho). **C.** Las cepas de SARM evolucionadas tratadas con ΦStaph1N muestran una pérdida significativa de resistencia a la oxacilina, en comparación con las cepas parentales. La pérdida de resistencia se indica mediante el área de aclaramiento bacteriano que rodea la tira de resistencia a antibióticos. **D.** El tratamiento con ΦStaph1N causa pérdida de resistencia contra diferentes β-lactámicos. Se representan gráficamente las reducciones en la concentración mínima inhibitoria (MIC) entre las células tratadas y las células con tratamiento simulado. OXI = oxacilina; CEF = cefazolina; AMX = amoxicilina; AMX + CA = amoxicilina y ácido clavulánico; VANC = vancomicina.

**Figura 2. Evo2 es una variante de ΦStaph1N con mayor actividad contra SARM. A.** Evo2 muestra una infectividad comparable hacia MRSA252, pero una mayor infectividad hacia MW2 y LAC, en comparación con ΦStaph1N. **B.** Similar a ΦStaph1N, la infección por Evo2 reduce la resistencia a β-lactámicas en SARM. **C.** La infección por Evo2 reduce la CMI (MIC) contra oxacilina en aislados clínicos de USA300 (ADL).

**Figura 3. La infección por fagos de cepas de SARM produce perfiles mutacionales distintivos. A.** Secuencias codificantes (CDS) con mutaciones de las tres cepas de SARM tras el tratamiento con fagos o sin fagos. Para cada cepa, se secuenciaron tres aislados y se identificaron sus mutaciones. Las mutaciones se codifican por colores según el número de ocurrencias entre las tres réplicas. **B.** Categorías de genes con mutaciones que surgieron en cada cepa de SARM y condición de tratamiento.

**Figura 4. La infección por fagos modifica el perfil transcriptómico de SARM.** Se realizó un análisis de expresión diferencial en los transcriptomas de MW2 (panel superior) y LAC (panel inferior). Para ambas cepas, se compararon muestras infectadas con Evo2 con controles no infectados. Se analizaron tres réplicas biológicas para cada condición. Las líneas punteadas horizontales representan un valor de corte p ajustado de 0,002, mientras que las líneas punteadas verticales representan un cambio log² de -2 o 2 en la expresión. Los transcritos con un cambio log² entre -2 o 2 y un valor p >0,002 se etiquetan como puntos grises (no significativo, NS); los transcritos que superan el cambio de corte o el valor p (pero no el otro) se representan como puntos azules y verdes, respectivamente; los transcritos que superan ambos puntos de corte se muestran como puntos rojos. Los genes mencionados en el texto principal están etiquetados.

******

**Figura 5. El tratamiento con fagos del SARM produce fenotipos de virulencia atenuados. A.** Las cepas MW2 y LAC presentan actividad hemolítica en placas de agar con sangre de conejo, mientras que MRSA252 no. **B.** Las cepas MW2 y LAC tratadas con fagos presentan una hemólisis reducida en comparación con las células no infectadas. **C.** Los cultivos supervivientes de MW2 y LAC tratados con ΦStaph1N (azul) o Evo2 (rojo) muestran tasas de agregación reducidas en comparación con las células sin tratamiento (verde azulado).

**

**Figura 6. Cotratamiento de SARM con bacteriófago y β-lactámico. A.** Ensayos de tablero de ajedrez de cepas de SARM con gradientes de oxacilina y Evo2 (paneles superiores) o ΦStaph1N (paneles inferiores). El gradiente de oxacilina es una dilución seriada de 2 veces la concentración del fármaco (µg/mL), mientras que el gradiente de MOI del fago es una dilución seriada de 10 veces la MOI. Las filas y columnas de cada placa están marcadas con letras y números, respectivamente. El gradiente de blanco y negro en cada pocillo refleja la densidad óptica del cultivo y es el valor medio de tres réplicas biológicas. Se analizaron las cepas de SARM cotratadas con oxacilina y **(B)** Evo2 o **(C)** ΦStaph1N para determinar su resistencia a fagos y a la oxacilina. La combinación de letras y números indica el pocillo del que se seleccionaron las células para el análisis. Los pocillos que no produjeron un cultivo viable se etiquetaron como NG (sin crecimiento). En los pocillos que recrecieron, calculamos la eficiencia de formación de placas (EOP) del fago y medimos la reducción en la CMI de oxacilina. Los cultivos que no mostraron placas virales detectables se etiquetaron como resistentes (R).

**Figura 1 – figura suplementaria 1.** **Sensibilidad de las cepas de SARM a los fagos.** Eficiencia de formación de placas de los fagos en SARM252, MW2 y LAC. Los fagos se diluyeron en serie 10 veces y se depositaron sobre las capas superiores de agar de cada cepa.

**Figura 1 – figura suplementaria 2.** **Curvas de crecimiento de cepas de SARM bajo diferentes niveles de infección por ΦStaph1N.** Los cultivos de SARM252, MW2 y LAC se infectaron con ΦStaph1N a la multiplicidad de infección (MOI) indicada. La densidad óptica (OD600) de los cultivos se monitorizó en un lector de platos automatizado. Cada condición se analizó en tres réplicas independientes.

******

**Figura 2 – figura suplementaria 1. Aislamiento y análisis de secuenciación de Evo2. A.** Placas individuales de Evo2 aparecieron en placas más grandes de ΦStaph1N en LAC. Las placas individuales se aislaron y propagaron en cultivo líquido. Evo2 muestra una mejor formación de placas en MW2 y LAC. Los datos de formación de placas en los paneles de la derecha son los mismos que en la Figura 2A. **B.** Evo2 es una forma mutante de ΦStaph1N con una mutación sin sentido en ORF141. La mutación de A a C (marcada con una flecha) en Evo2 convierte la serina 77 de ORF141 en un codón de terminación.

**Figura 2 – figura suplementaria 2. Curvas de crecimiento de cepas de SARM bajo diferentes niveles de infección por Evo2**. Los cultivos de SARM252, MW2 y LAC se infectaron con Evo2 a la multiplicidad de infección (MOI) indicada. La densidad óptica (DO₂₄) de los cultivos se monitorizó en un lector de placas automatizado. Cada condición se analizó en tres réplicas independientes.

**Figura 2 – figura suplementaria 3. La infección por fagos ΦΝΜ1γ6 LAC no induce la pérdida de resistencia a los β-lactámicos. A.** El LAC tratado con ΦΝΜ1γ6 desarrolla resistencia contra ΦΝΜ1γ6, evidenciada por la reducción de la formación de placas de la población parental a la evolucionada. **B.** Las poblaciones de LAC evolucionadas y parentales muestran CMI comparables contra diferentes β-lactámicos y vancomicina.

**

**Figura 2 – figura suplementaria 4. La infección con el fago SATA8505 induce la pérdida de resistencia a la oxacilina. A.** Las cepas de SARM MRSA252, MW2 y LAC tratadas con el fago SATA8585 desarrollan resistencia a SATA8585, evidenciada por la reducción de la formación de placas de la población parental a la evolucionada. **B.** El SARM evolucionado y parental muestra una CMI reducida contra la oxacilina.

**

**Figura 3 – figura suplementaria 1. Tipos de polimorfismos en cepas de SARM tras la infección por fagos o un tratamiento simulado.** Se representan gráficamente los polimorfismos encontrados en un gen con una categoría COG asignada.

******

**Figura 3 – figura suplementaria 2. Eficiencia de formación de placas de Evo2 y ΦStaph1N en cepas MW2 y LAC con disrupciones en los genes *mgrA, arl* y *sarA*.**

**Figura 5 – figura suplementaria 1. Efecto de la infección por fagos en la formación de biopelículas en cepas de SARM.** Los cultivos se infectaron con ΦStaph1N o Evo2. RP62a es una cepa de *S. epidermidis* con capacidad conocida para formar biopelículas, mientras que LM1680 es un derivado de RP62a que ha perdido dicha capacidad.^71,72^ La biomasa de la biopelícula se evaluó mediante tinción con Cristal Violeta. El Cristal Violeta solubilizado se cuantificó midiendo la absorbancia en 600nm. Los valores representan promedios y desviaciones estándar de tres réplicas. La significancia estadística se determinó mediante una prueba t bilateral.

**Referencias**

1. Ippolito, G., Leone, S., Lauria, F. N., Nicastri, E. & Wenzel, R. P. Methicillin-resistant Staphylococcus aureus: the superbug. *International Journal of Infectious Diseases* **14**, S7–S11 (2010).

2. Tomasz, A. The Mechanism of the Irreversible Antimicrobial Effects of Penicillins: How the Beta-Lactam Antibiotics Kill and Lyse Bacteria. *Annu. Rev. Microbiol.* **33**, 113–137 (1979).

3. World Health Organization. *Critically Important Antimicrobials for Human Medicine*. (World Health Organization, Geneva, 2019).

4. Murray, C. J. L. *et al.* Global burden of bacterial antimicrobial resistance in 2019: a systematic analysis. *The Lancet* **399**, 629–655 (2022).

5. Zapun, A., Contreras-Martel, C. & Vernet, T. Penicillin-binding proteins and β-lactam resistance. *FEMS Microbiol Rev* **32**, 361–385 (2008).

6. Liu, C. *et al.* Clinical Practice Guidelines by the Infectious Diseases Society of America for the Treatment of Methicillin-Resistant Staphylococcus aureus Infections in Adults and Children. *Clinical Infectious Diseases* **52**, e18–e55 (2011).

7. Turner, N. A. *et al.* Methicillin-resistant Staphylococcus aureus: an overview of basic and clinical research. *Nat Rev Microbiol* **17**, 203–218 (2019).

8. Barros, E. M. *et al.* Daptomycin Resistance and Tolerance Due to Loss of Function in Staphylococcus aureus *dsp1* and *asp23*. *Antimicrob Agents Chemother* **63**, e01542-18 (2019).

9. Suttle, S. W. Viruses and Nutrient Cycles in the Sea. **49**, (1999).

10. Kortright, K. E., Chan, B. K., Koff, J. L. & Turner, P. E. Phage Therapy: A Renewed Approach to Combat Antibiotic-Resistant Bacteria. *Cell Host & Microbe* **25**, 219–232 (2019).

11. Hatoum-Aslan, A. The phages of staphylococci: critical catalysts in health and disease. *Trends in Microbiology* **29**, 1117–1129 (2021).

12. Oechslin, F. Resistance Development to Bacteriophages Occurring during Bacteriophage Therapy. *Viruses* **10**, 351 (2018).

13. Azam, A. H. & Tanji, Y. Peculiarities of Staphylococcus aureus phages and their possible application in phage therapy. *Appl Microbiol Biotechnol* **103**, 4279–4289 (2019).

14. Gerlach, D. *et al.* Methicillin-resistant Staphylococcus aureus alters cell wall glycosylation to evade immunity. *Nature* **563**, 705–709 (2018).

15. Meyer, J. R. *et al.* Repeatability and Contingency in the Evolution of a Key Innovation in Phage Lambda. *Science* **335**, 428–432 (2012).

16. Bernheim, A. & Sorek, R. The pan-immune system of bacteria: antiviral defence as a community resource. *Nat Rev Microbiol* **18**, 113–119 (2020).

17. Maynard, N. D. *et al.* A Forward-Genetic Screen and Dynamic Analysis of Lambda Phage Host-Dependencies Reveals an Extensive Interaction Network and a New Anti-Viral Strategy. *PLoS Genet* **6**, e1001017 (2010).

18. Oromí-Bosch, A., Antani, J. D. & Turner, P. E. Developing Phage Therapy That Overcomes the Evolution of Bacterial Resistance. *Annu. Rev. Virol.* **10**, 503–524 (2023).

19. Chan, B. K. *et al.* Phage selection restores antibiotic sensitivity in MDR Pseudomonas aeruginosa. *Sci Rep* **6**, 26717 (2016).

20. Berryhill, B. A., Huseby, D. L., McCall, I. C., Hughes, D. & Levin, B. R. Evaluating the potential efficacy and limitations of a phage for joint antibiotic and phage therapy of *Staphylococcus aureus* infections. *Proc. Natl. Acad. Sci. U.S.A.* **118**, e2008007118 (2021).

21. Xia, G. & Wolz, C. Phages of Staphylococcus aureus and their impact on host evolution. *Infection, Genetics and Evolution* **21**, 593–601 (2014).

22. Voyich, J. M. *et al.* Insights into Mechanisms Used by *Staphylococcus aureus* to Avoid Destruction by Human Neutrophils. *The Journal of Immunology* **175**, 3907–3919 (2005).

23. Holden, M. T. G. *et al.* Complete genomes of two clinical *Staphylococcus aureus* strains: Evidence for the rapid evolution of virulence and drug resistance. *Proc. Natl. Acad. Sci. U.S.A.* **101**, 9786–9791 (2004).

24. Kobayashi, S. D. *et al.* Comparative analysis of USA300 virulence determinants in a rabbit model of skin and soft tissue infection. *J Infect Dis* **204**, 937–941 (2011).

25. Deghorain, M. & Van Melderen, L. The Staphylococci Phages Family: An Overview. *Viruses* **4**, 3316–3335 (2012).

26. Land, A. D., Hogan, P., Fritz, S. & Levin, P. A. Phenotypic Variation Is Almost Entirely Independent of the Host-Pathogen Relationship in Clinical Isolates of S. aureus. *PLoS ONE* **10**, e0129670 (2015).

27. Mo, C. Y. *et al.* Type III-A CRISPR immunity promotes mutagenesis of staphylococci. *Nature* **592**, 611–615 (2021).

28. Goldberg, G. W., Jiang, W., Bikard, D. & Marraffini, L. A. Conditional tolerance of temperate phages via transcription-dependent CRISPR-Cas targeting. *Nature* **514**, 633–637 (2014).

29. Baba, T., Bae, T., Schneewind, O., Takeuchi, F. & Hiramatsu, K. Genome Sequence of *Staphylococcus aureus* Strain Newman and Comparative Analysis of Staphylococcal Genomes: Polymorphism and Evolution of Two Major Pathogenicity Islands. *J Bacteriol* **190**, 300–310 (2008).

30. Pincus, N. B. *et al.* Strain Specific Phage Treatment for Staphylococcus aureus Infection Is Influenced by Host Immunity and Site of Infection. *PLoS ONE* **10**, e0124280 (2015).

31. Li, L. *et al.* The Global Regulon *sarA* Regulates β-Lactam Antibiotic Resistance in Methicillin-Resistant *Staphylococcus aureus* In Vitro and in Endovascular Infections. *J Infect Dis.* **214**, 1421–1429 (2016).

32. Zielinska, A. K. *et al.* *sarA* ‐mediated repression of protease production plays a key role in the pathogenesis of  *S taphylococcus aureus*  USA 300 isolates. *Molecular Microbiology* **86**, 1183–1196 (2012).

33. Crosby, H. A. *et al.* The Staphylococcus aureus Global Regulator MgrA Modulates Clumping and Virulence by Controlling Surface Protein Expression. *PLoS Pathog* **12**, e1005604 (2016).

34. Kwiecinski, J. M. *et al.* Staphylococcus aureus uses the ArlRS and MgrA cascade to regulate immune evasion during skin infection. *Cell Reports* **36**, 109462 (2021).

35. Panchal, V. V. *et al.* Evolving MRSA: High-level β-lactam resistance in Staphylococcus aureus is associated with RNA Polymerase alterations and fine tuning of gene expression. *PLoS Pathog* **16**, e1008672 (2020).

36. Bai, J. *et al.* The role of ArlRS in regulating oxacillin susceptibility in methicillin-resistant *Staphylococcus aureus* indicates it is a potential target for antimicrobial resistance breakers. *Emerging Microbes & Infections* **8**, 503–515 (2019).

37. Walker, J. N. *et al.* The Staphylococcus aureus ArlRS Two-Component System Is a Novel Regulator of Agglutination and Pathogenesis. *PLoS Pathog* **9**, e1003819 (2013).

38. Schulthess, B. *et al.* The σ ^B^ -Dependent *yabJ-spoVG* Operon Is Involved in the Regulation of Extracellular Nuclease, Lipase, and Protease Expression in Staphylococcus aureus. *J Bacteriol* **193**, 4954–4962 (2011).

39. Liu, X., Zhang, S. & Sun, B. SpoVG Regulates Cell Wall Metabolism and Oxacillin Resistance in Methicillin-Resistant Staphylococcus aureus Strain N315. *Antimicrob Agents Chemother* **60**, 3455–3461 (2016).

40. Chen, C. *et al.* Characterization of serine acetyltransferase (CysE) from methicillin-resistant Staphylococcus aureus and inhibitory effect of two natural products on CysE. *Microbial Pathogenesis* **131**, 218–226 (2019).

41. Markham, G. D., DeParasis, J. & Gatmaitan, J. The sequence of metK, the structural gene for S-adenosylmethionine synthetase in Escherichia coli. *Journal of Biological Chemistry* **259**, 14505–14507 (1984).

42. Proctor, A. R. & Kloos, W. E. Tryptophan Biosynthetic Enzymes of *Staphylococcus aureus*. *J Bacteriol* **114**, 169–177 (1973).

43. Maidhof, H., Reinicke, B., Blümel, P., Berger-Bächi, B. & Labischinski, H. femA, which encodes a factor essential for expression of methicillin resistance, affects glycine content of peptidoglycan in methicillin-resistant and methicillin-susceptible Staphylococcus aureus strains. *J Bacteriol* **173**, 3507–3513 (1991).

44. Srisuknimit, V., Qiao, Y., Schaefer, K., Kahne, D. & Walker, S. Peptidoglycan Cross-Linking Preferences of Staphylococcus aureus Penicillin-Binding Proteins Have Implications for Treating MRSA Infections. *J. Am. Chem. Soc.* **139**, 9791–9794 (2017).

45. Gardete, S. *et al.* Role of *murE* in the Expression of β-Lactam Antibiotic Resistance in *Staphylococcus aureus*. *J Bacteriol* **186**, 1705–1713 (2004).

46. Xu, Z. J. *et al.* Tryptophanyl-tRNA synthetase from Bacillus subtilis. *Journal of Biological Chemistry* **264**, 4304–4311 (1989).

47. Jaroch, M. *et al.* Alternate routes to mnm ^5^ s ^2^ U synthesis in Gram-positive bacteria. *J Bacteriol* **206**, e00452-23 (2024).

48. Kobayashi, K. *et al.* Comprehensive DNA Microarray Analysis of *Bacillus subtilis* Two-Component Regulatory Systems. *J Bacteriol* **183**, 7365–7370 (2001).

49. Maki, H., McCallum, N., Bischoff, M., Wada, A. & Berger-Bächi, B. *tcaA* Inactivation Increases Glycopeptide Resistance in *Staphylococcus aureus*. *Antimicrob Agents Chemother* **48**, 1953–1959 (2004).

50. Willing, S., Dyer, E., Schneewind, O. & Missiakas, D. FmhA and FmhC of Staphylococcus aureus incorporate serine residues into peptidoglycan cross-bridges. *Journal of Biological Chemistry* **295**, 13664–13676 (2020).

51. Lalaouna, D. *et al.* RsaC sRNA modulates the oxidative stress response of Staphylococcus aureus during manganese starvation. *Nucleic Acids Research* **47**, 9871–9887 (2019).

52. Masalha, M., Borovok, I., Schreiber, R., Aharonowitz, Y. & Cohen, G. Analysis of Transcription of the *Staphylococcus aureus* Aerobic Class Ib and Anaerobic Class III Ribonucleotide Reductase Genes in Response to Oxygen. *J Bacteriol* **183**, 7260–7272 (2001).

53. Jurado, A., Fernández, L., Rodríguez, A. & García, P. Understanding the Mechanisms That Drive Phage Resistance in Staphylococci to Prevent Phage Therapy Failure. *Viruses* **14**, 1061 (2022).

54. Cao, Z., Casabona, M. G., Kneuper, H., Chalmers, J. D. & Palmer, T. The type VII secretion system of Staphylococcus aureus secretes a nuclease toxin that targets competitor bacteria. *Nat Microbiol* **2**, 16183 (2016).

55. Clauditz, A., Resch, A., Wieland, K.-P., Peschel, A. & Götz, F. Staphyloxanthin Plays a Role in the Fitness of *Staphylococcus aureus* and Its Ability To Cope with Oxidative Stress. *Infect Immun* **74**, 4950–4953 (2006).

56. Yarwood, J. M. & Schlievert, P. M. Quorum sensing in Staphylococcus infections. *J. Clin. Invest.* **112**, 1620–1625 (2003).

57. Moller, A. G. *et al.* Genes Inﬂuencing Phage Host Range in Staphylococcus aureus on a Species-Wide Scale. **6**, (2021).

58. Reyes-Robles, T. *et al.* Staphylococcus aureus Leukotoxin ED Targets the Chemokine Receptors CXCR1 and CXCR2 to Kill Leukocytes and Promote Infection. *Cell Host & Microbe* **14**, 453–459 (2013).

59. Cheung, G. Y. C., Bae, J. S. & Otto, M. Pathogenicity and virulence of *Staphylococcus aureus*. *Virulence* **12**, 547–569 (2021).

60. Otto, M. Staphylococcus aureus toxins. *Current Opinion in Microbiology* **17**, 32–37 (2014).

61. Kortright, K. E., Doss-Gollin, S., Chan, B. K. & Turner, P. E. Evolution of Bacterial Cross-Resistance to Lytic Phages and Albicidin Antibiotic. *Front. Microbiol.* **12**, 658374 (2021).

62. Crosby, H. A., Kwiecinski, J. M. & Horswill, A. R. In Vitro Assay for Quantifying Clumping of Staphylococcus aureus. in *Staphylococcus aureus* (ed. Rice, K. C.) vol. 2341 31–36 (Springer US, New York, NY, 2021).

63. Feoktistova, M., Geserick, P. & Leverkus, M. Crystal Violet Assay for Determining Viability of Cultured Cells. *Cold Spring Harb Protoc* **2016**, pdb.prot087379 (2016).

64. Hyatt, D. *et al.* Prodigal: prokaryotic gene recognition and translation initiation site identification. *BMC Bioinformatics* **11**, 119 (2010).

65. Li, H. Minimap2: pairwise alignment for nucleotide sequences. *Bioinformatics* **34**, 3094–3100 (2018).

66. Li, H. *et al.* The Sequence Alignment/Map format and SAMtools. *Bioinformatics* **25**, 2078–2079 (2009).

67. Langmead, B. & Salzberg, S. L. Fast gapped-read alignment with Bowtie 2. *Nat Methods* **9**, 357–359 (2012).

68. Liao, Y., Smyth, G. K. & Shi, W. featureCounts: an efficient general purpose program for assigning sequence reads to genomic features. *Bioinformatics* **30**, 923–930 (2014).

69. Love, M. I., Huber, W. & Anders, S. Moderated estimation of fold change and dispersion for RNA-seq data with DESeq2. *Genome Biol* **15**, 550 (2014).

70. Steinegger, M. & Söding, J. MMseqs2 enables sensitive protein sequence searching for the analysis of massive data sets. *Nat Biotechnol* **35**, 1026–1028 (2017).

71. Christensen, G. D., Simpson, W. A., Bisno, A. L. & Beachey, E. H. Adherence of slime-producing strains of Staphylococcus epidermidis to smooth surfaces. *Infect Immun* **37**, 318–326 (1982).

72. Jiang, W. *et al.* Dealing with the Evolutionary Downside of CRISPR Immunity: Bacteria and Beneficial Plasmids. *PLoS Genet* **9**, e1003844 (2013).
